# Sustainable high-energy-density Li–Mn–O layered cathode via metastable synthetic kinetics

**DOI:** 10.1093/nsr/nwag356

**Published:** 2026-06-11

**Authors:** Yuansheng Shi, Fushan Geng, Dilxat Muhtar, Pengfeng Jiang, Jiaqi Cao, Weixin Chen, Chunzhen Yang, Jun Qi, Wei Tong, Xueyi Lu, Bingwen Hu, Yang Sun, Xia Lu

**Affiliations:** School of Materials, Sun Yat-sen University, Shenzhen 518107, China; Shanghai Key Laboratory of Magnetic Resonance, Institute of Magnetic Resonance and Molecular Imaging in Medicine, School of Physics, East China Normal University, Shanghai 200421, China; School of Materials, Sun Yat-sen University, Shenzhen 518107, China; School of Materials, Sun Yat-sen University, Shenzhen 518107, China; School of Materials, Sun Yat-sen University, Shenzhen 518107, China; School of Materials, Sun Yat-sen University, Shenzhen 518107, China; School of Materials, Sun Yat-sen University, Shenzhen 518107, China; School of Materials, Sun Yat-sen University, Shenzhen 518107, China; Anhui Key Laboratory of Condensed Matter Physics at Extreme Conditions, High Magnetic Field Laboratory, Chinese Academy of Sciences, Hefei 230031, China; School of Materials, Sun Yat-sen University, Shenzhen 518107, China; Shanghai Key Laboratory of Magnetic Resonance, Institute of Magnetic Resonance and Molecular Imaging in Medicine, School of Physics, East China Normal University, Shanghai 200421, China; School of Materials, Sun Yat-sen University, Shenzhen 518107, China; School of Materials, Sun Yat-sen University, Shenzhen 518107, China

**Keywords:** layered Li-rich manganese oxides, metastability, ion-exchange, Li-ion batteries, anionic redox

## Abstract

The electrochemical stability of metastable lithium-rich layered cathodes is intrinsically governed by their synthetic history, yet the correlation between ion-exchange thermodynamics and structural evolution remains elusive. Here, using layered P3-Na_0.6_[Li_0.2_Mn_0.8_]O_2_ as a precursor, the spontaneous ion-exchange synthesis of O3-Li_0.6_[Li_0.2_Mn_0.8_]O_2_ (O3-LLM RT) at room temperature yields one of the most promising layered Li–Mn–O cathodes reported to date in LiPF_6_-based carbonate electrolyte at room temperature. However, elevated synthesis temperatures induce manganese ion migration, which hinders the reversible interlayer migration of Li^+^ into the transition metal layers. This structural impediment triggers irreversible lattice oxygen activation and degrades structural stability (e.g. at 280°C, denoted the high-temperature O3-LLM sample as (O3-LLM HT)). Consequently, the perfect layered O3-LLM RT cathode exhibits a high specific capacity of 250 mAh/g with an excellent cycling stability of 88.1% capacity retention (vs. the 51.9% for O3-LLM HT) after 400 cycles at 2.0–4.8 V, ranking at the top of the Li–Mn–O layered cathodes. These findings provide insights into the design and optimization of metastable materials for high-energy-density batteries.

## Teaser

A kinetically stabilized metastable lithium–manganese oxide cathode is created through spontaneous ion exchange, revealing how metastability can enable high-energy-density batteries with improved long-term durability.

## INTRODUCTION

To date, the demand for high-energy-density lithium-ion batteries (LIBs) has become increasingly urgent for electric vehicles and large-scale energy storage systems [1,2]. However, a major concern is that cathode materials remain a critical bottleneck, restricting the advancement of next-generation LIBs. Considering factors such as cost, environmental impact and sustainability, developing cobalt- and nickel-free cathode materials is essential for future energy storage devices [3,4]. Therefore, Li–Mn–O electrode materials are promising alternatives due to Mn’s low cost and abundance, with layered Li–Mn–O cathodes attracting significant attention in recent decades. Early studies focusing on the traditional layered LiMn^III^O_2_, analogue to the LiCoO_2_, were intentionally designed for Li storage [5]. However, the cooperative Jahn–Teller effect of Mn^3+^ presents significant challenges, making the direct synthesis of layered LiMnO_2_ nearly impossible. Although later studies found that the layered LiMnO_2_ phase can be obtained *via* the ion exchange using a Na*_x_*MnO_2_ precursor [6], the spontaneous migration of Mn ions into the Li layer—leading to its transformation into the spinel phase—greatly accelerates structural degradation, significantly limiting its applicability during cycling [7]. Another attempt to directly synthesize the rock-salt-type Li_4_Mn^III^_2_O_5_ also results in rapid capacity fading [8]. These findings may suggest that the exclusive presence of Mn^3+^ ions in the structure severely undermines electrochemical reversibility.

Furthermore, the lithium-rich layered oxides (LRLOs) with both cationic and anionic redox activities have attracted considerable attention [9,10]. By introducing low-valence Li^I^ (Ni^II^, Co^III^, etc., for typical LRLOs) into the Mn layer, the tetravalent Mn ions can be plausibly stabilized under the principle of charge neutrality. Consequently, the well-known Li-rich phase, viz. the monoclinic Li[Li_1/3_Mn^IV^_2/3_]O_2_ (formed by incorporating 1/3 Li into the Mn layer), has been proposed [11]. This material offers a high theoretical capacity of 458 mAh/g but suffers from structural instabilities, including unfavorable cation migration and oxygen release during cycling [12]. Despite the development of various strategies—such as the stacking fault modulation [13], construction of disordered Li/Mn [14] and Mg substitution [15]—cycling reversibility remains insufficient to meet the demands of high-energy-density LIBs. Compared to the conventional layered cathodes (e.g. LiCoO_2_ and layered nickel–cobalt–manganese oxide (NCM) cathodes), the extra-injection of Li into the TM (transition metal) layers generates significant structural versatility, which, in turn, plays a key role in modulating the electrochemical performance of Li-rich layered cathodes. This structural instability stems from the low Mn concentration combined with excessive Li in the TM layer in Li[Li_1/3_Mn^IV^_2/3_]O_2_, which thermodynamically forms a percolation network that facilitates Li/O ion migration [16]. While reducing Li content in the TM layer is theoretically possible, stabilizing tetravalent Mn ions through the conversion of a Na-deficient layered structure to a Li-deficient one via ion exchange leverages the intrinsic differences and connections between Na and Li layered oxides [17]. Recent reports on Li-rich manganese cathodes [O3-Li_0.6_[Li_0.2_Mn_0.8_]O_2_ (O3-LLM)] with Li deficiencies in the Li slab and ∼20% Li in the TM layer—such as Li_0.700_[Li_0.222_Mn_0.756_]O_2_ [18] and Li_0.6_[Li_0.2_Mn_0.8_]O_2_ (LLM) [19]—demonstrate enhanced structural reversibility during cycling [17]. However, these metastable phases correspond to local minima on the free energy landscape, which are thermodynamically less favorable than the ground-state phase, existing as kinetically trapped states [20] and highly susceptible to structural transformations. Common Li–Mn–O precursors tend to crystallize into the spinel phase (such as LiMn_2_O_4_) upon high-temperature synthesis. Moreover, structural evolution of the layered phase toward the spinel phase [6,7] is frequently observed upon cycling. This dual tendency hints at an intrinsic structural ability of Mn in the layered framework, reflecting its editable local Li/Mn coordination environment and the thermodynamic instability of layered LLM [21]. This suggests that the electrochemically stable region for metastable O3-LLM is extremely limited. Nevertheless, significant opportunities remain for developing high-energy-density and stable cathodes by balancing the metastable structural evolution and Li^+^/e^−^ electrochemistry (anion redox) in this narrow range due to the diverse local structural changes under different synthesis pathways and temperatures.

In this regard, the ribbon-ordered P3-Na_0.6_[Li_0.2_Mn_0.8_]O_2_ (P3-NLM) is synthesized as a model precursor to design advanced O3-LLM cathodes via ion exchange. The real-time observation of Na^+^/Li^+^ exchange during the formation of the perfect layered O3-LLM sample at room temperature (O3-LLM RT) is captured using *in situ* batteries with a LiPF_6_-based carbonate electrolyte. Furthermore, the temperature-dependent structural and cycling stabilities of various O3-LLM materials are revealed using the variable-temperature X-ray diffraction (VT-XRD), ^7^Li solid-state nuclear magnetic resonance (ssNMR), X-ray absorption spectroscopy (XAS) and electrochemical analysis. It is found that at elevated ion-exchange temperatures, the spontaneous migration of Mn ions into the Li slab suppresses Li-ion diffusion back to the TM layer and induces the formation of unfavorable oxygen dimers at high voltages, severely compromising cycling reversibility. As a result, the metastable O3-LLM RT demonstrates exceptional cycling stability within the Li–Mn–O cathode system, effectively utilizing both reversible cation and anion redox reactions. These findings provide critical insights into the synthesis and electrochemical behavior of high-performance metastable layered cathodes.

## RESULTS AND DISCUSSION

### Real-time observation of O3-LLM synthesis at RT

**Figure 1.**
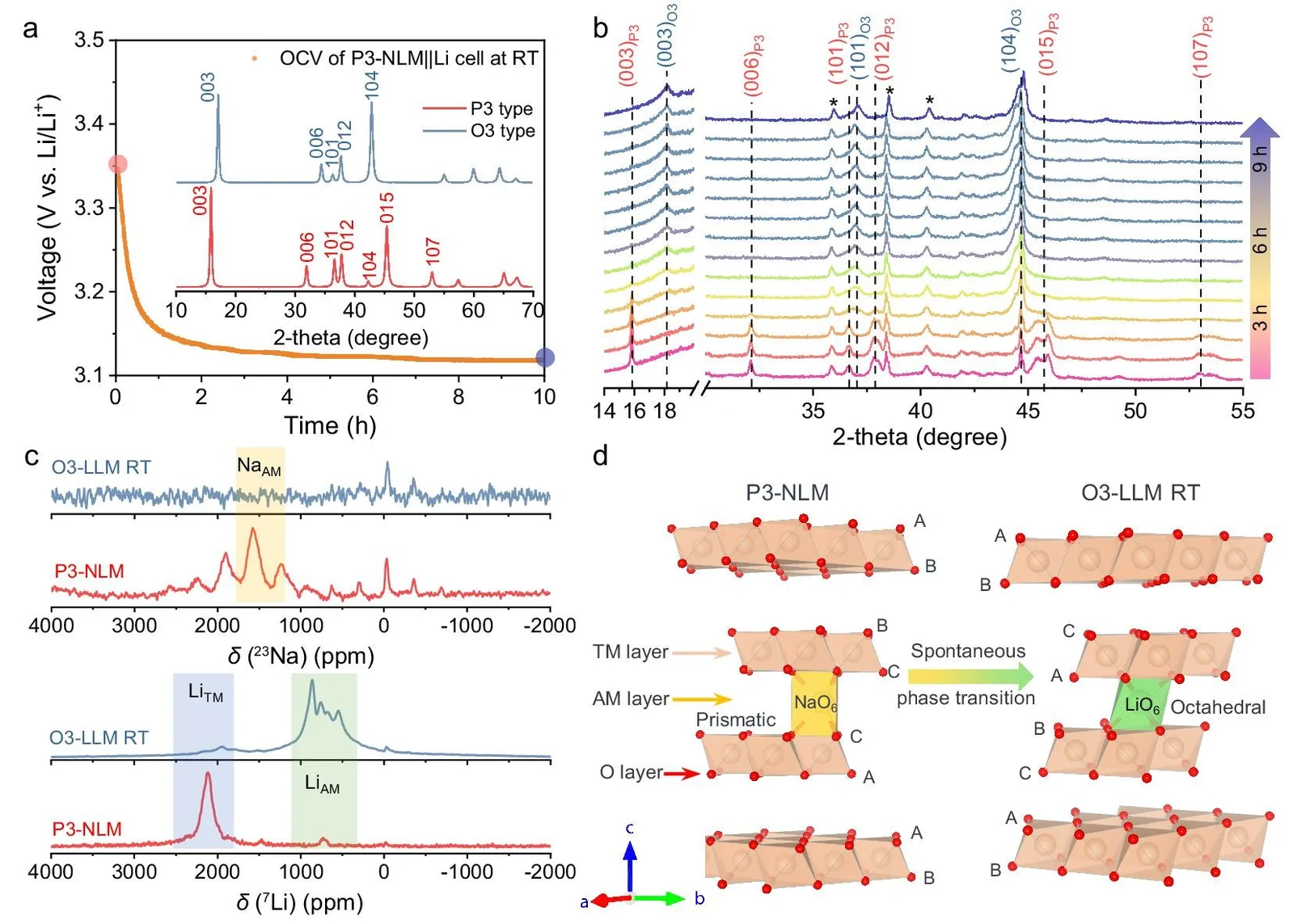
Li^+^/Na^+^ ion exchange from P3-NLM to O3-LLM RT. (a) The OCV of P3-NLM||Li half-cell with time, where the insets are the simulated XRD patterns of P3 and O3 phases. (b) Direct ion-exchange visualizing by *in situ* XRD on P3-NLM||Li half-cell with electrolyte of 1 M LiPF_6_ in ethylene carbonate/dimethyl carbonate (EC/DMC, 1:1 by volume), where the ‘*’ represents the inactive phase of battery components. (c) The ^23^Na (top) and ^7^Li (below) ssNMR isotropic spectra, where the Li_AM_ and Li_TM_ represent the Li^+^ ions, respectively, occupy the alkali metal and TM sites. (d) Schematic illustration of the spontaneous phase transition from P3-NLM to O3-LLM phases *via* ion exchange.

The ribbon-ordered P3-NLM was first prepared as a precursor to perform the ion-exchange process (Fig. S1 and Table S1), where the P3-NLM||Li cell was assembled with LiPF_6_-based carbonate electrolyte to monitor the Li^+^/Na^+^ ion-exchange process using *in situ* XRD. As shown in Fig. 1a, within the first 3 hours, the open circuit voltages (OCVs) of the *in situ* cell quickly drop from 3.35 V to around 3.13 V. The characteristic peak intensities of the P3 phase simultaneously begin to decay once the P3-NLM electrode contacts the electrolyte (Fig. 1b). Referencing the standard Bragg peaks of P3 and O3 phases, the diffraction peaks, such as the intensity of (003)_P3_, (006)_P3_ and (015)_P3_ gradually decline, and the peaks including the (003)_O3_, (101)_O3_ and (104)_O3_ emerge with time to indicate the progressive exchange from original P3 to the final O3-LLM material. After ∼4 hours, the starting P3 phase is plausibly depleted and probably converted into the O3 phase, which demonstrates the underlying spontaneous Li^+^/Na^+^ ion exchange along with the P3 to O3 phase transition (Fig. 1d). During the ion-exchange process, the initial prismatic sites located between the TMO_6_ slabs in the P3 structure cannot stably accommodate the inserted Li ions due to the thermodynamic instability of the Li prismatic configuration. This instability triggers the spontaneous gliding of adjacent TMO_6_ slabs, leading to the formation of the more thermodynamically stable O3 structure, which in turn facilitates the migration of Li ions to the newly formed octahedral sites [22]. Notably, the OCV eventually stabilizes at 3.12 V in response to the structural changes because the quasi-thermodynamic voltage is exclusively dependent on the chemical potential of the atomic structure [23]. Here, the observed voltage drop of 0.23 V closely approaches the standard electrode potential difference of 0.3 V between Li and Na, indicating that the Na^+^/Li^+^ ion-exchange process in sodium layered oxides is predominately driven by thermodynamics. Thus, the obtained product is named O3-LLM RT after the ion exchange at RT. To detect the differences in Li/Na coordination environment before/after soaking into the electrolyte (Fig. 1c), we collected the ^7^Li ssNMR spectra [24,25], which show a significant increase in signal intensity within the 500–1000 ppm range. This increase is attributed to the intercalation of Li^+^ ions into the standard octahedral sites between adjacent TMO_6_ slabs inside the layered O3-LLM [26]. Moreover, after immersing for ∼10 h, no further Na signal is detected from the ^23^Na spectra in the generated O3-LLM RT sample. On the other hand, the absence of Na signal in scanning electron microscope elemental mappings visually confirms the extraction of Na^+^ ions from P3-NLM during the transition to O3-LLM at RT (Figs S2 and S3). Further analysis using ultraviolet-visible absorption spectroscopy (Fig. S4) reveals that the optical bandgap of O3-LLM RT decreases to 0.3 eV compared to ∼1.1 eV of P3-NLM [27]. This seemingly narrow bandgap arises from in-plane TM ion ordering and stacking sequence changes within O3-LLM at RT (Fig. S5).

### Accessible thermodynamic stability of metastable O3-LLM

**Figure 2.**
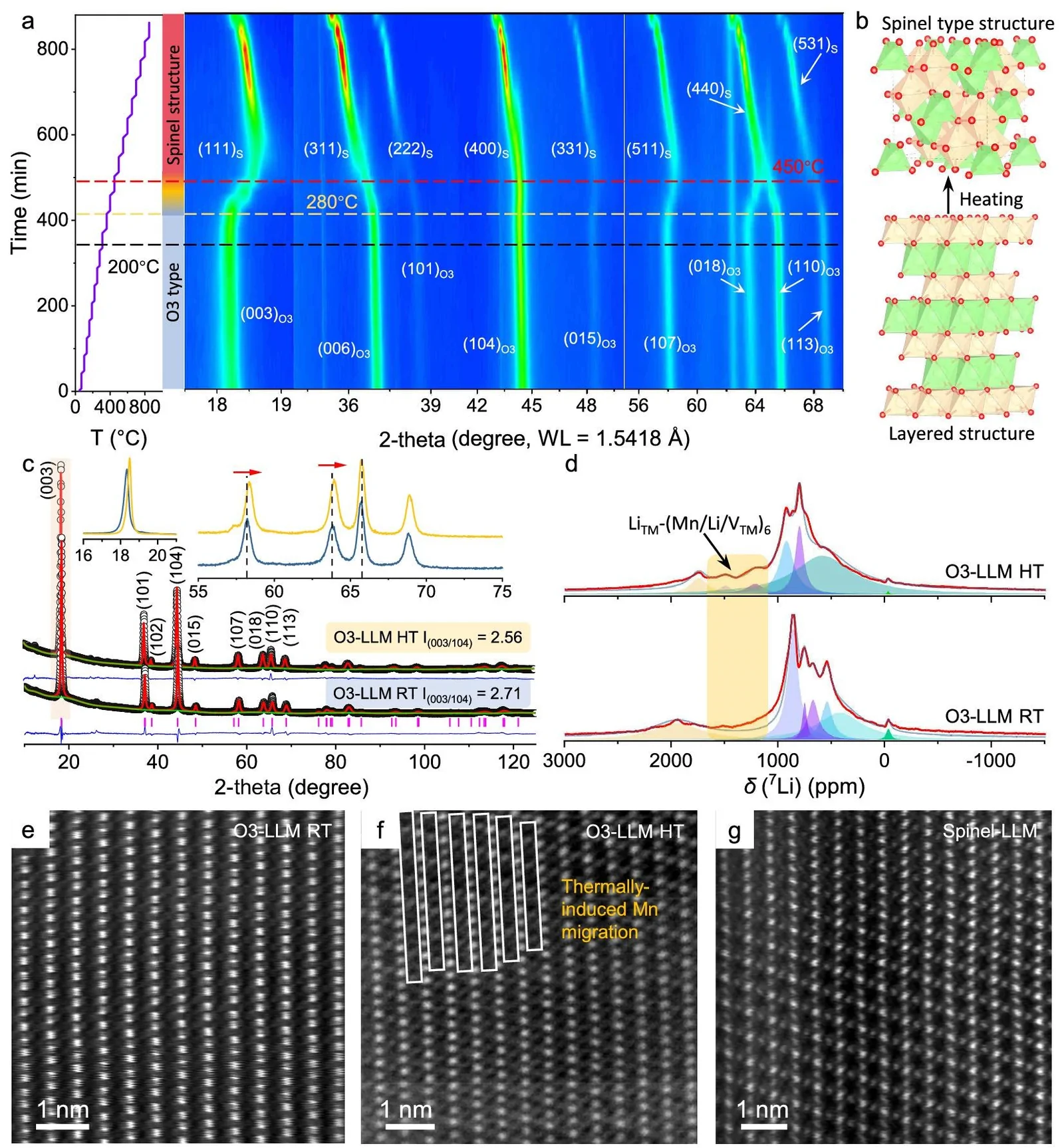
Temperature-dependent stability of metastable O3-LLM. (a) Contour plot of the temperature-dependent XRD patterns of O3-LLM RT. (b) Schematic illustration of the layered to spinel transition of O3-LLM upon heating. (c) Rietveld-refined XRD profiles of O3-LLM RT and HT (*R*$\bar{3}$*m*) materials. (d) The ^7^Li NMR isotropic spectra of O3-LLM RT and HT samples. HAADF-STEM analyses of metastable LLM materials synthesized at (e) RT, (f) 280°C and (g) 450°C. The boxed regions highlight regions of pronounced Mn ion migration.

The VT-XRD was then performed to examine the thermodynamic stability of the as-prepared metastable O3-LLM RT with the temperature increasing from RT to 850°C (Fig. 2a). At temperatures below 200°C, the long-range characteristic XRD peaks of the O3 phase remain unchanged. However, upon heating to around 280°C, a noticeable shift of the (003) peak toward a higher angle begins, and the (018) and (110) peaks gradually approach each other, indicating subtle structural changes within the metastable O3-LLM phase. At 450°C, the (003), (107) and (018) peaks of the O3-LLM sample exhibit a more pronounced rightward shift. Subsequently, the merging of the layered (018)_O3_ and (110)_O3_ peaks into the (440)_Spinel_ peak becomes distinctly observable, indicating the transition to the spinel phase as shown in Fig. 2b and Fig. S6 and Note S1, which corresponds to significant Li/Mn cation mixing during the phase transition. Additionally, the characteristic XRD peaks of the spinel structure undergo a collective shift toward lower angles as the temperature continues to rise, indicating a recrystallization process driven by lithium volatilization at high temperatures. Of particular note is that 280°C is the commonly reported temperature for the eutectic point of LiNO_3_/LiCl molten-salt-assisted ion exchange [18,21,28,29]. In this regard, the high-temperature O3-LLM sample (O3-LLM HT) and Spinel-LLM materials were synthesized at 280°C and 450°C, respectively, as reference samples (dashed lines at 280°C and 450°C in Fig. 2a) to investigate their structural and electrochemical differences with the O3-LLM RT material.

Figure 2c exhibits the refined high-resolution XRD patterns of layered O3-LLM RT and HT. A slight shift of the (003) and (018) peaks toward higher angles is observed, in line with the above VT-XRD results (Fig. 2a), which corresponds to the decreased interslab distances in the layered structure (see refinement and ICP results in Tables S2–S4). The LLM sample heated to 450°C was confirmed to undergo severe Mn migration, approaching the critical point of the layered-to-spinel phase transition, as evidenced by the refinement results in Fig. S7 and Table S5 (denoted as Spinel-LLM). Moreover, the intensity ratio of the (003)/(104) peaks decreases from 2.71 to 2.56, and the (018)/(110) peaks start to overlap with each other, both of which empirically indicate the dynamic, probably cross-layer migration of Mn ions as a prerequisite for the layered-to-spinel structure transition [30] (Figs S8 and S9). Furthermore, the migration of Mn ions is directly scrutinized *via* the ^7^Li ssNMR (Fig. 2d), where the clear extra NMR signals in the range of 1000–1700 ppm reflect minor Mn ion shuttling [26] (Li_TM_-(Mn/Li/V_TM_)_6_) between the alkali and TM layers [31], also supported by the results from Grey *et al.* [32].

Furthermore, the high-angle annular dark-field scanning transmission electron microscopy (HAADF-STEM) images along the [100] zone axis shown in Fig. 2e–g provide a more intuitive visualization of the influence of temperature on the atomic structure of LLM. The O3-LLM RT sample synthesized at RT exhibits a well-defined layered structure, attributed to the mild ion-exchange conditions. However, as the temperature increases, partial Mn migration is observed in the O3-LLM HT sample due to thermal activation, as clearly marked by the boxed regions. Upon further heating to 450°C, the LLM material undergoes a complete transformation into a spinel phase, as evidenced in Figs S6 and S7, where the atomic arrangement displays the characteristic spinel structure. These subtle structural changes may have a significant impact on the electrochemical stability of the metastable O3-LLM HT and Spinel-LLM, which will be discussed later.

### Electrochemistry of O3-LLM electrodes

**Figure 3.**
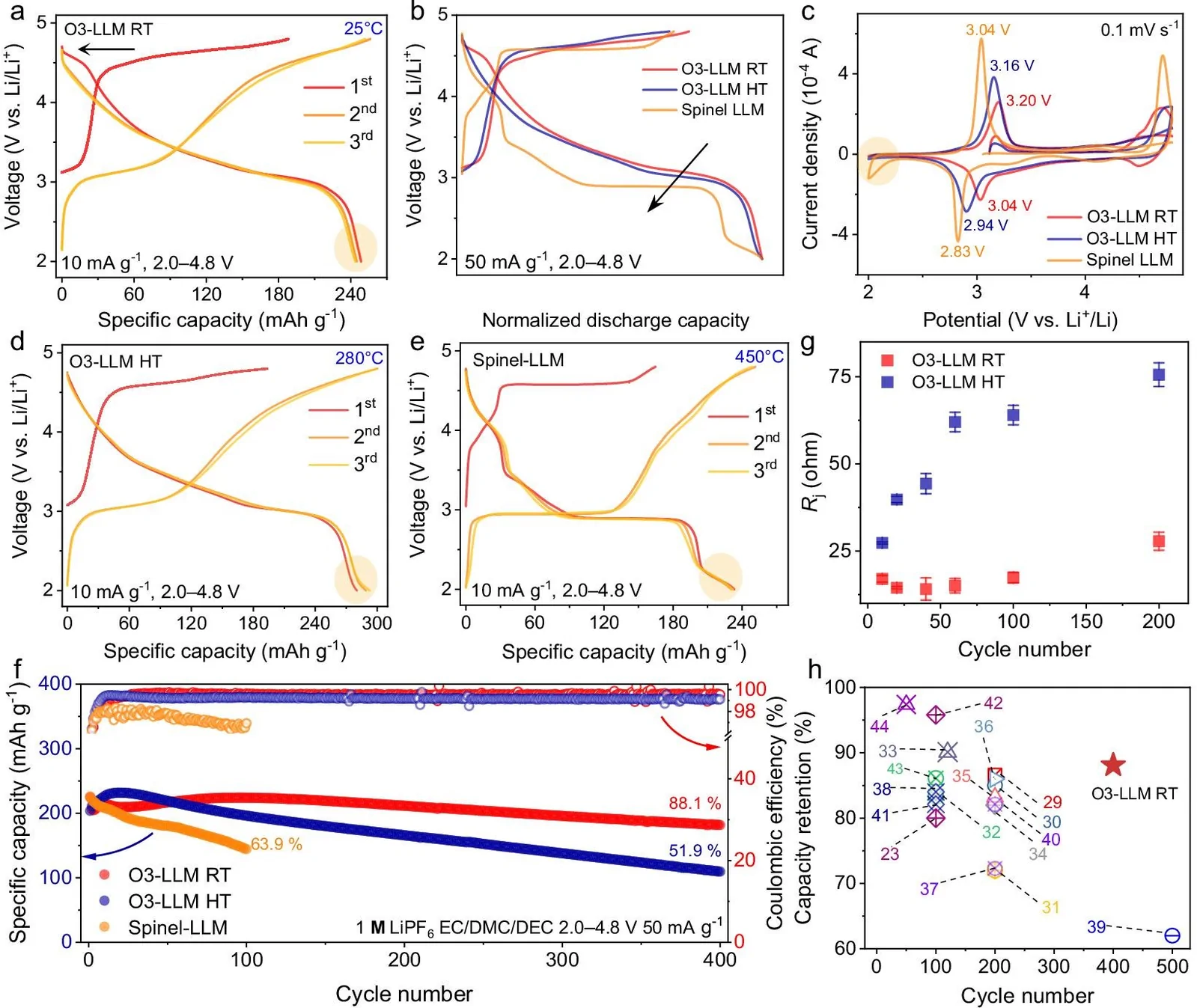
Electrochemical performance of O3-LLM cathodes. (a) Cycling curves of O3-LLM RT in a voltage range of 2.0–4.8 V at a current density of 10 mA/g. (b) Normalized voltage profiles of O3-LLM RT, O3-LLM HT and Spinel-LLM cathodes. (c) CV curves of the initial cycle of O3-LLM RT, O3-LLM HT and Spinel-LLM cathodes at a scan rate of 0.1 mV/s. Cycling profiles of (d) O3-LLM HT and (e) Spinel-LLM cathodes in a voltage range of 2.0–4.8 V at a current density of 10 mA/g. (f) Extended cycling stability of O3-LLM RT, O3-LLM HT and Spinel-LLM cathodes, respectively. (g) The fitted interfacial resistances of *R*_j_ (electrode–electrolyte interface) at different cycles. (h) The capacity retention and cycle number comparison between the O3-LLM RT and the previously reported LRLOs cathodes, where the corresponding references and comparative details are summarized in Table S7.

The electrochemical performance of different O3-LLM materials was tested using a coin-type Li half-cell. Specifically, the O3-LLM RT exhibits a short discharge plateau during the initial discharge (∼20 mAh/g), marked by an arrow (Fig. 3a), resembling the lithiation process reported for P3-NLM [33]. Furthermore, from the normalized voltage curves (Fig. 3b), it is evident that the discharge voltage around 3 V decreases as the synthesis temperature of the LLM samples increases. This trend aligns with the cyclic voltammetry (CV) curves (Fig. 3c), where the redox couple of O3-LLM RT (3.20/3.04 V) shifts to the lower voltages in O3-LLM HT (3.16/2.94 V) and further decreases in Spinel-LLM (3.04/2.83 V). Furthermore, a sloping voltage plateau around 2 V is observed to gradually grow as the temperature increases. All these correlations indicate that the subtle differences in the local structure, as revealed by NMR (Fig. 2d), can lead to different electrochemical profiles and discharge voltages, and the temperature-driven Mn migration is directly associated with the reduction in the redox couple voltage within the O3 lattice framework. When cycled at 10 mA/g, the O3-LLM HT electrode delivers a specific capacity of ∼280 mAh/g within a potential range of 2.0–4.8 V, higher than the 250 mAh/g of O3-LLM RT and the 230 mAh/g of Spinel-LLM (Fig. 3a, d and e). However, the O3-LLM RT cathode demonstrates much better cycling performance than that of the O3-LLM HT cathode using an optimized 1 M LiPF_6_ in ethylene carbonate/dimethyl carbonate/diethyl carbonate (EC/DMC/DEC, 1:1:1 by volume) electrolyte (Fig. 3f), as is also the case with the 1 M LiPF_6_ in EC/DMC (1:1) electrolyte (Fig. S10). The O3-LLM RT cathode delivers a discharge retention of 88.1% after 400 cycles, remarkably outperforming the 51.9% retention of the O3-LLM HT cathode. Moreover, the overall Coulombic efficiency and average discharge voltage of O3-LLM RT are overwhelmingly higher than those of O3-LLM HT during cycling (Fig. 3f and Fig. S11). In addition, regardless of the cutoff voltage during discharge, the O3-LLM RT electrode maintains more stable cycling performance compared to O3-LLM HT (Figs S12 and S13).

Electrochemical impedance spectroscopy was then performed to check the interfacial evolution, where the measured resistance (*R*_j_) of Li^+^ ion diffusion inside the solid–electrolyte interphase increases rapidly in O3-LLM HT, but much more slowly in the O3-LLM RT over cycles (Fig. 3g and Fig. S11). Then, the charge transfer resistance (*R*_CT_) increases in both materials. All these changes of the interfacial resistances demonstrate a strong correlation with the capacity decay process, especially in the O3-LLM HT electrode with a quick interfacial deterioration. When layered LLM transitions to the Spinel-LLM phase, cycling stability declines further, with capacity retention dropping to ∼63.9% after just 100 cycles. Moreover, the Coulombic efficiency falls below 98%, indicating a further reduction in structural reversibility after severe Li/Mn mixing. In a more generalized contrast, the O3-LLM RT electrode demonstrates exceptional cycling stability within the Li–Mn–O cathode system, exhibiting significantly enhanced durability compared to conventional LRLOs. Notably, when cycled under identical conditions, it retains 88.1% capacity retention after 400 cycles—a marked improvement over the traditional O3-Li_1.12_Ni_0.13_Co_0.13_Mn_0.54_O_2_ cathode (only 24% after 400 cycles as reported [34,35]; see Fig. S14 and Table S6). Furthermore, as quantitatively demonstrated in Fig. 3h and systematically compared in Table S7, the O3-LLM RT cathode still outperforms state-of-the-art LRLOs modified by various strategies. These findings provide new insights into the development of Ni- and Co-free Li-rich high-energy-density layered cathodes using metastable Li–Mn–O structures.

### Structural evolution and redox electrochemistry of the O3-LLM cathodes

**Figure 4.**
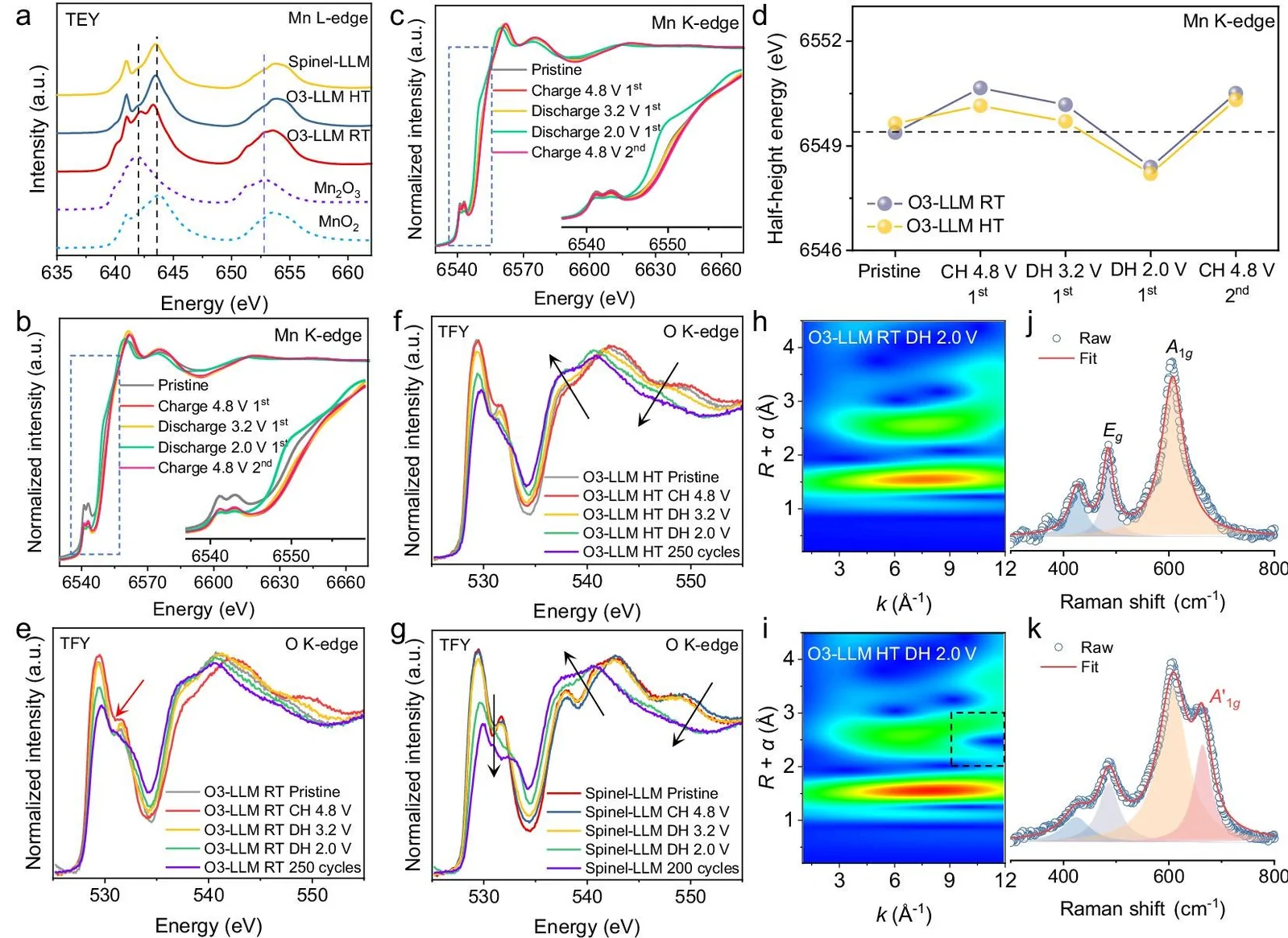
Charge compensation in O3-LLM RT and HT electrodes. (a) The normalized Mn L-edge spectra of O3-LLM RT, O3-LLM HT and Spinel-LLM electrodes at the pristine state in total electron yield (TEY) mode. The normalized Mn K-edge X-ray absorption near-edge structure (XANES) spectra of (b) O3-LLM RT and (c) O3-LLM HT cathodes at different voltages. (d) The Mn K-edge absorption edge energy, determined by the half-height method, for the O3-LLM RT and O3-LLM HT cathodes. O K-edge XAS spectra of (e) O3-LLM RT, (f) O3-LLM HT and (g) Spinel-LLM electrodes at different states in total fluorescence yield (TFY) mode. Contour plots of wavelet-transformed extended X-ray absorption fine structure (WT-EXAFS) at the Mn K-edge (h, i) and *ex situ* Raman spectra (j, k) of O3-LLM RT and HT electrodes after discharge to 2.0 V (*k* denotes the wave vector) [53].

The *in situ* XRD experiments were conducted to further check the structural changes of O3-LLM RT and HT cathodes upon cycling. As shown in Fig. S15, both cathodes undergo similar O3-O3′-O3 phase transitions upon cycling. During delithiation, the XRD peaks show no significant changes before 4.6 V. However, as the charging state comes to the high voltage plateau, a noticeable decrease in the intensity of the (003) peak can be observed, along with a shoulder peak at ∼19°. This corresponds to the Li-deficient O3′ phase with reduced interlayer *d*-spacing, likely caused by anionic redox-induced structural relaxation and interlayer Li⁺ migration (Note S2). Due to the different synthesis routes, a small amount of Mn ions migrate into the Li layers to shrink the interlayer distance of the metastable O3-LLM HT sample. As a result, the *c* lattice parameter of the RT sample is larger than that of the HT sample. Furthermore, this consistent trend throughout the charge–discharge process implies that the migrated Mn ions in the Li layer appear to be immobile during Li insertion and extraction in O3-LLM HT. Meanwhile, the shifts of (101) and (104) peaks indicate the changes of lattice parameters *a/b*, which are related to the charge transfer from Mn ions within the O3-LLM electrode [10,36]. To further investigate the redox processes of O3-LLM cathodes, both hard and soft XAS measurements were conducted. Following ion exchange and subsequent thermal treatment, the bulk Mn valence state in O3-LLM (RT and HT) as well as in Spinel-LLM cathodes remains close to +4, as confirmed by detailed Mn K-edge XAS analysis (Fig. S16, Table S8 and Note S3). Yet, the total electron yield (TEY) Mn L-edge spectra (Fig. 4a) reveal the presence of mixed Mn^3+/4+^ at the O3-LLM RT surface, consistent with Han *et al.* [37], who reported surface TM reduction and the formation of a Li_0.94_ phase after Li^+^/Na^+^ ion exchange with Na_0.67_CoO_2_. Consequently, the Mn K-edge spectrum in fluorescence mode for the pristine O3-LLM RT electrode shows a slight shift to lower energy (Fig. 4b), reflecting the presence of slightly reduced Mn species near the surface. Upon cycling, the Mn ions in O3-LLM RT and HT electrodes participate in charge compensation through reversible Mn^3+/4+^ changes in their oxidation states (Fig. 4b and c). Figure 4d summarizes the Mn K-edge X-ray absorption near-edge structure (XANES) edge position [38], determined at half maximum height. The edge position variations of both samples follow the same sequence of electrochemical redox reactions and are consistent with the TEY Mn L-edge results in Fig. S17. During charging, Mn remains largely in the +4 state. Upon discharge to 3.2 V, the electron paramagnetic resonance (EPR) signal broadens slightly, accompanied by a small shift of the edge position. After further discharge across the ∼3.0 V plateau to 2.0 V, soft XAS, EPR, and edge-shift data consistently confirm the presence of Mn^3+^. These results indicate that the ∼3.2/3.0 V CV redox couple (Fig. 3b and c) arises from the Mn^3+^/Mn^4+^ redox process, which is further supported by *operando* EPR spectra (Figs S18 and S19 and Notes S4 and S5).

Furthermore, Fig. 4e–g present the O K-edge XAS of O3-LLM (RT and HT), Spinel-LLM electrodes in the bulk sensitive total fluorescence yield (TFY) mode [39]. The pre-edge feature, commonly attributed to electronic transitions from the O 1*s* orbital to the TM 3*d*-O 2*p* hybridized states [40–43], is often used as an indicator of covalency and oxygen redox activity [44]. Special attention is given to the feature around 531 eV. Upon charging to 4.8 V, a distinct increase in the pre-edge feature around ∼531 eV is observed (indicated by the arrow in Fig. 4e), which corresponds to the delithiation-induced charge compensation by oxidized oxygen states. Meanwhile, there is a quick intensity decrease in the EPR signal at ∼4.8 V in Fig. S18, signifying the known oxygen redox reaction [18,33]. Upon subsequent discharge, the ∼531 eV feature gradually diminishes and closely resembles the pristine spectrum, suggesting a largely reversible oxygen redox process in the O3-LLM RT and HT samples. However, a slight decrease in the pre-edge intensity is still observed. Notably, after extended cycling, the Spinel-LLM sample exhibits a pronounced suppression of the pre-edge ∼531 eV peak after 200 cycles, indicating a substantial loss of lattice oxygen (Table S9). Consistently, the Mn K-edge extended X-ray absorption fine structure (EXAFS) data reveal a progressive decrease in the Mn–O/Mn–TM intensity ratio across the electrodes in the order of O3-LLM RT, O3-LLM HT and Spinel-LLM electrodes after long-term cycling (Fig. S20), highlighting a correlation between oxygen depletion and structural changes in the Mn–O environment. Moreover, the O3-LLM HT and Spinel-LLM electrodes display significant shifts in the spectral features within the 535–550 eV region (as marked by arrows in Fig. 4f and g). The observed trends in spectral evolutions in the O3-LLM HT and Spinel-LLM, particularly the lattice oxygen loss and the progressive decrease in Mn–O/Mn–TM intensity ratio of EXAFS data, closely mirror the long-term electrochemical performance degradation shown in Fig. 3f, indicating that oxygen redox reversibility progressively diminishes from O3-LLM RT to HT to Spinel-LLM.

Figure 4h and i present the contour plots of the wavelet-transformed (WT) Mn K-edge EXAFS spectra for the O3-LLM RT and HT cathodes. Two primary features, located at ∼1.5 and 2.5 Å, correspond to Mn–O and Mn–TM (TM = Mn or Li) coordination shells, respectively. O3-LLM RT shows negligible changes in Mn–TM coordination after the formation cycle, aligning with its stable electrochemical performance (Fig. 3f). In contrast, the O3-LLM HT electrode exhibits a clear splitting of the Mn−TM scattering peak (highlighted by the black dashed box), along with splitting of the (101) and (104) diffraction peaks in *ex situ* XRD patterns (Fig. S20, Note S6). These features collectively suggest a disproportionation reaction of Mn^3+^ ions, inducing the unfavorable lattice distortions and Jahn–Teller effect. In addition, after discharging to 2.0 V, significant differences in the *ex situ* Raman spectra of the two samples appear (Fig. 4j and k and Fig. S21). The *A*'_1*g*_ peak (defective *A*_1*g*_ mode) at ∼664 cm^−1^ remains present in the O3-LLM HT electrode, indicating the existence of TM_Li_–V_TM_ (V: vacancy) antisite defects induced by Mn migration [45,46]. These results mean that the structural evolution and oxygen redox of O3-LLM RT and HT cathodes go in totally different directions: sustainable evolution for O3-LLM RT versus deteriorated evolution O3-LLM HT after the formation cycle.

### Local structural evolution

**Figure 5.**
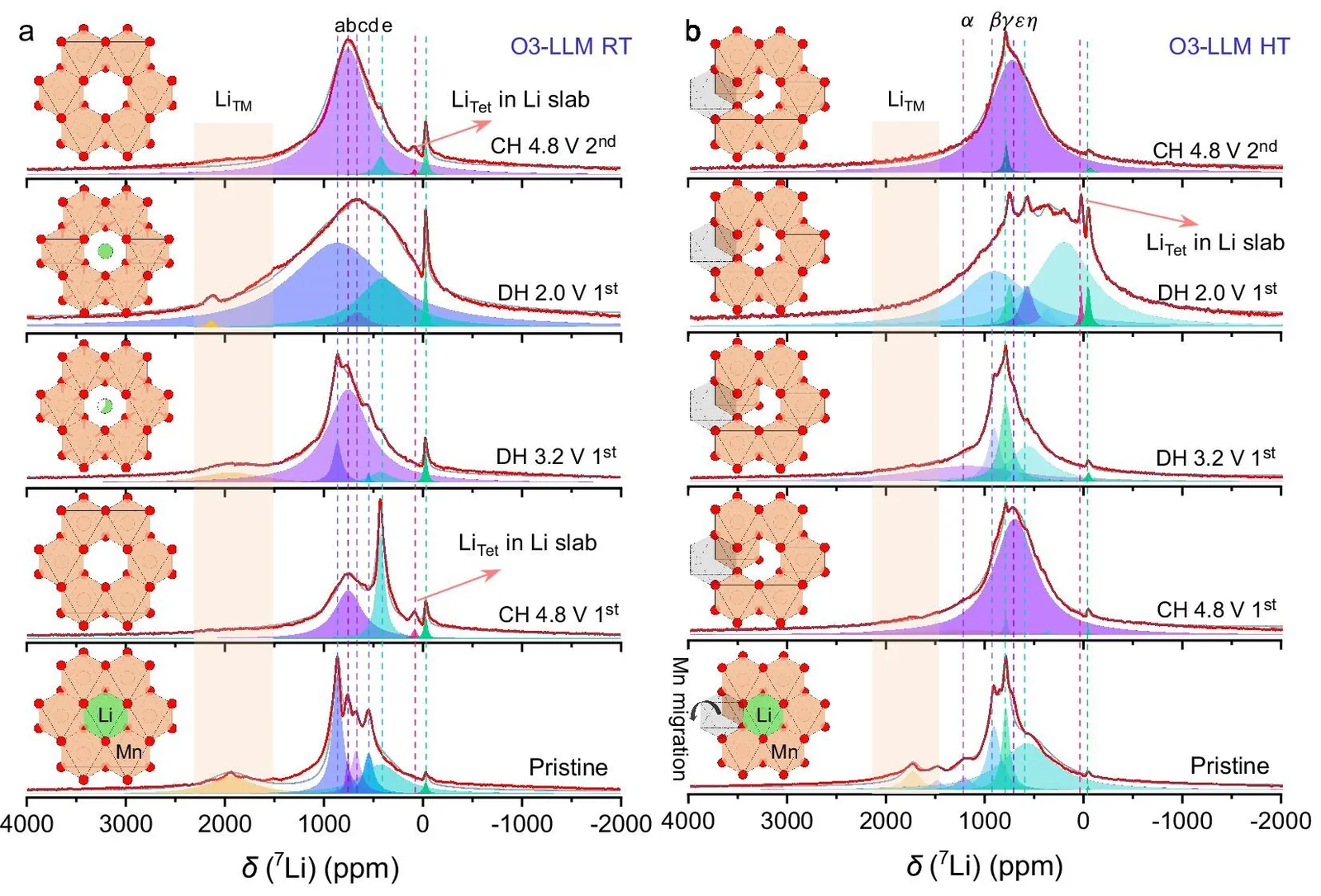
Local structural evolution of O3-LLM cathodes. The ^7^Li NMR isotropic spectra for (a) O3-LLM RT and (b) O3-LLM HT cathodes acquired via the pj-MATPASS sequence. Different Li sites are distinguished according to their respective resonances. The sharp NMR feature around 0 ppm is attributed to diamagnetic species (e.g. Li_2_CO_3_ and LiOH).

The ^7^Li ssNMR isotropic spectra were collected to capture the local structural differences of layered O3-LLM RT and HT samples at short range (Fig. 2d and Fig. 5), which resulted from the lithium intercalation and paramagnetic Mn^4+^ (Mn^3+^) ions [25,26]. The ^7^Li ssNMR spectra of LRLOs typically show two main resonance groups [26,27,47,48], viz. one at 300–1000 ppm attributed to the Li ions in the Li layers (Li_Li_), and the other at 1500–2000 ppm corresponding to the Li ions in the TM layers (Li_TM_). The signal around 100 ppm results from the Li^+^ ion occupation in tetrahedral sites within the Li layers [49]. It is evident that, although the XRD patterns of O3-LLM RT and HT are essentially consistent (Fig. 2c), their ^7^Li ssNMR spectra show significant local structure differences. In particular, the chemical shift of Li_TM_ differs by ∼200 ppm (2000 vs. 1800 ppm), which is related to vacancies and lattice parameter changes caused by Mn ion migration at 280°C. Moreover, the NMR peaks of Li_Li_ range from 800 to 1000 ppm in O3-LLM RT (Fig. 5a, labeled as peaks ‘a’, ‘b’, ‘c’, ‘d’ and ‘e’, respectively), while those are concentrated around ∼700 ppm in O3-LLM HT electrode (Fig. 5b, labeled as peaks ‘*α*’, ‘*β*’, ‘*γ*’, ‘*ε*’ and ‘*η*’, respectively), which clearly displays the local structural changes with temperature. Upon charging, the NMR signal of Li_TM_ ions almost disappears in both O3-LLM electrodes at 4.8 V. It implies that the Li_TM_ ions migrate across the TM layer into the Li layer (Li_Li_), accompanied by the formation of the Li-deficient O′3 phase, which results in the reduced interlayer spacing. However, the dynamic reversibility of Li migration differs significantly between the two samples. In the highly delithiated O3-LLM RT electrode, some Li migrates to Li_Tet_ sites and subsequently returns to the original Li_TM_ sites during discharge, which appears to be highly reversible in the following cycles (Fig. 5a). These observations proved the high structural reversibility of the O3-LLM RT electrode (Fig. 3f) and the smaller voltage fade in the high-voltage region associated with anionic redox (Fig. S22). In contrast, in the highly delithiated O3-LLM HT electrode, there is no Li_Tet_ signal observed at all. Furthermore, no apparent NMR signal of Li_TM_ ions is detected after being discharged to 3.2 or 2.0 V, indicating the immobilized Li^+^ ions in Li_Li_ sites without structural reversibility to their original Li_TM_ sites (Fig. 5b). Consequently, many Li vacancies are left inside the TM layer, which tends to increase the interior strain within the O3-LLM HT structure. The accumulation of such strain can be greatly enhanced by the excessive Li_Li_ ions, which serves as the prerequisite to activate the disproportionation reactions of Mn ions (Jahn–Teller effect of Mn^3+^ ions) and also trigger the structural failure as noted previously (Fig. 4i and k and Fig. S20). Moreover, an additional Li_Tet_ peak emerges in the O3-LLM HT sample at the 2.0 V state. Such a Li_Li_ occupation with the reinserted Li ions further contributes to the complex Li coordination environments of the Li layer in O3-LLM HT at the discharged 2.0 V (Fig. 5b). In addition, from the ^7^Li ssNMR signals, the chemical shifts of different Li sites still appear as the discrete resonance peaks, indicating a certainly ordered atomic structure in pristine O3-LLM RT, while a united bulge appears in O3-LLM HT after charging to 4.8 V. This may explain the voltage plateau related to the lattice oxygen reduction (Fig. 3a) at the beginning of the first discharge [33]. The above results indicate that differences in the metastability of O3-LLM govern the reversibility of Li cross-layer migration upon cycling.

### Thermodynamic origins of O3-LLM failure

**Figure 6.**
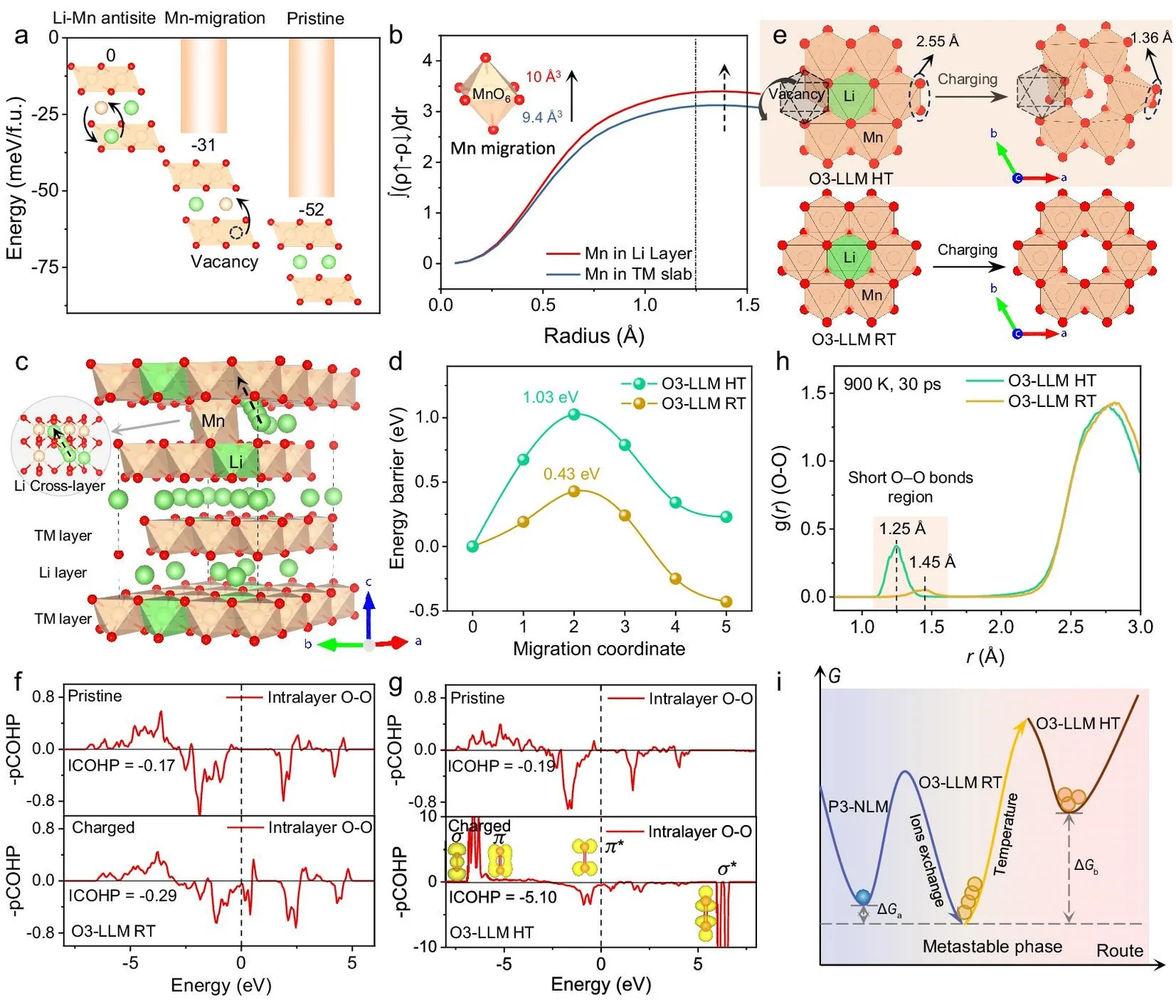
Degradation of O3-LLM cathode. (a) The relative energies of the O3-LLM with Li/Mn antisite, the defective O3-LLM where the Mn ion migrates to the Li layer with a vacancy left (O3-LLM HT) and the metastable O3-LLM (RT). (b) The integrated single spin states of Mn ions in Li slab and TM layer, along with the volume change of MnO_6_ octahedra. (c) Schematic illustration of Li^+^ ion migration back to the TM layer. The inset depicts the migration of adjacent Li ions back into vacancies within the TM layer. (d) The calculated migration energy barriers for Li^+^ ions to diffuse into the TM layer in O3-LLM RT and HT. (e) The O–O bond rearrangements in O3-LLM HT with respect to O3-LLM RT. The projected COHP of intralayer O–O bonding and charged state for (f) O3-LLM RT and (g) HT. (h) RDF of O–O pairs for O3-LLM at RT and HT. (i) Schematic illustration of the Gibbs free energy profiles for the metastable O3-LLM.

To explore the local structural differences in Li and Mn migration for O3-LLM at RT and HT cathodes, different O3-LLM structures, including the perfect layered O3-LLM RT, the defective O3-LLM HT where the Mn ion migrates to the Li layer with a vacancy left and the O3-LLM with Li/Mn antisite (Fig. 6a), are enumerated and calculated using density functional theory (DFT). Concerning the energetically favorable perfect O3-LLM RT, there is only an energy gain of 21 meV per formula unit (f.u.) for the defective O3-LLM-HT with the cross-layer migration of Mn ions at 0 K. Such an energy difference is comparable with the thermal excitation energy [50] of ∼25 meV at RT, implying the possible migration of Mn ions within the perfect O3-LLM RT structure at elevated temperatures as observed in Fig. 2. Figure 6b shows a volume expansion of MnO_6_ octahedra after the Mn migration from TM to Li slabs along with a slight reduction in the oxidation state as evidenced by the spin density integration, showing that the partially reduced Mn ions in the Li layer decrease the interplanar spacing locally inside the O3-LLM HT (Fig. S23 and Note S7). This explains the decrease in interplanar *d*-spacing (TM layers) observed by XRD data (Fig. 2 and Fig. S15). As shown in Fig. 6c, the energy barriers of Li^+^ ion diffusion back to the TM layer before/after Mn migration to the Li layer are calculated using the climbing image nudged elastic band method. For O3-LLM HT, the adjacent Li^+^ ion should jump over an energy barrier of 1.03 eV to the nearby Mn vacancy inside the TM layer. In contrast, for O3-LLM RT, the migration of Li^+^ ions back to their original position overcomes only 0.43 eV (Fig. 6d). Furthermore, the O3-LLM RT structure with the restored Li^+^ ions is energetically more favorable (∼0.5 eV lower), which supports the migration of Li^+^ ions back to the TM layer. This well accounts for the differences in the local structures between O3-LLM RT and HT cathodes, as detected in the *ex situ* ssNMR spectra (Fig. 5). At 4.8 V, all Li ions are probably extracted from the TM layers, leaving abundant cation vacancies, where the clear cation migration occurs during structural relaxation inside O3-LLM HT (Figs 5b and 6e). This migration of Mn to the Li site in the Li layer results in uncoordinated O ions, where the short O–O dimers, such as the peroxo (O_2_)^2−^, superoxo (O_2_)^1−^ and molecular O_2_ species [51,52], with bond lengths in the range of 1.2–1.5 Å are generated around the remaining Mn vacancies in the TM layer. These significant changes in oxygen bonding suggest electronic reconfiguration within the O3-LLM HT material. In contrast, the O3-LLM RT exhibits no significant structural distortions at high voltages (Fig. 6e and Fig. S24). Figure 6f and g compare the crystal orbital Hamilton population (COHP) analysis of the intralayer oxygen ions before and after the migration of Mn to the Li site in the Li layer, which involves the O–O dimerization (1.36 Å) in the delithiated O3-LLM HT cathode. There is a clear semiconductor-to-metal transition from the pristine to the delithiated O3-LLM RT cathode (Fig. 6f), similar to LiCoO_2_ [42]. However, the formation of the O–O dimer (1.36 Å) in O3-LLM HT produces both positive and negative COHP values with ICOHP of −5.1, aligning with the σ*/π* and *π*/*σ* hybridization states, respectively, demonstrating the intricate electronic structures (Fig. 6g). Furthermore, the dynamic differences in local structures between O3-LLM RT and HT samples and their impact on O–O bond evolution at high-voltage states were investigated using *ab initio* molecular dynamics simulations at 900 K (Fig. S25 and Note S8). As shown in Fig. 6h, O3-LLM HT exhibits pronounced local distortions due to vacancy clusters in the transition-metal layer, resulting in much shorter and more fluctuating O–O bonds (∼1.25 Å) compared with O3-LLM RT (∼1.4–2.2 Å). These observations highlight distinct local structural environments and kinetic pathways for oxygen evolution in the two samples. Consequently, Mn ion migration can significantly enhance the formation of unstable oxygen dimers in O3-LLM HT, leading to structural disorder and severe voltage fading during delithiation (Fig. S22) [51]. At lower voltages, inserted Li⁺ ions are trapped in the Li layers in O3-LLM HT, causing interlayer stress and structural distortion that deactivate the cathode. In contrast, reversible Li_TM_ ion migration in O3-LLM RT helps relieve the strain and maintain structural stability. The synthesis of temperature-dependent metastable Li–Mn–O materials can be understood through Gibbs free energy, as illustrated in Fig. 6i. Compared to the stable P3-LLM phase, substituting Li for Na to produce O3-NLM introduces structural transformation and an associated energy difference (Δ*G*_a_), primarily attributed to the chemical potential difference between Na and Li, rendering O3-NLM metastable. Consequently, this energy difference drives the spontaneous Na⁺/Li⁺ ion-exchange process. As revealed by DFT calculations, the Mn migration is easily triggered by thermal excitation within the Li–Mn–O crystal framework, leading to the destruction of the originally perfect layered structure. Therefore, during ion exchange at elevated temperatures, the excessive migration of Mn into Li sites within the Li slab further increases the energy difference (Δ*G*_b_ in Fig. 6i) relative to O3-LLM RT, leading to the formation of the high-temperature O3-LLM HT phase. The variations in Gibbs free energy result in distinct local structural evolution pathways, ultimately undermining structural stability and anionic redox reversibility over subsequent cycles.

## CONCLUSION

In summary, the critical synthesis factors influencing metastable O3-LLM have been systematically explored to achieve improved structural reversibility and excellent energy retention in high-energy-density LIBs. The spontaneous ion exchange from P3-NLM to O3-LLM is clearly observed at RT. The resultant O3-LLM RT and O3-LLM HT demonstrate that, even within the same phase, metastable O3-LLM materials undergo significant local structural alterations at elevated temperatures, leading to markedly different specific capacities and retention. Furthermore, at 450°C, the layered LLM structure completely transforms into a spinel phase, further weakening its electrochemical performance. Advanced characterization techniques, including ssNMR, Raman and XAS, reveal substantial lattice distortions in O3-LLM HT at the end of discharge. As a result, in O3-LLM HT, the Li^+^ ions in the TM layer cannot return to their original sites. Due to Mn ion migration and the formation of substantial vacancies at high voltages, the O3-LLM HT is more prone to unstable O–O dimer formation, leading to bonding rearrangements and a lattice oxygen release process. In contrast, in O3-LLM RT, the Li^+^ ions can reversibly migrate back to the TM layers during discharge with a lower energy barrier of 0.43 eV. The O3-LLM RT electrode exhibits an impressive capacity retention of 88.1% after 400 cycles, significantly outperforming the 51.9% of O3-LLM HT. These insights pave the way for the rational optimization of Ni- and Co-free Li–Mn–O layered oxides, facilitating the development of long-life anionic redox systems for next-generation high-energy-density LIBs.

From a practical perspective, current ion-exchange synthesis methods face scalability challenges due to the use of molten or highly concentrated salt treatments, which increase Li salt consumption, process complexity and cost. Industrial implementation may therefore require the development of more efficient and potentially continuous processing strategies. Consequently, the present results indicate that certain Na layered oxides can undergo Li^+^/Na^+^ exchange in lithium battery electrolytes, where the high ionic mobility and intimate solid–liquid contact provide favorable conditions for ion transport. This suggests a mechanistically plausible pathway for electrolyte-mediated or *in situ* ion exchange during cell formation. While further optimization is required to control the exchange extent and uniformity, such an approach may offer a scalable route to stabilize metastable structures within conventional manufacturing workflows, potentially reducing reliance on separate large-scale molten-salt-processing steps.

## Supplementary Material

nwag356_Supplemental_File
