# Supplementary material for: Sustainable high-energy-density Li–Mn–O layered cathode via metastable synthetic kinetics
Source: Natl Sci Rev. 2026 Jun 11;13(17):nwag356. doi: 10.1093/nsr/nwag356 (PMC13591636; doi:10.1093/nsr/nwag356)
Supplement: Supplementary file 1 [file nwag356_supplemental_file.pdf]

Supplementary Materials for

Sustainable High Energy Density Li-Mn-O Layered Cathode via Metastable Synthetic Kinetics

Yuansheng Shi[1], Fushan Geng[2], Dilxat Muhtar[1], Pengfeng Jiang[1], Jiaqi Cao[1], Weixin Chen[1], Chunzhen Yang[1], Jun Qi[1], Wei Tong[3], Xueyi Lu[1], Bingwen Hu[2], Yang Sun[1] and Xia Lu[1,*]

[1]School of Materials, Sun Yat-sen University, Shenzhen 518107, China;
[2]Shanghai Key Laboratory of Magnetic Resonance, Institute of Magnetic Resonance and Molecular Imaging in Medicine, School of Physics, East China Normal University, Shanghai 200421, China and
[3]Anhui Key Laboratory of Condensed Matter Physics at Extreme Conditions, High Magnetic Field Laboratory, Chinese Academy of Sciences, Hefei 230031, China

∗**Corresponding author.** E-mail: luxia3@mail.sysu.edu.cn

Methods

Materials Synthesis

The P3-NLM (P3-$Na_{0.6}Li_{0.2}Mn_{0.8}O_2$) material was first obtained using the solid-state reaction method. Specifically, the stoichiometrically Na_2CO_3 (Macklin, 99.99 %), Li_2CO_3 (Aladdin, 99.9 %) and MnO_2 (Alfa, 99.9 %) were ball-milled for 24 hours with an appropriate amount of isopropyl alcohol. Then, the resulting mixtures were annealed in a muffle furnace at 700 °C for 15 hours. Approximate 2 wt. % excess of Na_2CO_3 and Li_2CO_3 were added to compensate for the Li and Na element loss upon the annealing process. After that, the mixture of P3-NLM and $LiNO_3$/LiCl (weight ratio: 88:12) was heated up to 280 °C for 4 h, and the product was washed several times with deionized water under vacuum filtration. then dried at 100 ℃ for 12h to get O3-LLM HT. Meanwhile, the metastable O3-LLM RT sample was synthesized *via* a precisely controlled room-temperature ion-exchange protocol. Specifically, the P3-NLM precursor was immersed in 1 **M** $LiPF_6$ EC/DMC (1: 1) electrolyte at room temperature, maintaining a liquid-to-solid ratio of ~ 25 - 30 mL g^{-1}. Following the 24 h spontaneous exchange, the resulting O3-LLM RT powder was collected by filtration, then washed rigorously with anhydrous ethanol to eliminate residual lithium salts and organic solvents, and subsequently dried under vacuum at 60 ℃. The Spinel-LLM cathode sample was obtained by heating the O3-LLM RT at 450 ℃ and holding it for 6 hours.

O3-LNCM preparation

The carbonate precursors for the representative Ni-Co-containing Li-rich Mn-based material $Li_{1.12}Ni_{0.13}Co_{0.13}Mn_{0.54}O_2$ were synthesized via a co-precipitation method. Stoichiometric amounts of sulfate solutions (Ni: Co: Mn = 1:1:3) were continuously pumped into a 50 L continuous stirring tank reactor (CSTR). Simultaneously, Na_2CO_3 and NH_4OH aqueous solutions were also pumped into the

CSTR. During the reaction, the stirring speed was maintained at 250 r/min, the pH value was kept at 8.0, and the reaction temperature was controlled at 60 °C. The resulting precursor powders were filtered, washed several times with deionized water, and then dried in an oven at 120 °C for 12 hours. After thoroughly mixing the carbonate precursors and a specific proportion of Li_2CO_3, the powders were initially heated at 480 °C for 5 hours and then calcined at 800 °C for 12 hours in the air.

***Ex situ* and *in situ* XRD measurements**

The X-ray diffraction (XRD) data were collected on a Bruker D8 ADVANCE X-ray diffractometer (40 kV/40 mA) using a Cu K_α radiation ($\lambda \sim 1.5418$ Å) with the continuous scanning mode in a 2θ range from 10° to 130°. The *in-situ* Swagelok-type cell (Beijing Scistar Technology Co., Ltd.) with beryllium windows was used to monitor the Na^+/Li^+ ions exchange process and structural evolution monitor upon Li extraction/insertion. The constant voltage charging at the 1st cycle minimizes the possible electrochemical polarization. The variable-temperature XRD of the O3-LLM RT testing began at the initial RT scan, with the temperature increased in steps of 30 K at a rate of 5 K/min until reaching 310°C. After that, the increment was adjusted to 50 K until the temperature reached 850 °C. The XRD data were refined using GSAS-II software.[1]

XAS, UV-Vis, SEM, STEM, ICP and Raman Measurements

Hard XAS experiments were conducted at the Mn K-edge on the BL11B beamline at the Shanghai Synchrotron Radiation Facility (SSRF). The white light was monochromatized by a Si (111) double-crystal monochromator and calibrated with Mn foil (K-edge at 6539 eV). Hard XAS spectra with transmission (powder pellet) and fluorescence (electrode films) modes were adopted. Soft XAS measurements were mainly performed on the Soft X-ray Spectroscopy beamline at the Australian Synchrotron ANSTO. The soft XAS data were processed and analyzed using the Quick AS NEXAFS

Tool (QANT).[2] Hard XAS data were processed using Athena, where a linear function was subtracted from the pre-edge region and the edge jump was normalized, and were subsequently fitted using Artemis[3]. The χ(k) function was Fourier transformed (FT) using k3 weighting, and the wavelet transform (WT) was utilized to analyze the extended X-ray absorption fine structure data.[4, 5] The ultraviolet-visible (UV-vis) light absorption spectrum was recorded on a Lambda 900 UV-VIS-NIR spectrometer (PerkinElmer, USA) to obtain the optic bandgaps. The microscopy characteristics of the samples were investigated by TESCAN MIRA4. The HAADF-STEM was conducted using a JEOL ARM-200F transmission electron microscope operated at an acceleration voltage of 200 kV. The instrument is equipped with a spherical aberration (Cs) corrector for the condenser lens system. The system provides an energy resolution of better than 0.5 eV and an image resolution of 0.078 nm. The STEM probe current was set to 17 pA. To reduce background contrast noise and enhance lattice visibility, the HAADF images were Fourier-filtered during post-processing. The ICP-OES was performed using an Avio 200 (PerkinElmer, USA) to determine the changes in Na and Li contents before and after ion exchange. The Raman spectra were obtained using inVia™ confocal Raman microscope (Renishaw) equipped with a 633 nm argon ion laser. Each spectrum was recorded with an exposure time of 20 seconds and accumulated twice.

Solid-State ^{23}Na, ^{7}Li NMR and EPR Spectroscopy

The NMR experiments were conducted on a 9.4 T Bruker Avance III NMR spectrometer utilizing a 1.9 mm triple-resonance HXY MAS probe with a MAS rate of 35 kHz. For 7Li acquisition, a pj-MATPASS pulse sequence with a π/2 pulse length of 1.6 μs was employed, with a recycle delay of 50 ms. Meanwhile, a rotor-synchronized solid echo pulse sequence (π/2–τ–π/2–τ–acq.) with a π/2 pulse width of 2.0 μs was utilized for ^{23}Na acquisition, also with a recycle delay of 50 ms. The ^{7}Li and ^{23}Na

shifts were externally referenced to 1 M LiCl aqueous solution and 1 M NaCl aqueous solution at 0 ppm. Finally, the NMR data was analyzed using the Dmfit software.[6] All electrode samples were sealed into standard EPR quartz tubes in an Ar-filled glovebox. *Ex situ* EPR spectra were then recorded using a Bruker EMX Plus 10/12 spectrometer equipped with a dual-mode microwave cavity and an Oxford ESR910 liquid helium cryostat. The perpendicular mode was employed, utilizing a microwave frequency of 9.4 GHz, a microwave power of 1 mW, and a modulation field amplitude of 2 G.

Operando EPR measurement: the pouch cell consisted of an aluminum current collector with O3-LLM RT as the cathode, a copper mesh embedded with lithium metal as the anode, and a glass fiber separator (Whatman, GF/D) with minimal electrolyte. PET/EVA laminating films were used for packaging. The cathode, separator, and anode were wrapped in Kapton film before being heat-sealed. The assembled cell was connected to fine copper wires and placed in a 10 mm quartz tube. Argon gas (80 ml/min) flowed through the tube to prevent oxygen from permeating the package and oxidizing the lithium metal. Then, the pouch cell, placed within a quartz tube, was aligned with the external magnetic field and fixed in the center of the cavity. A low pass filter was used in the circuit to reduce noise. Continuous wave (CW) EPR spectra were recorded at X-band on a Bruker ELEXSYS E580 spectrometer with a Super High Sensitivity cavity (SHQE-W1) during battery cycling.

Electrochemical Measurements

The cathode slurry was prepared by mixing O3-LLM RT (or directly use the P3-NLM electrode), O3-LLM HT and Spinel-LLM powder, acetylene black (AB), and poly(vinylidene difluoride) (PVDF) in a weight ratio of 8:1:1 and dissolving the mixture in N-methyl-2-pyrrolidone (NMP). This slurry was then spread onto aluminum foil using a doctor blade. The electrode was subsequently dried in a vacuum oven at 100 °C for 12 hours and then cut into circular discs with a diameter of 12 mm and a mass

loading of approximately 2 mg/cm^2. CR2032 coin cells were assembled in an Ar-filled glovebox (MIKROUNA, O_2, and H_2O < 0.1 ppm) using lithium metal (450 μm thick) as the counter electrode and Whatman glass microfiber (GF/D) as the separator. Electrolytes used were 1 **M** $LiPF_6$ in EC/DMC (1:1) and 1 **M** $LiPF_6$ in EC/DMC/DEC (1:1:1), with an addition of 100 μL. After a rest period of 6 hours, cycling tests were performed using the Land CT2001A battery test system (Wuhan LAND Electronics. Ltd., China). Cyclic voltammetry (CV) tests were carried out at an electrochemical workstation (CHI 660D), with the voltage set between 2.0 and 4.8 V and a scan rate of 0.1 mV s^{-1}, The electrochemical impedance spectroscopy was measured on an electrochemical workstation (Princeton Applied Research, PARSTAT MC) with frequency range from 100 kHz to 0.05 Hz with an amplitude voltage of 10 mV, and fitted using the ZView electrochemical impedance software. For *ex-situ* characterizations, the batteries were cycled to specified states of charge and discharge and then disassembled inside the Ar-filled glovebox. The resultant electrodes were cleansed with dimethyl carbonate (DMC), dried in the glovebox overnight, and analyzed using *ex-situ* XRD, ssNMR, ICP, EPR and XAS techniques. All at RT unless specified otherwise.

Computational Details

First-principles calculations were conducted using the Vienna ab initio simulation package (VASP),[7] employing density functional theory (DFT). The projected augmented wave (PAW)[8, 9] method was used to describe the electron–ion interactions, and the exchange-correlation interactions were treated within the generalized gradient approximation (GGA)[10] using the Perdew–Burke–Ernzerhof (PBE) functional. A cutoff energy of 520 eV was applied for the plane wave expansion. Geometry optimizations utilized conjugate gradient minimization until all ion forces were below 0.01 eV/Å per atom. Van der Waals interactions were accounted for via the D3 correction.[11, 12] A Γ-centered k-point

mesh of 4 × 4 × 2 was employed for Brillouin zone sampling. All calculations, including structural relaxation, total energy, and electronic structure, were performed using the GGA+U method, with an effective Hubbard U value of 3.9 eV applied to Mn[13]. Crystal structures were constructed using VESTA[14] software. The lowest-energy 20 configurations at various Li content, based on Ewald energy, were enumerated using the Python Materials Genomics (pymatgen) library[15] for O3-LLM RT and HT. Chemical bonding analyses were conducted using the COHP method[16, 17] implemented in the LOBSTER[18] package. Cross-layer migration barriers of Li ions were calculated using the climbing image nudged elastic band (CINEB) method with four intermediate states.[19, 20] CINEB simulations were considered complete when the force magnitude per atom was below 0.02 eV/Å.

AIMD simulations were further carried out to investigate the evolution of O-O bonding in the highly delithiated $Li_{2/9}Li_{7/9}O_2$ structures (O3-LLM RT and HT), where all Li ions in the Li layers had been removed. Van der Waals interactions were accounted for using Grimme's D3 dispersion correction[11, 12]. The calculations employed a plane-wave energy cutoff of 400 eV and sampled the Brillouin zone at the Γ point, with a time step of 2 fs for integration. For simulations at 900 K[21], the system was heated to the target temperature over 2 ps using velocity rescaling, followed by a 4 ps canonical (NVT)[22] equilibration at constant temperature. Subsequent production runs of 30 ps were used for statistical analysis of the structural evolution. Configurations were recorded along the AIMD trajectories whenever significant local rearrangements were observed, including the formation or breaking of short O-O bonds or displacement of Mn ions from the transition-metal layers.

The O-O bonding analysis

A Python script has been developed to analyze the oxygen-oxygen (O-O) bonding in layered oxide cathode materials using AIMD trajectories from VASP (XDATCAR files). The script first reads all

frames of the trajectory and identifies the indices of oxygen atoms in the system. For each frame, it calculates all O-O distances using periodic boundary conditions and records the first 10 shortest O-O pairs per frame, allowing the focused tracking of the most likely bonding interactions. The script also keeps tracking the minimum O-O distance for each frame, which is used to generate a time series that shows the temporal evolution of potential O-O bonds. To identify bonding events, a threshold distance is applied, and only interactions that persist for a minimum number of consecutive frames are considered significant. All pairwise distance data are stored in CSV files for further inspection. In addition, the script constructs a histogram of all O-O distances across the trajectory, providing a radial distribution function (RDF)-like view, and computes a proper O-O pair PDF by normalizing the counts with respect to the expected distribution in a homogeneous system, averaged over all frames. The output includes time-dependent minimum O-O distances, O-O distance histograms, PDFs, and event tables, which can be used for visualization in tools such as OVITO. These results enable quantitative analysis of O-O bonding probabilities, temporal evolution of bonding events, and spatial correlations between oxygen atoms, providing the detailed insights into bond formation and dynamics in layered oxide cathodes. The source code for the O-O analysis is publicly available at [https://github.com/Yuanshengshi/OO-analysis] to facilitate reproducibility and further research.

Supplementary Figures

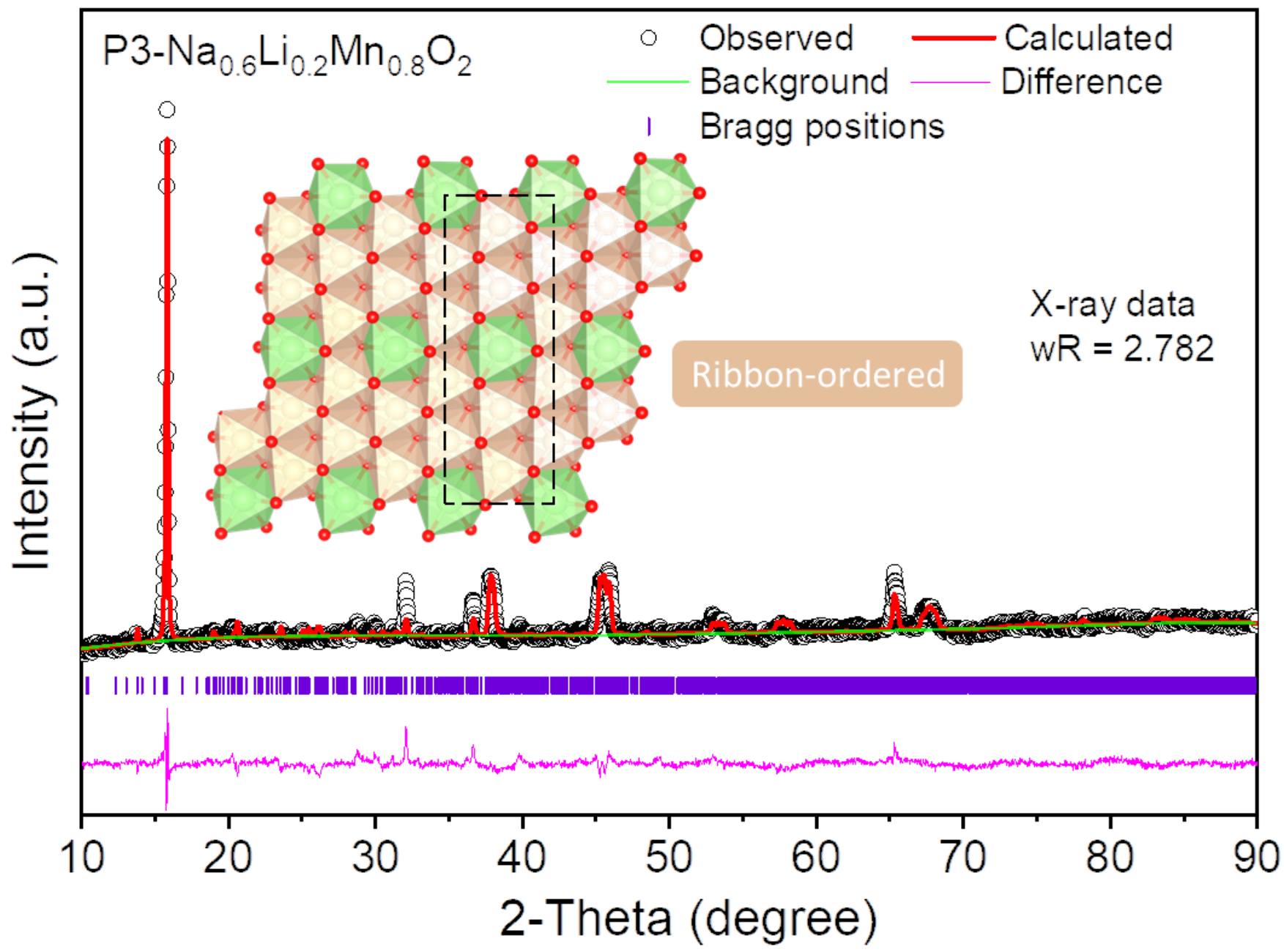

Figure S1. Refined XRD pattern of pristine P3-$Na_{0.6}[Li_{0.2}Mn_{0.8}]O_2$ (P3-NLM) with in-plane ribbon ordering. The superlattice peaks between 10-30° represent the ribbon ordering of Li within the transition metal layer, wherein the experimental (black circles) and calculated (red solid line), the Bragg reflection peaks (purple solid ticks) and the difference curve (magenta line) are shown, respectively.

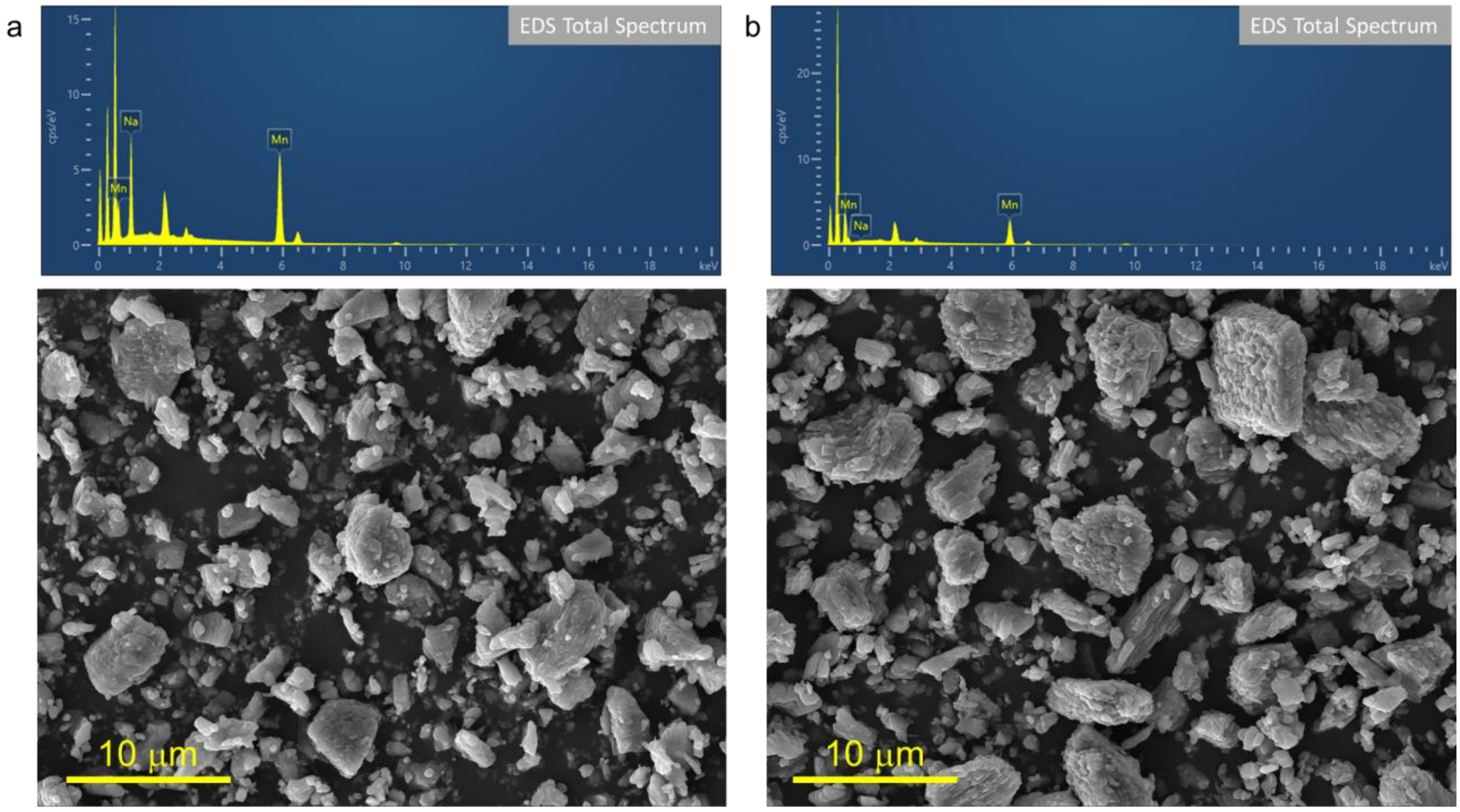

Figure S2. The energy dispersive X-ray spectroscopy (EDS) and SEM images of (a) P3-NLM and (b) O3-LLM RT.

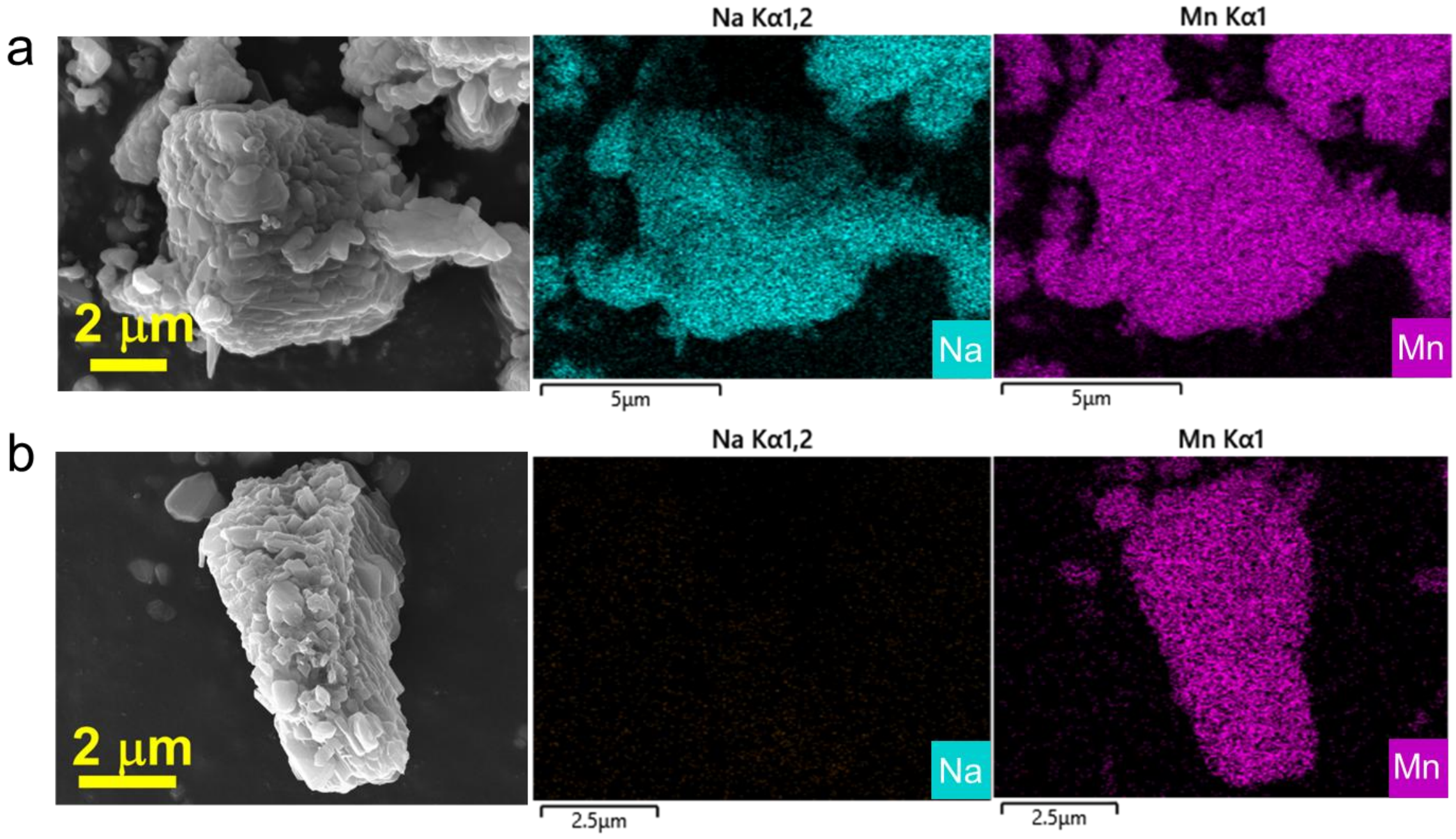

Figure S3. The SEM elemental mapping results of (a) P3-NLM and (b) O3-LLM RT.

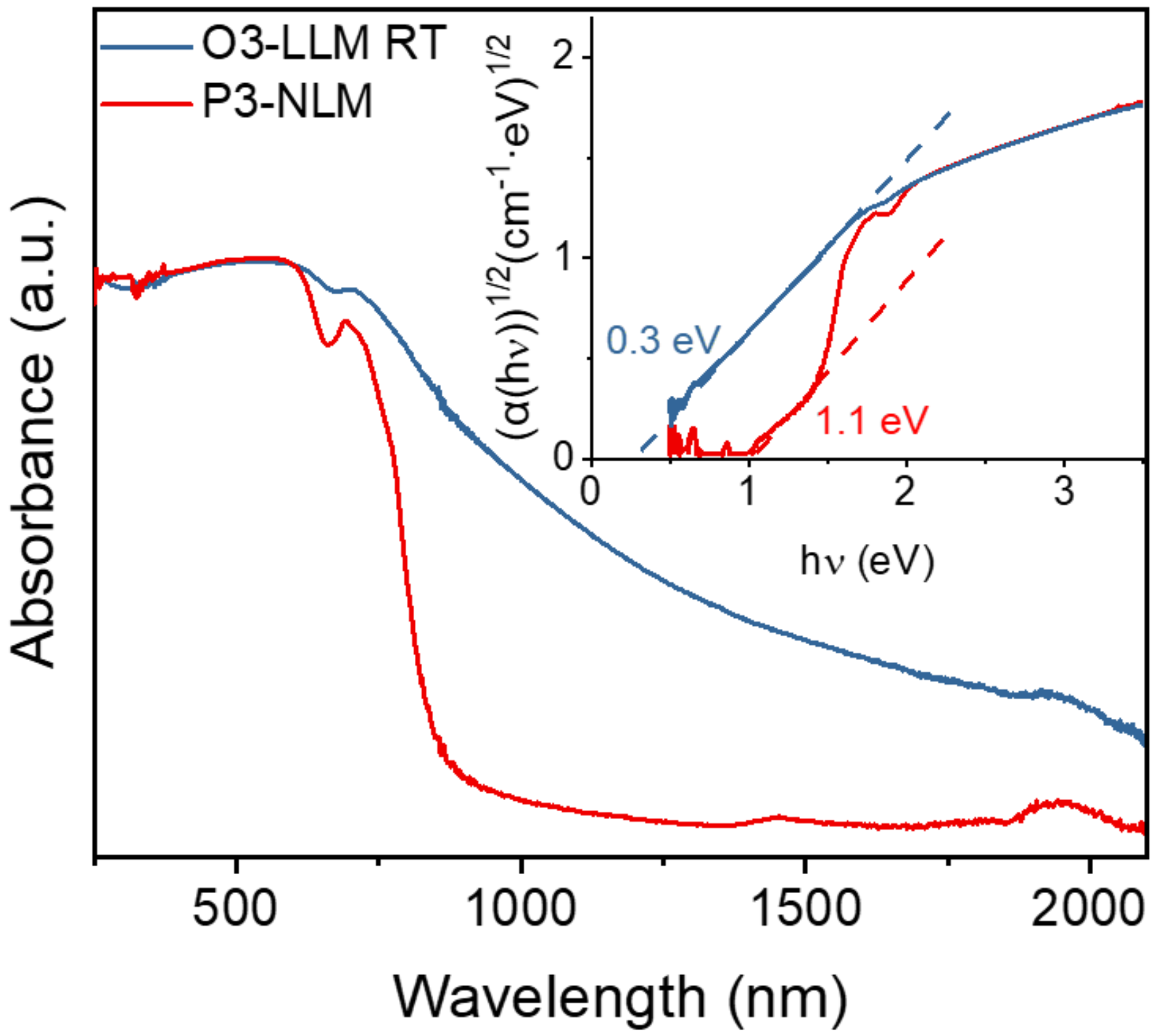

Figure S4. The ultraviolet-visible (UV-Vis.) absorption spectroscopy of P3-NLM cathode before/after soaking in the electrolyte.

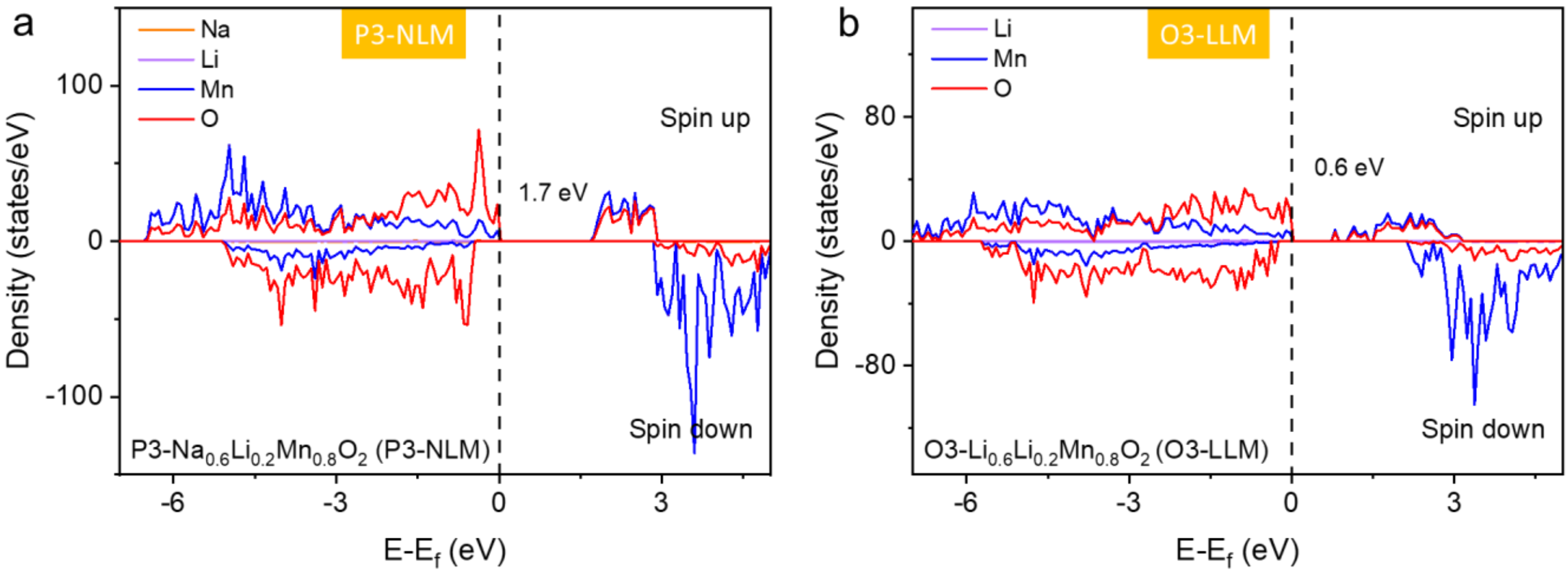

Figure S5.The projected density of states (DOS) for ribbon-ordered P3-NLM and in-plane disordered O3-LLM reveals a significant band gap reduction, decreasing from 1.7 eV in P3-NLM to 0.6 eV in O3-LLM, which is consistent with the observation in $Li_{0.7}[Li_{0.222}Mn_{0.756}]O_2$ cathode.[23]

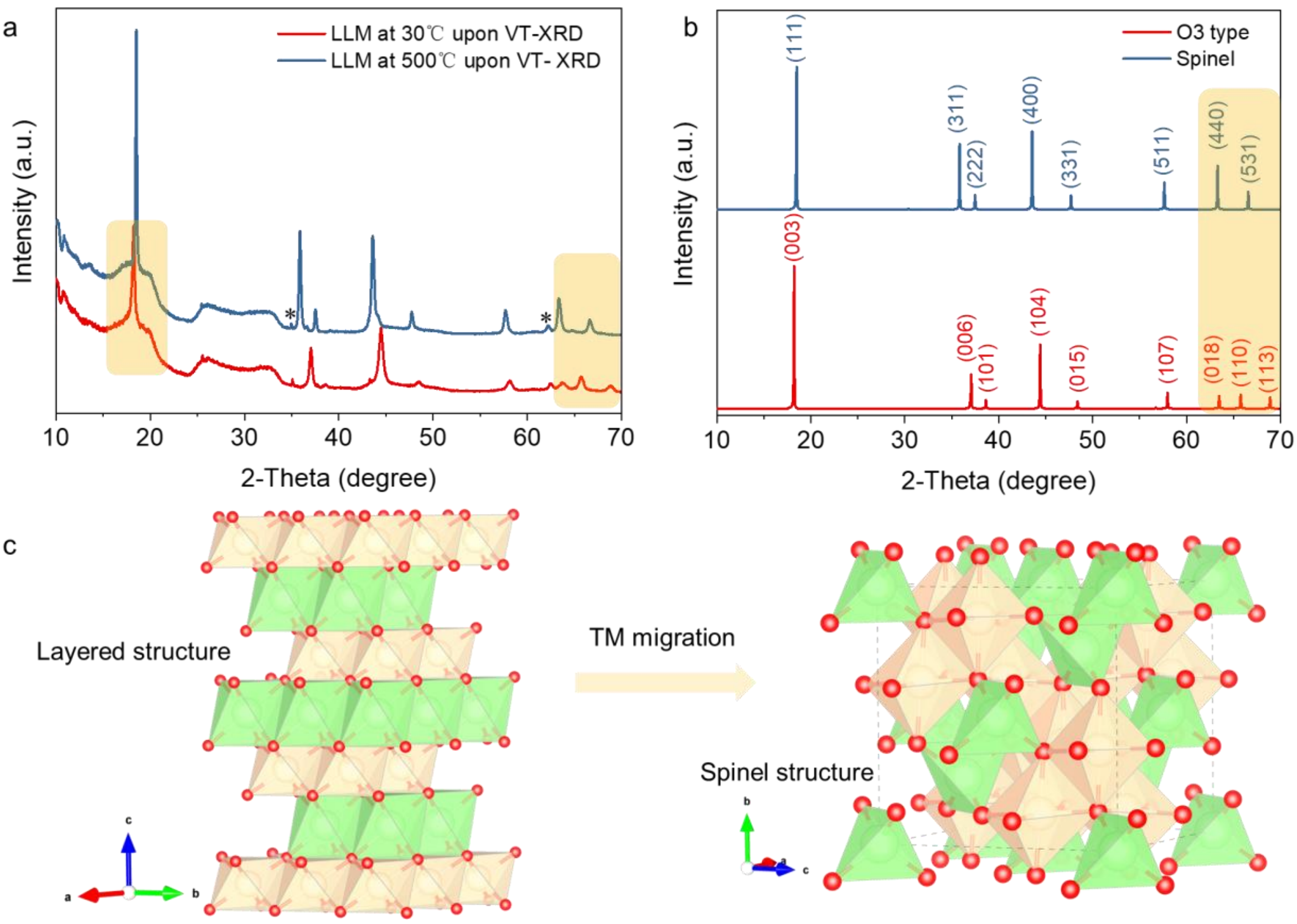

Figure S6. (a) XRD patterns of O3-LLM RT recorded at 30 °C and 500 °C during heating. The yellow-shaded regions highlight the significant shift of the main peak from (003) to (111), as well as the appearance of the Spinel phase (440) peak. Diffraction peaks corresponding to the inactive phase (sample stage) are marked with asterisks (*). (b) Simulated XRD profiles comparing the O3-type layered structure and the Spinel structure. (c) Schematic illustration depicting the transformation of O3-LLM from a layered structure into a Spinel structure upon heating.

Supplementary Note 1.

The changes in the diffraction peaks in the highlighted regions are clearly observed by comparing the experimental XRD data with the simulated XRD results. Specifically, during the transition from the layered structure to the Spinel structure, the main peak (003) shifts to a higher angle and evolves into the (111) peak of the Spinel phase. Similarly, the (018) and (110) peaks of the layered structure gradually merge into a single diffraction peak, corresponding to the (440) peak of the Spinel phase. This transformation occurs progressively; as shown in Figure 2a, the (018) peak shifts to the right and fully merges into the (440) peak only at around 450 ℃ in variable-temperature XRD. This gradual process, occurring over the temperature range of 200-450 ℃, is accompanied by the migration of transition metals, as evidenced by the reduction in the $I_{(003/104)}$ intensity ratio from 2.71 in O3-LLM RT to 2.56 in O3-LLM HT. While no significant changes occur in the long-range structure during this process, substantial alterations in the local structure are evident, which have a pronounced effect on the material's electrochemical performance.

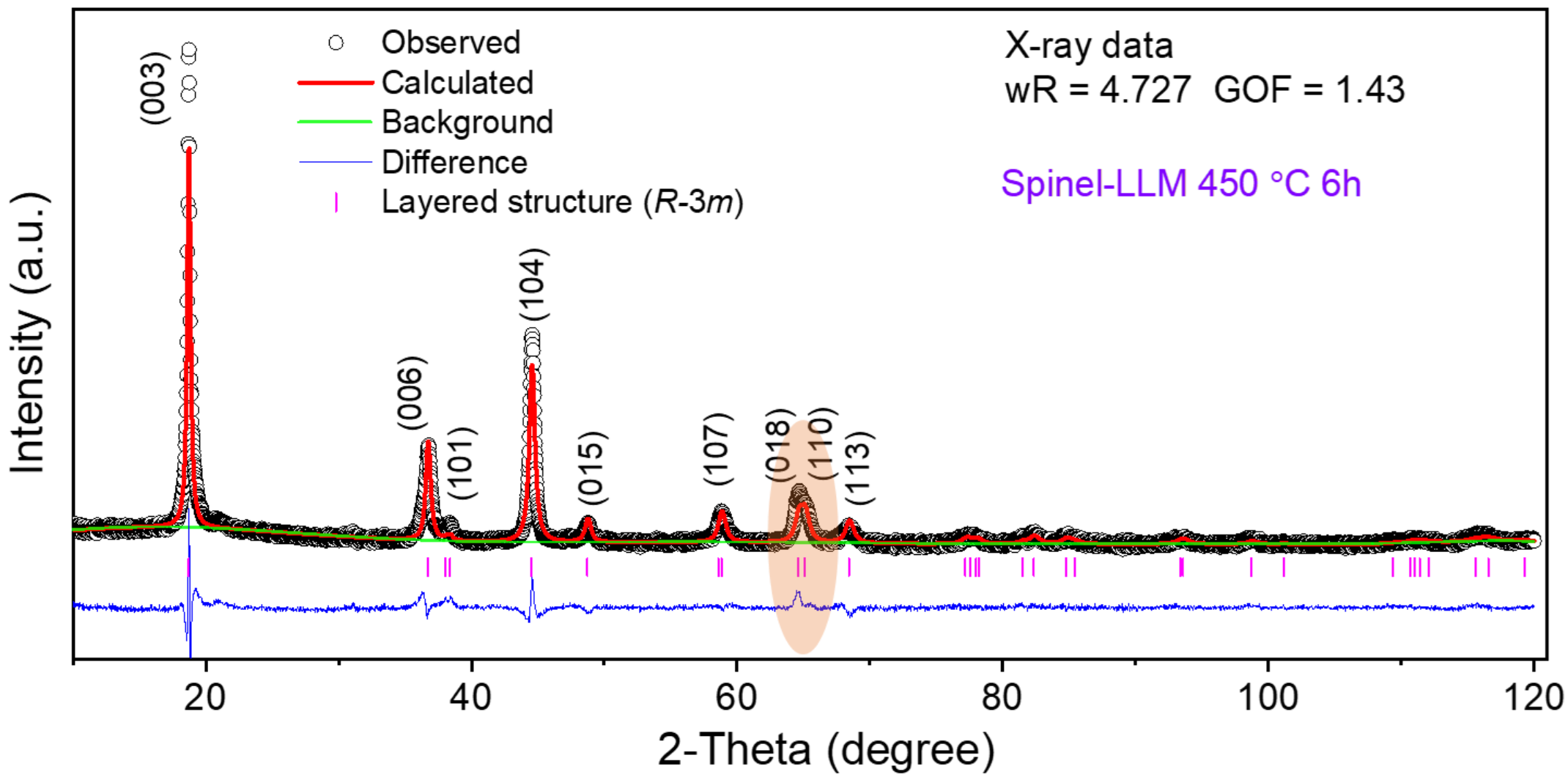

Figure S7. Rietveld refinement of the LLM sample at 450 °C. The significant occupation of Mn ions in the Li slab and the merging of the layered (018) and (110) peaks into a spinel-like (440) reflection indicate the onset of a structural collapse. This marks 450 °C as the critical temperature for the layered-to-spinel phase transformation. While the long-range layered framework is kinetically preserved between 200-400 °C, substantial local metastabilities arise specifically from the thermally induced migration of Mn ions.

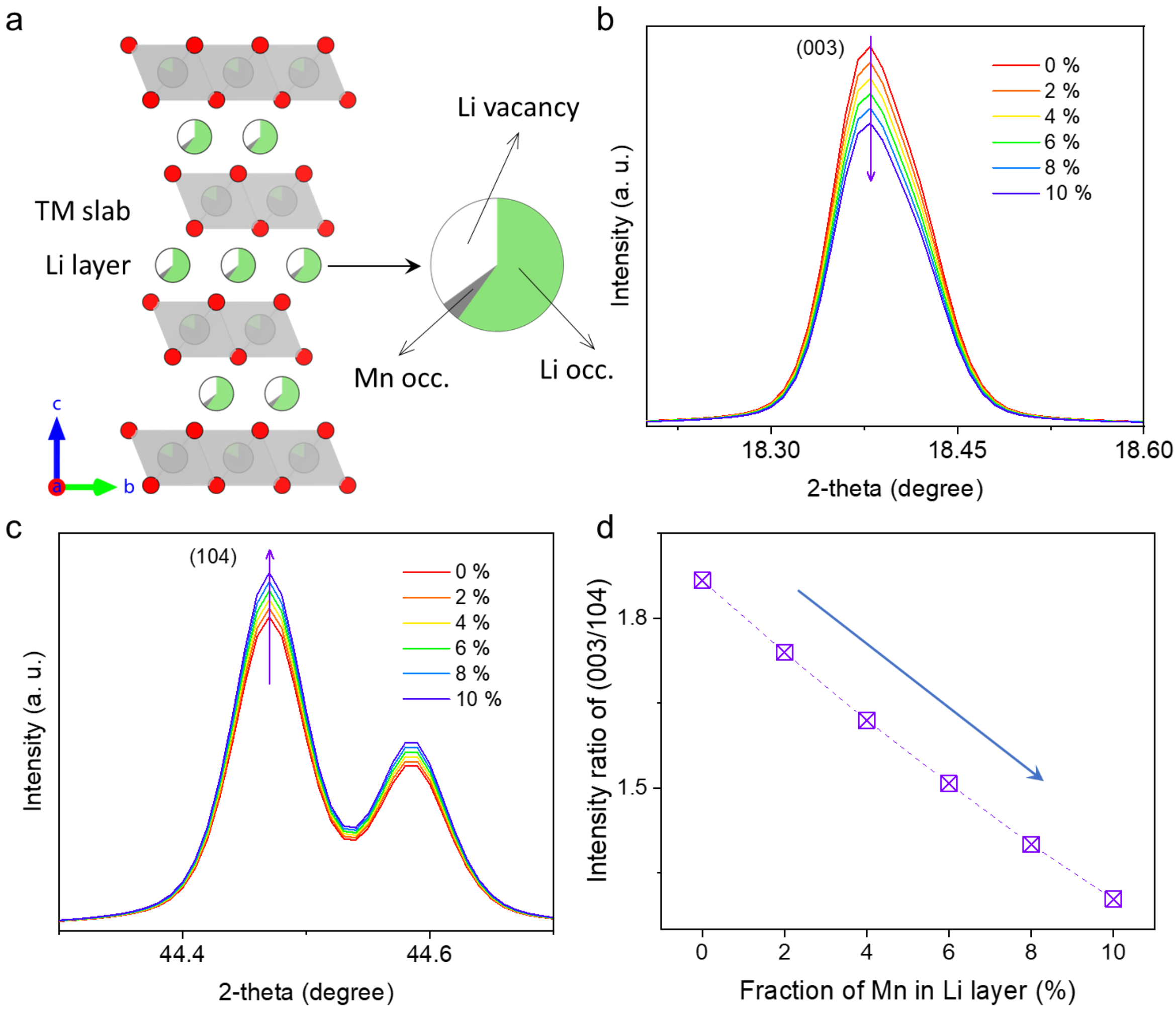

Figure S8. (a) Crystal structures of layered O3-LLM oxides, where the differently colored sectors in the Li layer denote the relative occupancy of Li vacancies, Li, and Mn. Simulated XRD patterns illustrate the migration of Mn ions into the Li layer at varying ratios, with corresponding intensity changes observed in (b) the (003) peak and (c) the (104) peak. (d) The intensity ratio of the (003) to (104) peaks are plotted as a function of the Mn migration proportion into the Li layer. This XRD simulation reveals that a reduction in the (003)/(104) intensity ratio serves as an indicator of Mn migration.

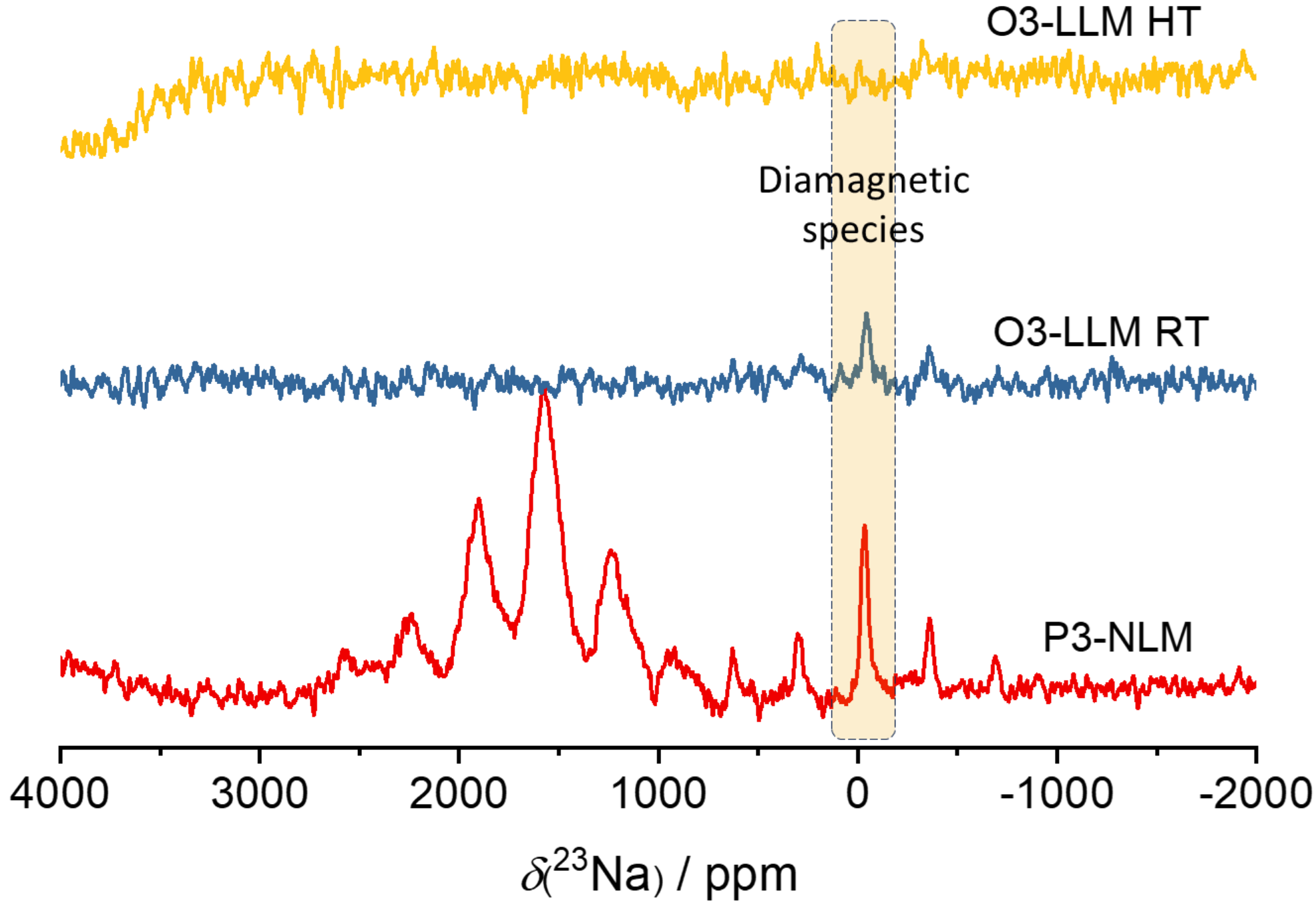

Figure S9. The ^{23}Na MAS NMR spectra of P3-NLM before and after the different ion-exchange pathways. only a weak signal near 0 ppm is observed in the O3-LLM RT sample, likely corresponding to diamagnetic species such as the surface Na_2CO_3. This indicates that the Na detected by ICP-OES primarily originates from surface residues rather than the unexchanged lattice Na, confirming that the Li/Na exchange was complete in all cases.

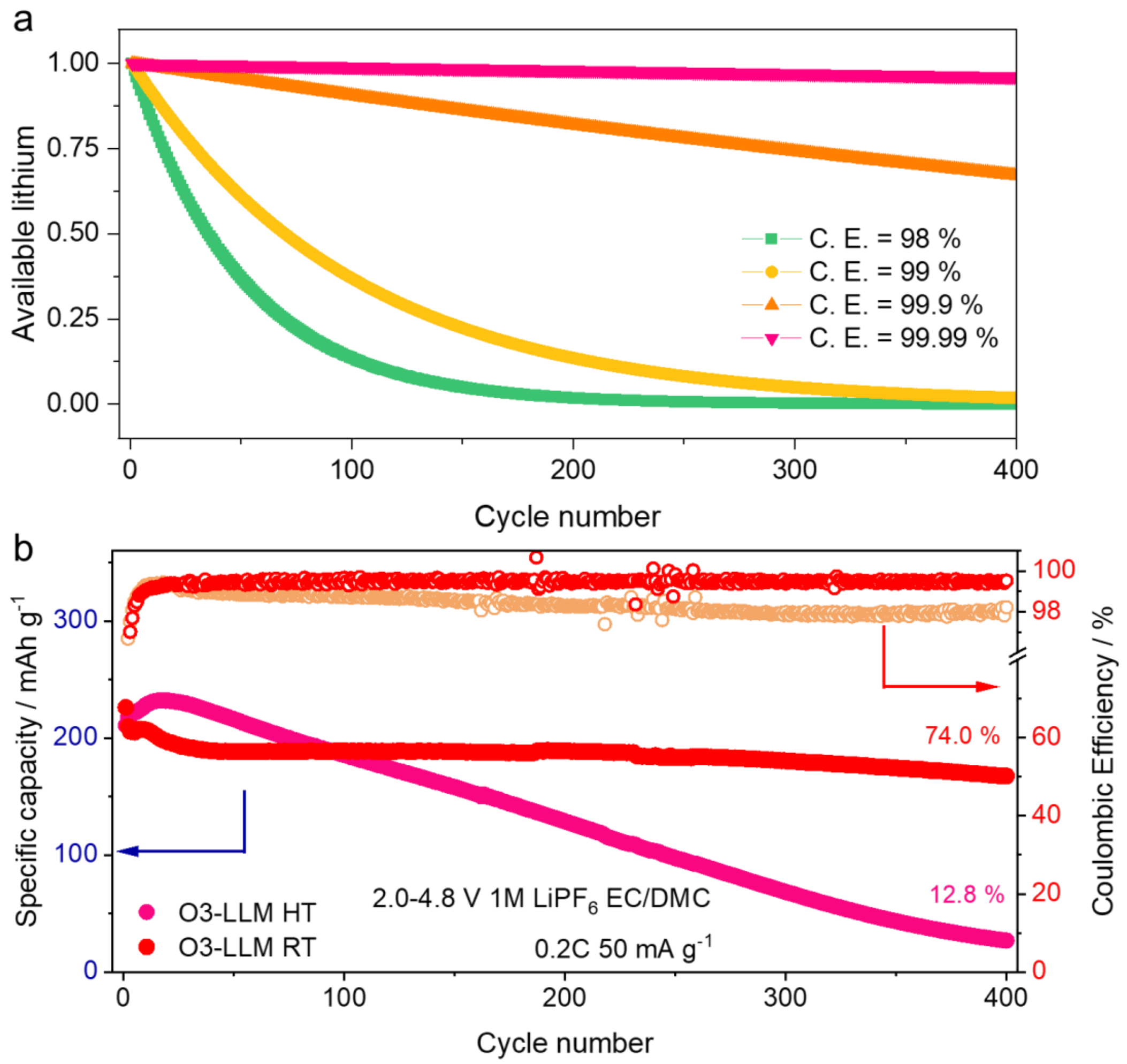

Figure S10. (a) The theoretical cycling performance at different Coulombic efficiencies. The calculation results show that Coulombic efficiency is closely related to the cycling stability and reversibility of electrode materials. To achieve high Coulombic efficiency, minimizing side reactions with the electrolyte and ensuring structural reversibility is essential. (b) Long cycling performance of O3-LLM RT and HT cathodes at the 2.0 – 4.8 V with electrolytes of 1 **M** $LiPF_6$ in EC/DMC (1:1), respectively.

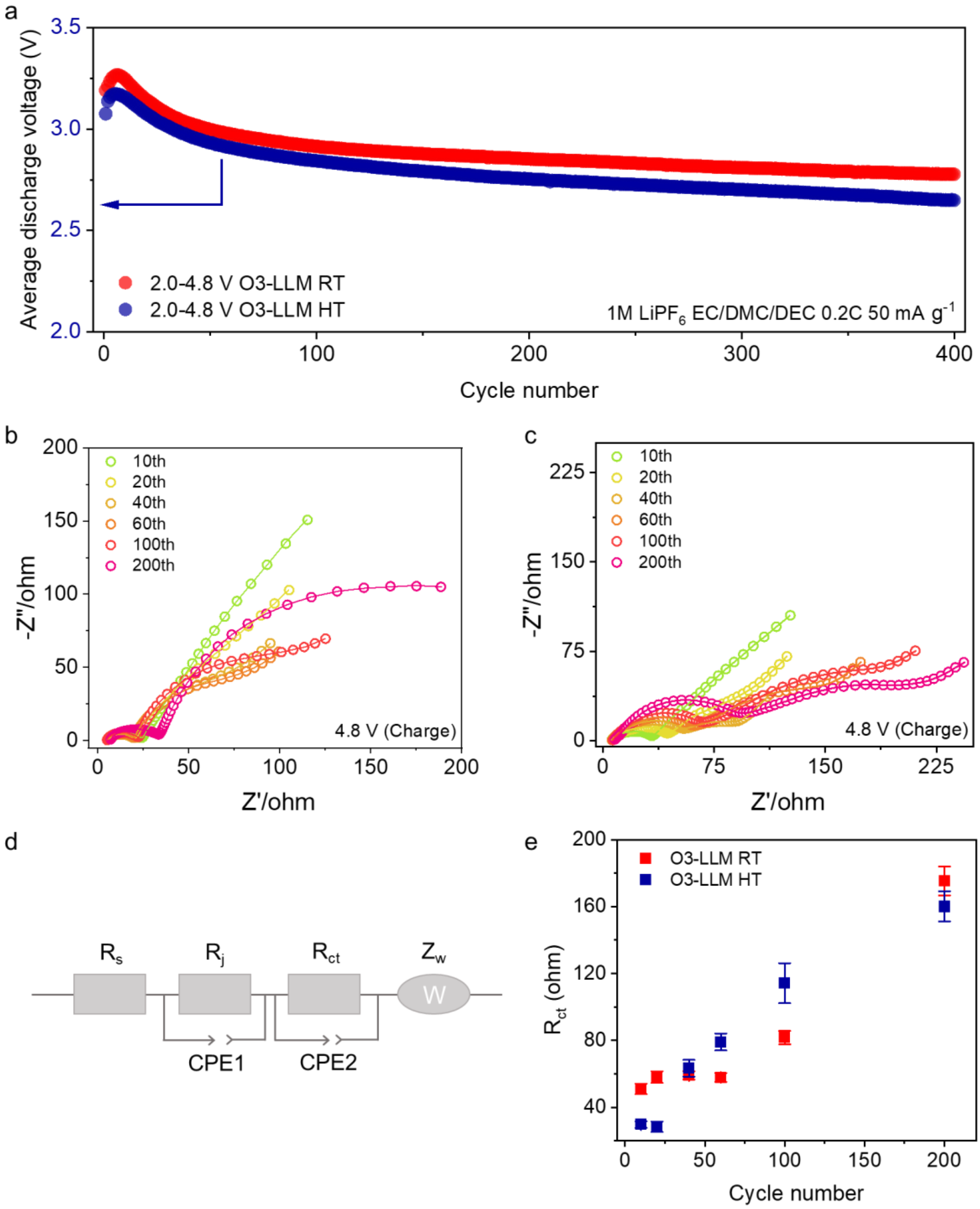

Figure S11. (a) Average discharge voltage at different cycles corresponds to long cycling performance illustrated in Figure 3f. The measured and fitted electrochemical impedance spectroscopy (EIS) of charged (b) O3-LLM RT and (c) O3-LLM HT cathodes at different cycles. (d) The equivalent circuit of EIS fitting for the O3-LLM electrode. (e) The fitted R_{ct} (Charge transfer impedance) value at different cycles

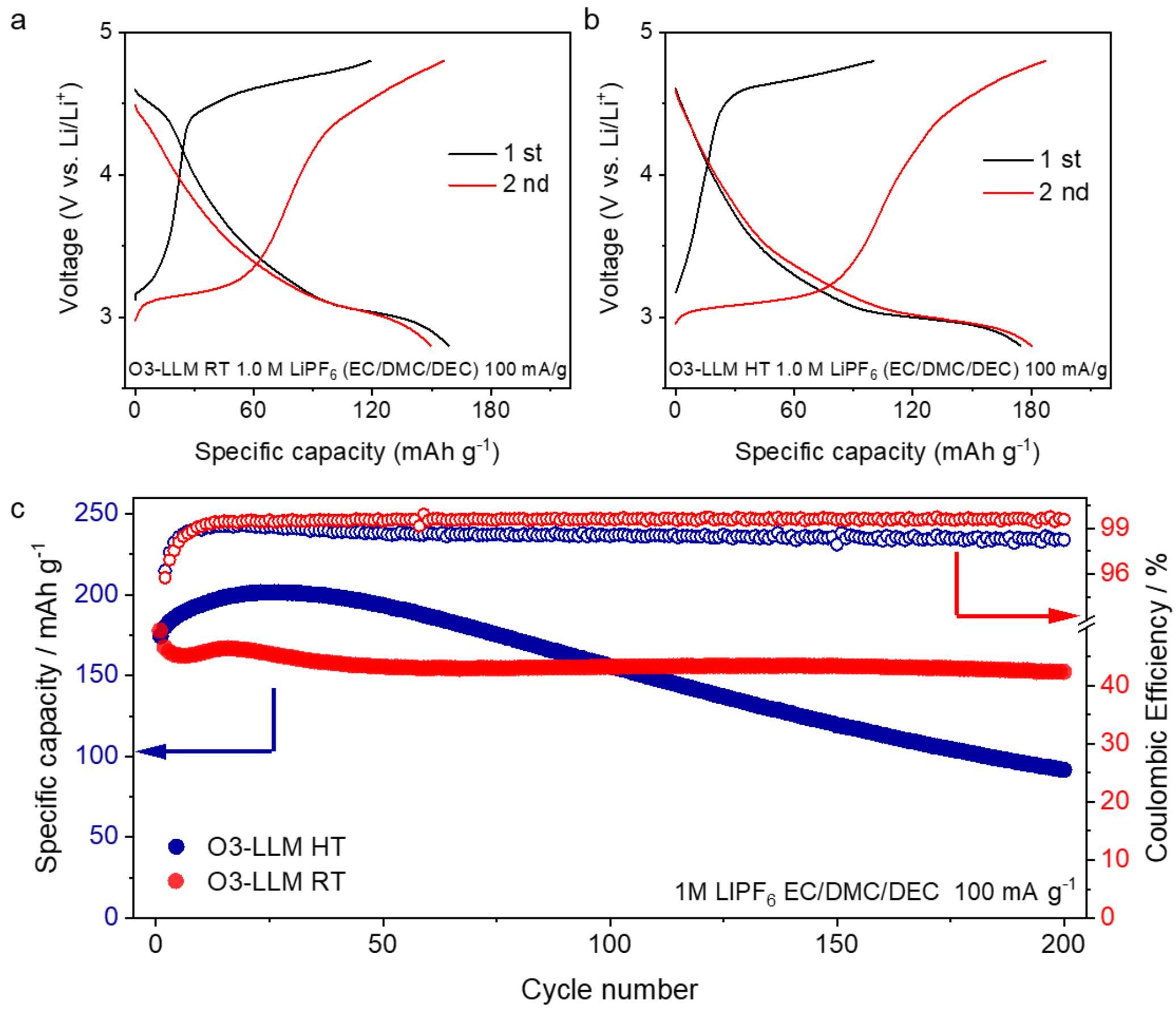

Figure S12. (a) Charge/discharge curves for O3-LLM RT and (b) HT electrode between 2.8 and 4.8 V at 100 mA g^{-1}. (c) long cycling performance of O3-LLM RT and HT within the 2.8 – 4.8 V voltage range.

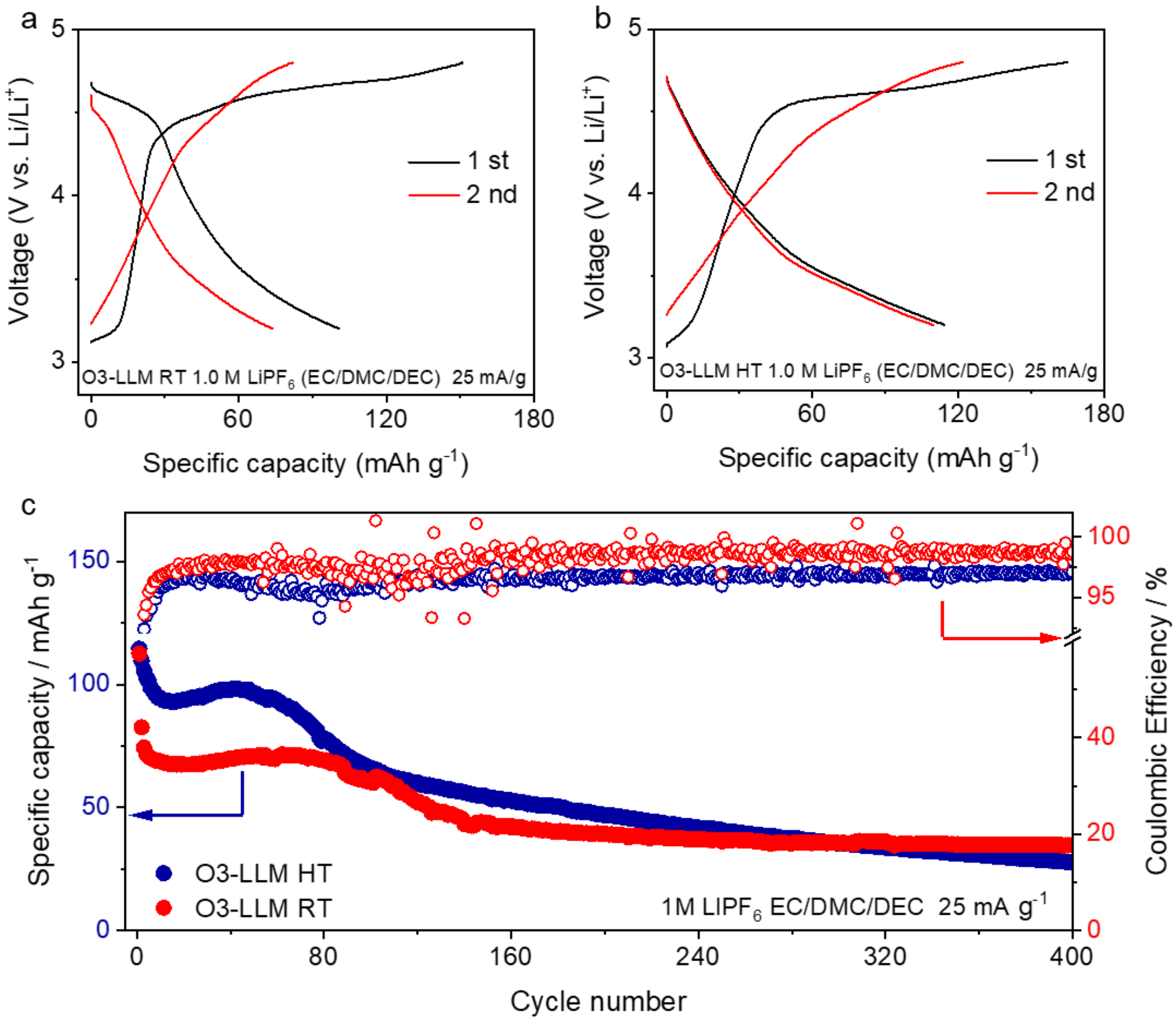

Figure S13. (a) Charge/discharge curves for O3-LLM RT and (b) HT electrode between 3.2 and 4.8 V at 25 mA g^{-1}. (c) long cycling performance of O3-LLM RT and HT within the voltage range of 3.2 – 4.8 V. 1 **M** $LiPF_6$ in EC/DMC/DEC (1:1:1) was used as the electrolyte for battery assembly.

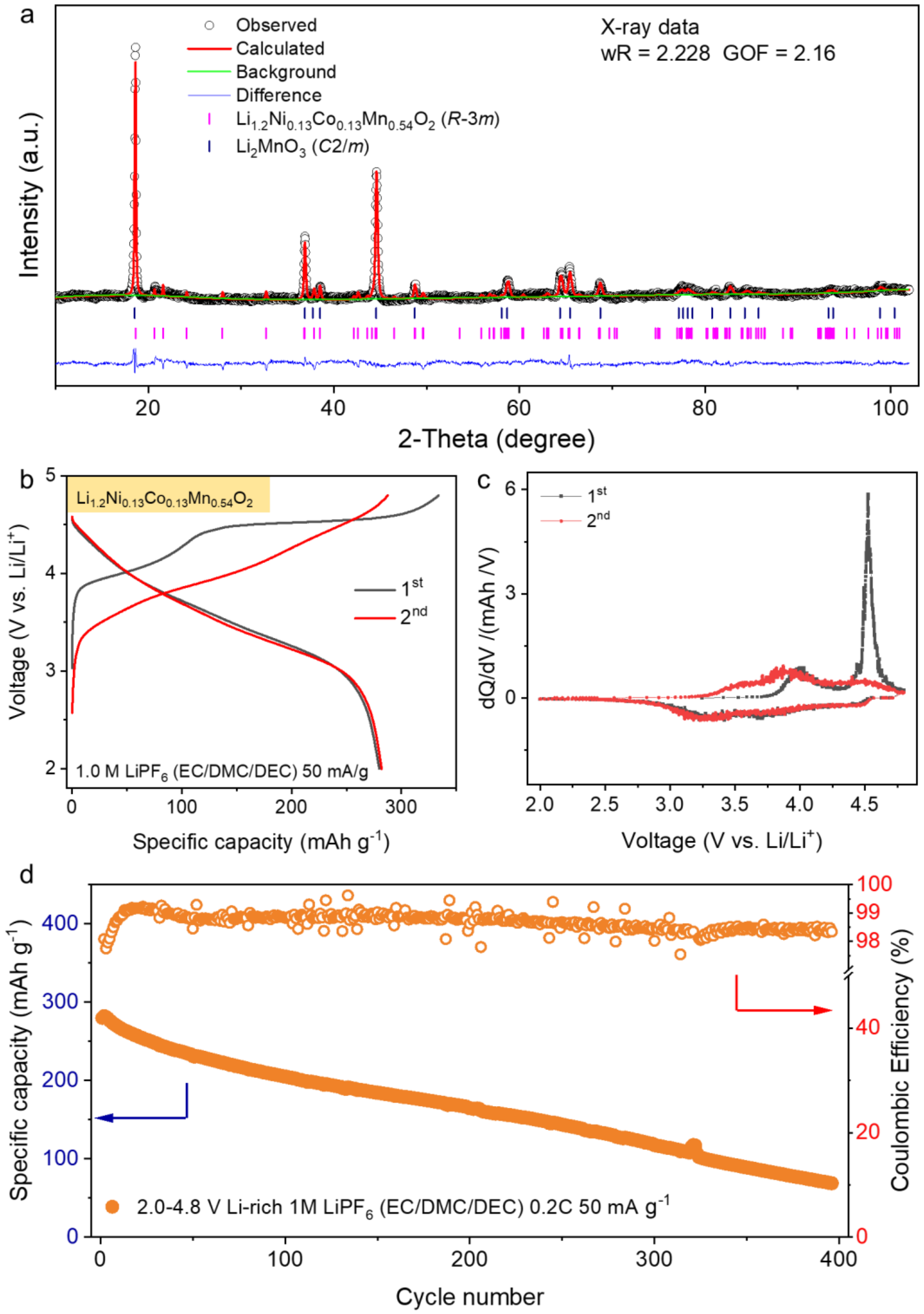

Figure S14. (a) Experimental and Rietveld refinement XRD profiles of $Li_{1.2}Ni_{0.13}Co_{0.13}Mn_{0.54}O_2$ (O3-LNCM) cathode. The refined crystallographic parameters are provided in Table S6. (b) Charge–discharge curves at 50 mA/g between 2.0 and 4.8 V vs metallic Li. (c) The corresponding dQ/dV curves and (d) cycling stability of Li-rich NMC. 1 **M** $LiPF_6$ in EC/DMC/DEC (1:1:1) was used for battery assembly.

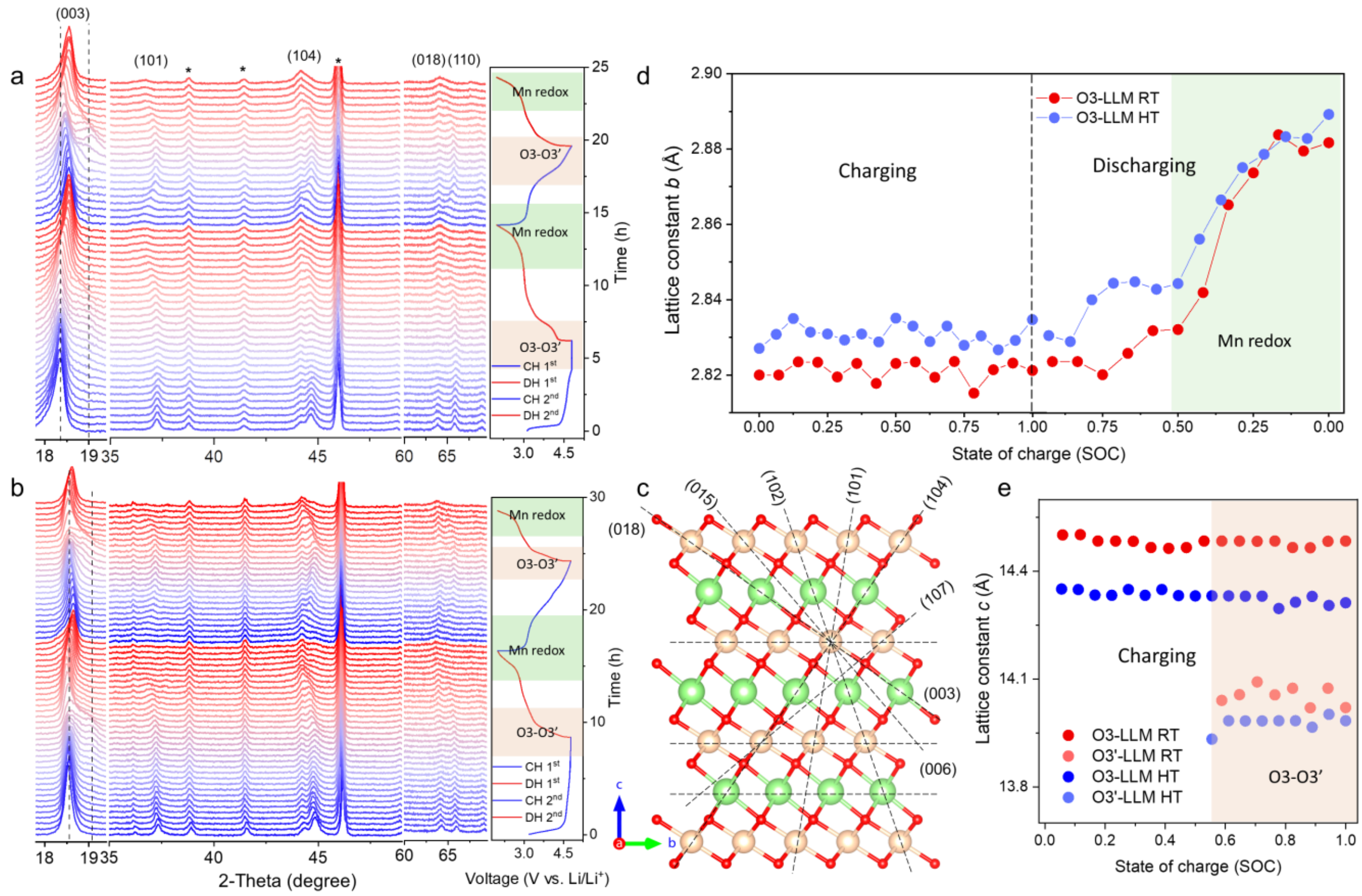

Figure S15. *In situ* X-ray characterizations of O3-LLM RT and HT cathodes. The contour plots of the *in-situ* XRD patterns for (a) O3-LLM RT and (b) O3-LLM HT electrodes during the first two cycles, where the XRD peaks of Be are marked with "*". The high/low-voltage regions are marked with red and green shaded areas, respectively. (c) Schematic representation of the lattice planes of O3-type oxides. The evolution of lattice constant (d) ***b*** and (e) ***c*** during the first charging-discharging process, which were calculated by the position of the plane of (003) and (104). The state of charges (SOCs) is normalized between 0 (Pristine) and 1 (fully charged).

Supplementary Note 2.

In-situ XRD was performed to analyze the disparity in structural evolution during the first two cycles to uncover further the impact of synthesis temperature on the structural evolution of the O3-LLM synthesized at RT and HT. The two cathodes undergo similar structural transformations, which go through O3-O3'-O3 phases (Figure S15 a, b). During the Li^+ extraction, before the battery is charged to 4.6 V, the diffraction peaks show no significant changes. However, as the charging continues into the voltage plateau, a noticeable decrease in the intensity of the (003) peak can be observed, along with the gradual formation of a shoulder peak at higher angles of ~ 19°, as indicated by the dashed lines. This suggests the formation of the Li-deficient O3' phase with a lower interplanar spacing in the high-

voltage region, which can be ascribed to the cross-layer migration of Li in the TM layer to the AM layer.[24] It will be discussed in ssNMR results. Meanwhile, the (101) and (104) peaks remain unchanged during the first charge but shift to lower angles during discharge. In the second cycle, they shift to a higher angle during charging and to a lower angle during discharging. This is because the O3-LLM RT and HT electrodes are predominantly in the bulk oxidation state of +4 in their original state. When discharging to a voltage plateau of ~3.0 V, a significant amount of tetravalent manganese is reduced to trivalent manganese, causing the Mn-O bonds to lengthen. As shown in Figure S15 c, d, this indicates the expansion of the *a/b* lattice parameters.[25] In subsequent charge-discharge cycles, the redox couple around ~3.2/3.0 V aligns with the systematic shifts of the (101) and (104) peaks. Note that the appearance/disappearance of a Li-deficient hexagonal O3' phase for O3-LLM electrodes is closely related to the anionic redox activity and the migration of Li within the TM layer, which ultimately determines their differences in structural reversibility. Furthermore, the variations in lattice parameters between the two electrodes during the charging process are shown in Figure S15 e. As mentioned in Figure 2, due to synthesis at different temperatures, a small amount of Mn ions is present in the Li layer of the metastable O3-LLM HT sample. This results in the *c* lattice parameter of the RT sample being larger than that of the HT sample. Subsequently, as charging proceeds into the high-voltage region, the *c* lattice parameter of the RT sample remains larger than that of the HT sample. Aside from this, the overall trend in their changes remains broadly consistent.

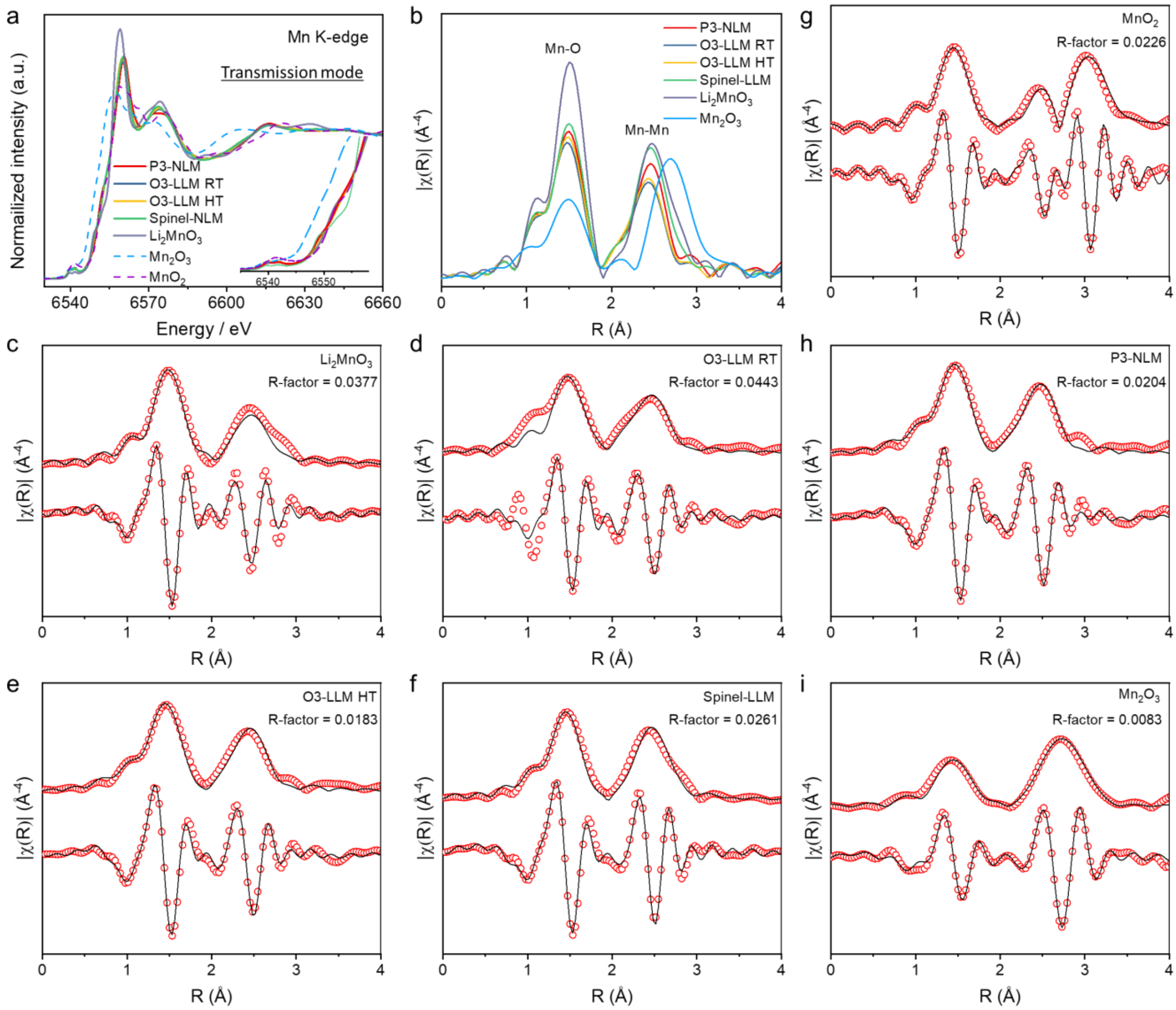

Figure S16. (a) Normalized Mn K-edge XANES spectra on powder pellet samples of P3-NLM, O3-LLM (RT and HT), Spinel-LLM, and reference compounds including Li_2MnO_3, Mn_2O_3, and MnO_2, and (b) the corresponding K-edge EXAFS of these layered LLM oxides. Mn K-edge EXAFS data fits for (c) layered Li_2MnO_3, (d) O3-LLM RT, (e) O3-LLM HT, (f) Spinel-LLM, (g) MnO_2, (h) P3-NLM, and (i) Mn_2O_3, shown as the magnitude and real part of the Fourier-transformed R-space spectra (offset vertically for clarity). The corresponding refined structural parameters, including bond length (R), coordination number (CN), and Debye–Waller factor (DW), are summarized in Table S8.

Supplementary Note 3.

After ion exchange, the Mn K-edge XANES spectra of O3-LLM RT, O3-LLM HT, and spinel-LLM show that the white line positions closely align with those of tetravalent references (Li_2MnO_3, P3-NLM, and MnO_2), exhibiting a shift of approximately ~3 eV relative to the trivalent Mn_2O_3 reference at the half-maximum height. This indicates that Mn in the bulk of the ion-exchanged products predominantly adopts the +4 oxidation state, irrespective of the synthesis conditions and temperatures. EXAFS fitting further confirms this observation: the Mn-O bond lengths in O3-LLM RT, O3-LLM HT, and spinel-LLM remain around 1.88 Å, showing only minor variations, and are significantly shorter than those in Mn_2O_3 (~1.94–2.2 Å).

Interestingly, the EXAFS analysis also reveals a gradual decrease in the Mn-O coordination number from O3-LLM RT to O3-LLM HT and further to spinel-LLM, suggesting a subtle disruption of the MnO_6 octahedral environment at higher synthesis temperatures. Specifically, the coordination number decreases from ~4.5 in O3-LLM RT/HT to ~4.37 in spinel-LLM, accompanied by a slight increase in the Debye–Waller factors (σ^2), indicative of enhanced local disorder around Mn. These local structural variations are consistent with long-range structural information from variable-temperature and refined XRD: the intensity ratio of the (003)/(104) peaks decrease from 2.71 to 2.56, and the (018)/(110) peaks begin to overlap, signaling incipient layer-to-spinel transformations and partial Mn migration. Complementary solid-state ^{7}Li NMR measurements further show minor changes in the Li local environment correlated with increased Mn mobility.

Taken together, these observations quantitatively demonstrate that higher synthesis temperatures induce partial Mn migration from the transition-metal layers, leading to minor distortions of the MnO_6 octahedra, while the tetravalent Mn oxidation state in the bulk is largely preserved.

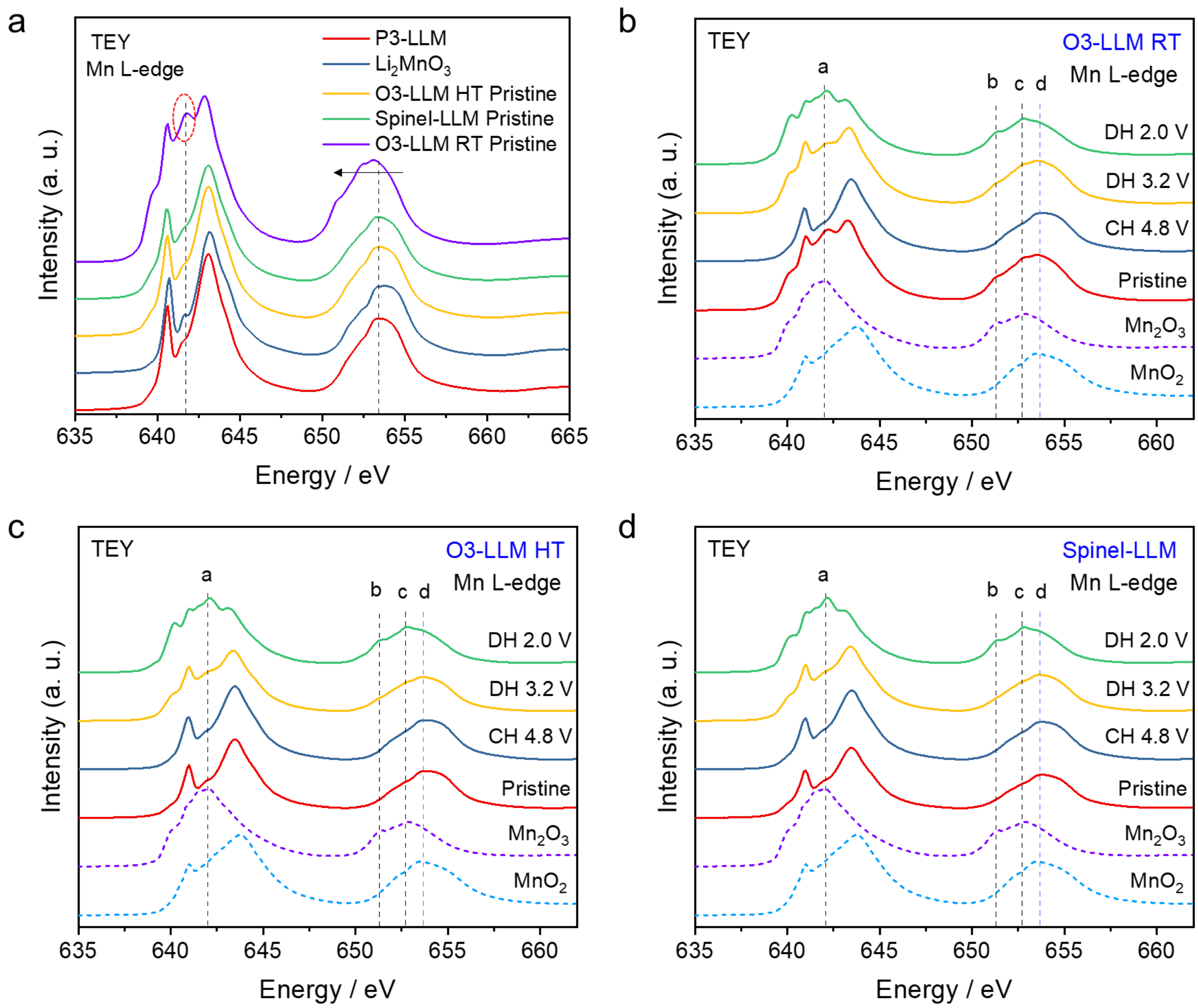

Figure S17. (a) Mn $L_{2,3}$-edge XAS spectra (TEY mode) of pristine O3-LLM prepared at different synthesis temperatures, benchmarked against reference compounds $Li_2Mn^{IV}O_3$ and P3-NLM. Mn $L_{2,3}$-edge XAS collected from TEY mode of (b) O3-LLM RT, (c) O3-LLM HT and (d) Spinel-LLM at different states of charge. Absorption events characteristic of Mn oxidation states of 3+ and 4+ have been provided as a guide to the eye. The Mn $L_{2,3}$-edge XAS spectra unambiguously indicate that the pristine O3-LLM RT sample adopts a mixed Mn^{3+}/Mn^{4+} valence configuration.

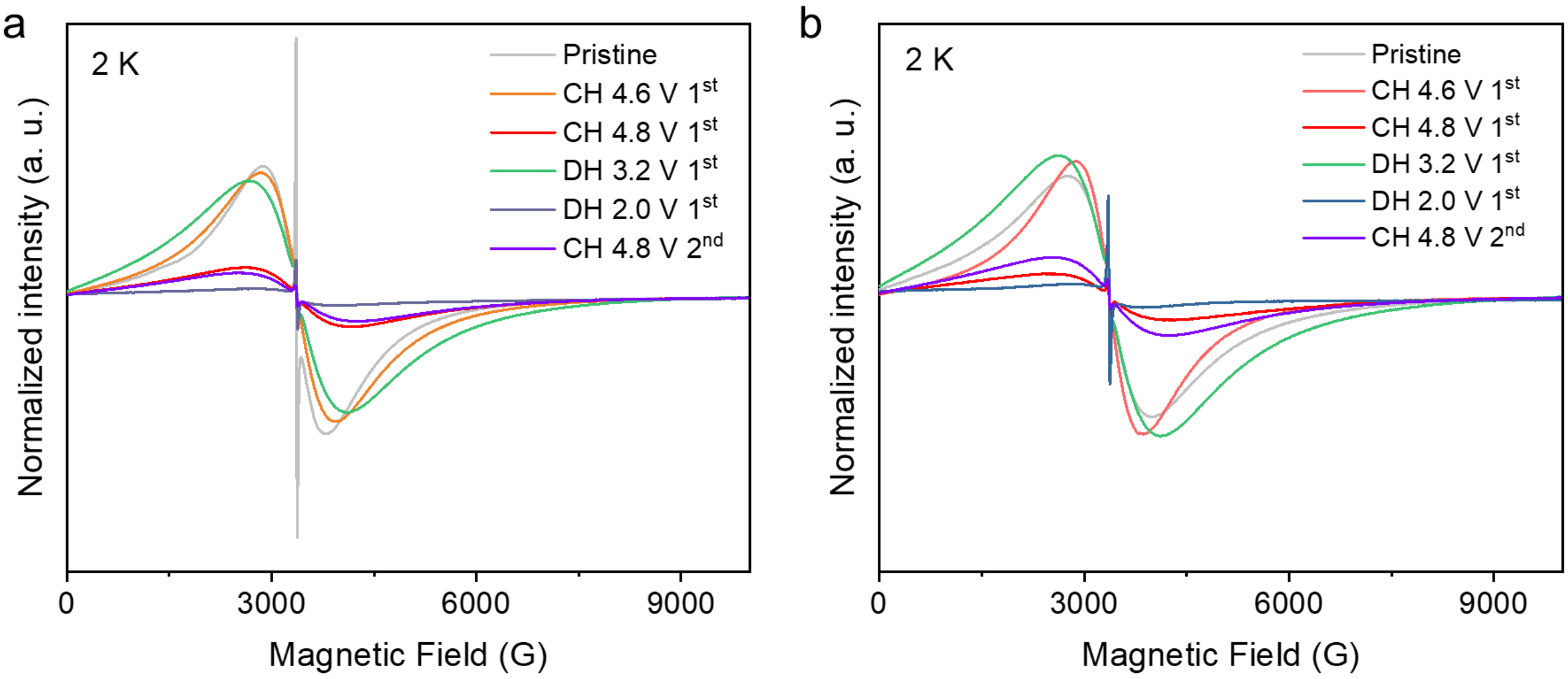

Figure S18. (a) Ex-situ electron paramagnetic resonance (EPR) X-band spectra under 2 K of (c) O3-LLM RT and (d) O3-LLM HT electrodes at different charge/discharge states.

Supplementary Note 4.

To further investigate the evolution of Mn valence states, ex-situ electron paramagnetic resonance (EPR) X-band spectra were collected for O3-LLM RT and O3-LLM HT electrodes at various charge/discharge states. In paramagnetically concentrated systems, EPR is capable of detecting transition metal clusters *via* dipole–dipole and magnetic exchange interactions[26]. Owing to its high sensitivity to unpaired electron spins, EPR is particularly effective for detecting Mn^{4+} ions (S = 3/2), which exhibit a broad resonance signal centered around g ≈ 2.0. In contrast, Mn^{3+} ions are typically EPR-silent due to their integer spin state[27]. As shown in Figure S18, both O3-LLM RT and O3-LLM HT display broad EPR signals in their pristine states, which are attributed to Mn^{4+} species in octahedral coordination. Upon discharge to 2.0 V, the EPR signals are markedly suppressed or nearly vanish, likely reflecting the reduction of Mn^{4+} to EPR-silent Mn^{3+}. This observation aligns well with the Mn K-edge XAS results, further supporting the reversible Mn^{4+}/Mn^{3+} redox activity during cycling.

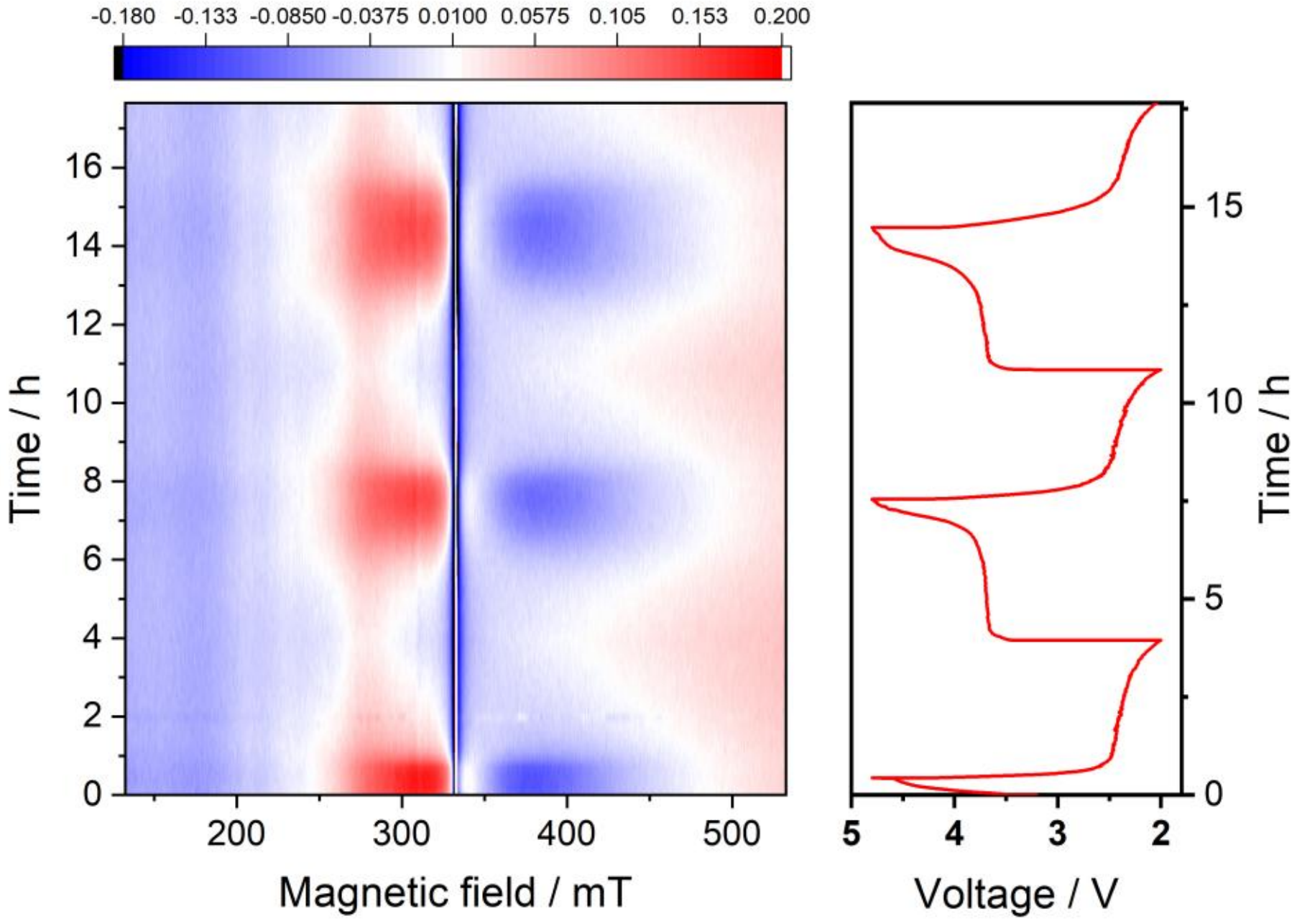

Figure S19. Operando EPR spectra of the O3-LLM RT||Li cell for the first three cycles between 2.0-4.8 V. Because the sealing and applied pressure of the battery cannot be adequately ensured during operando EPR measurement, the lab-made electrochemical cell that was used for the experiment has a much higher resistance than the coin cells used for galvanostatic cells. Therefore, the polarization was very high. However, the redox couple of $Mn^{3+}/^{4+}$ upon cycling was clearly captured as depicted by signal changes within a voltage range of 2-3 V.

Supplementary Note 5.

Since Mn^{3+} is silent in X-band perpendicular-mode EPR spectra[28], operando EPR can monitor changes in the Mn^{4+}/Mn^{3+} redox couple in Li-Mn-O systems. However, due to the limited size of the EPR cavity, investigating battery devices with EPR faces the challenge of skin effects, which restrict the penetration of microwave (MW) radiation into metallic solids to a surface layer with a depth (δ) on the order of hundreds of nanometers to micrometers at commonly used X-band frequencies (~ 9.5 GHz).[26] As a result, it is difficult to achieve proper battery sealing using PET/EVA laminating films and sufficient pressure to ensure minimal cell polarization. Nevertheless, operando EPR data still detected the disappearance (during discharge) and reappearance (during charge) of Mn signals in the low voltage range in Figure S19. This suggests the presence of a reversible Mn^{4+}/Mn^{3+} redox process in the O3-LLM RT electrode.

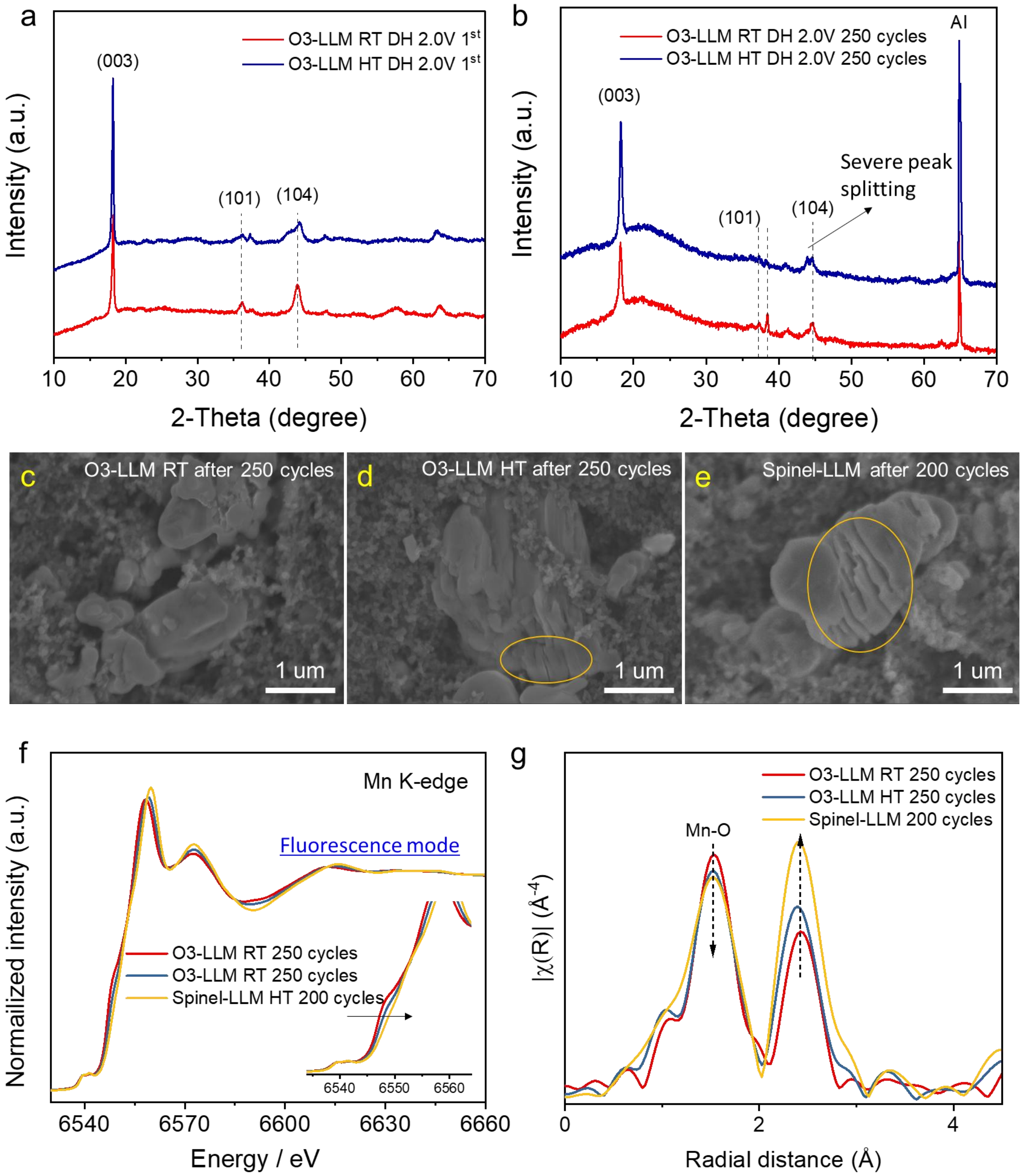

Figure S20. XRD patterns of O3-LLM RT and O3-LLM HT electrodes (a) after the first discharge to 2.0 V, (b) long cycling test. SEM images of (c) O3-LLM RT, (d) O3-LLM HT and (e) Spinel-LLM electrodes after extended cycling. (f) Normalized Mn K-edge XANES spectra on cycled O3-LLM (RT and HT), Spinel-LLM electrodes, (g) the corresponding K-edge EXAFS spectra.

Supplementary Note 6.

To further probe the structural evolution upon cycling, *ex-situ* XRD measurements were performed on electrodes discharged to 2.0 V. As shown in Figure S20 a, b, the O3-LLM HT sample exhibits a clear splitting of the (104) reflection, indicative of phase inhomogeneity and lattice distortion. This observation aligns with ^{7}Li NMR results, which reveal that Li^{+} ions in the O3-LLM HT cathode do not fully migrate into the transition metal layers upon discharge, leaving behind transition metal vacancies. Additionally, Raman spectroscopy also reveals a persistent A'_{1g} mode at ~664 cm^{-1} in O3-LLM HT, characteristic of TM_{Li}–V_{TM} (V: vacancy) anti-site defects, further confirming Mn migration–induced structural disorder. After 250 cycles, SEM imaging reveals the development of microcracks in the O3-LLM HT electrode, while more pronounced intergranular cracking is observed in the Spinel-LLM electrode. In contrast, the O3-LLM RT electrode retains superior morphological integrity, with minimal signs of mechanical degradation (Figure S20 c, d, e).

Mn K-edge XAS spectra collected on discharged electrodes after extended cycling (Figure S20 f, g) show a systematic rightward shift in the absorption edge from O3-LLM RT to HT and further to Spinel-LLM. This trend is contrary to the expected reduction toward Mn^{3+} and instead suggests a progressive suppression of Mn reduction during discharge. Such behavior implies a loss of redox-active Mn and a corresponding decrease in capacity contribution. The Mn K-edge EXAFS data in R-space and compared the relative intensities of the Mn-O and Mn-TM coordination peaks across the three samples. A systematic decrease in the Mn-O/Mn-TM intensity ratio was observed with increasing structural degradation. This trend reflects a progressive disruption of the Mn-O coordination environment, likely driven by cumulative oxygen loss and transition metal migration upon repeated cycling, particularly in O3-LLM HT and Spinel-LLM.

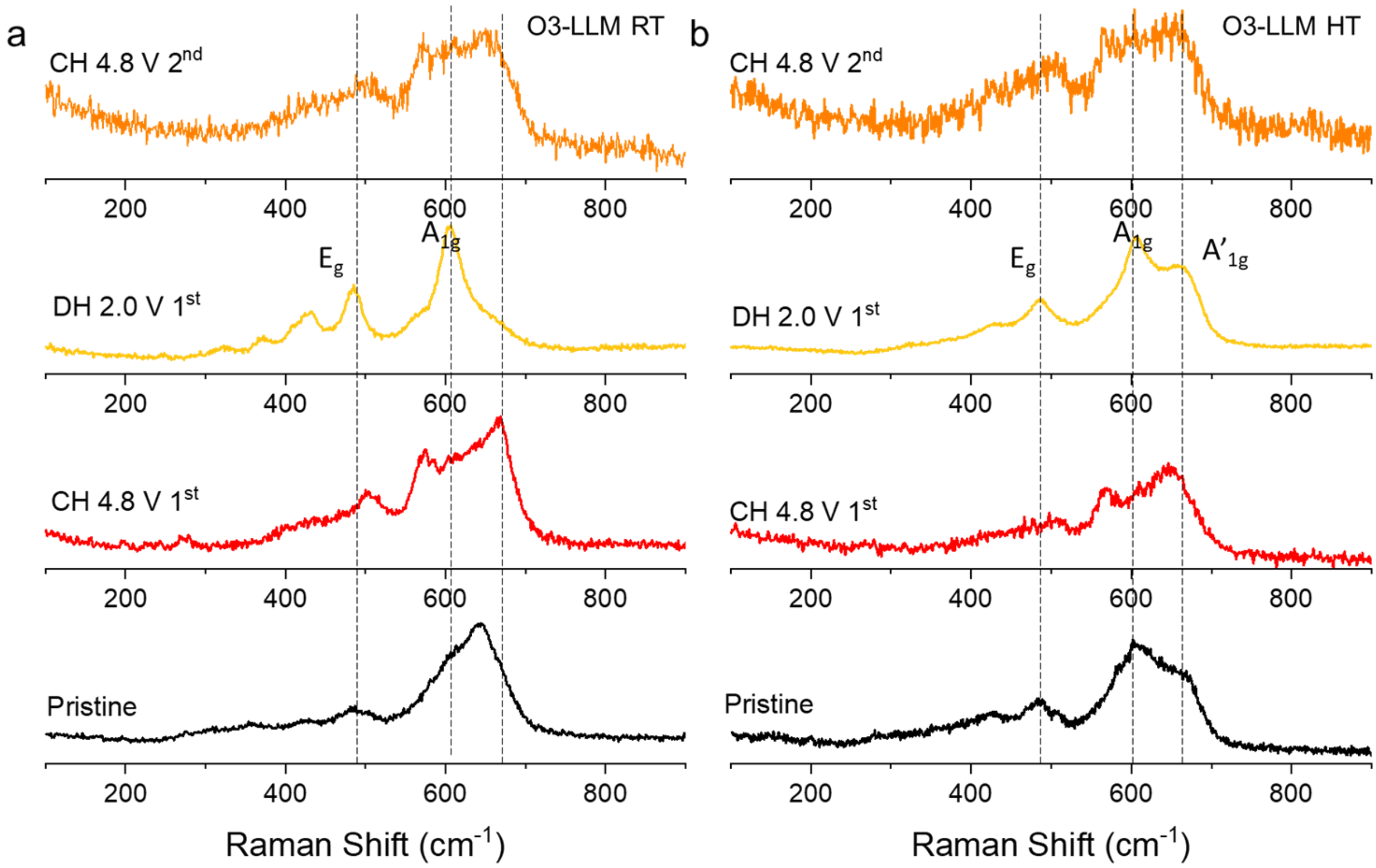

Figure S21. Different cation migration behaviors of the two materials. Raman spectra for (a) O3-LLM RT and (b) O3-LLM HT electrodes at different states of charge.

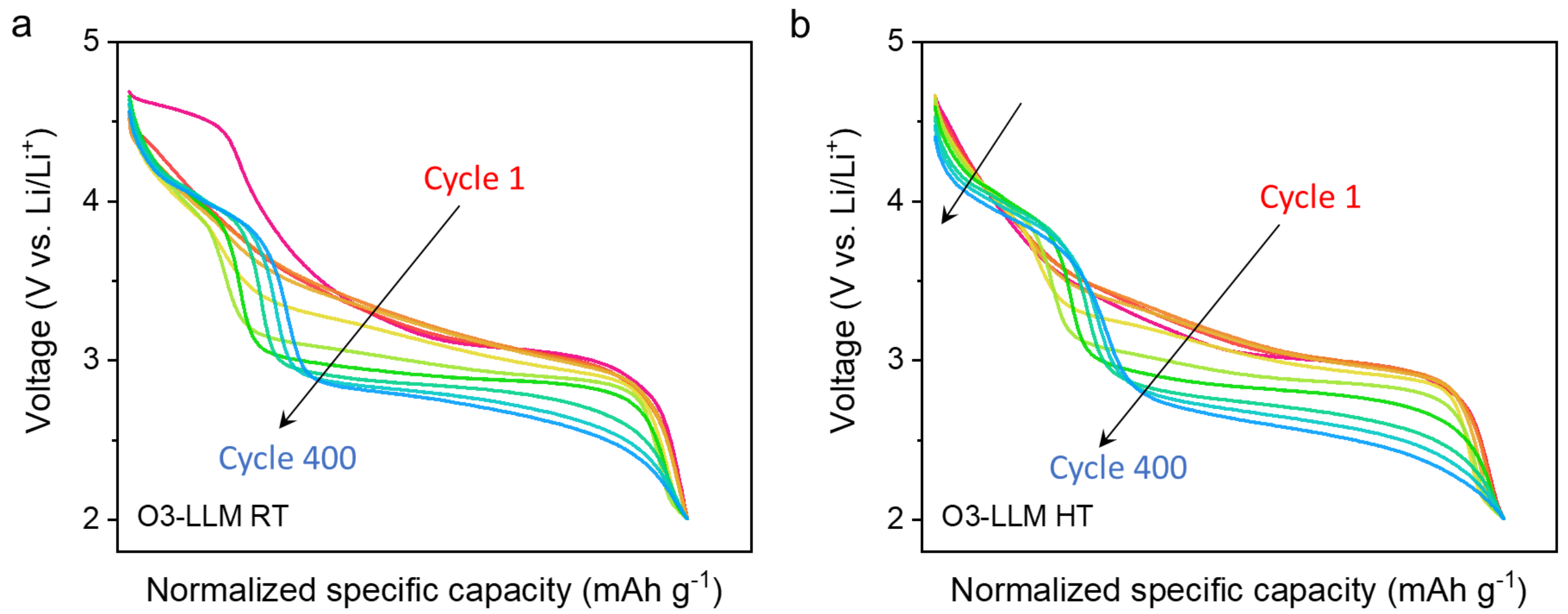

Figure S22. Normalized discharge curves of (a) O3-LLM RT and (b) O3-LLM HT electrode for 400 cycles, which indicates that the O3-LLM RT electrode has better structural reversibility at high voltage region.

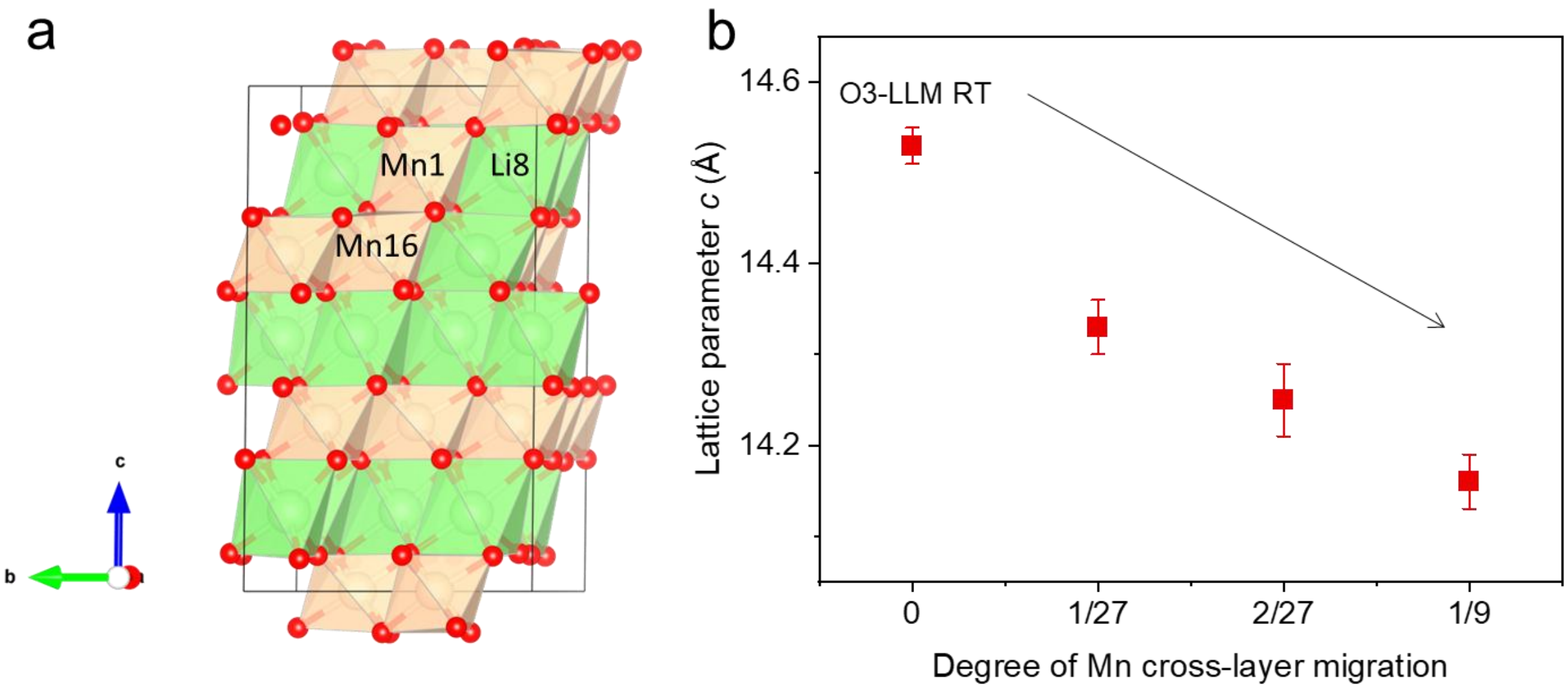

l(Mn16-O26) = 1.87788(0) Å	l(Mn1-O37) = 1.96649(0) Å	l(Li8-O10) = 2.12233(0) Å
l(Mn16-O20) = 1.92982(0) Å	l(Mn1-O11) = 1.99067(0) Å	l(Li8-O39) = 2.01978(0) Å
l(Mn16-O11) = 1.93855(0) Å	l(Mn1-O38) = 2.01684(0) Å	l(Li8-O37) = 2.23502(0) Å
l(Mn16-O12) = 1.91035(0) Å	l(Mn1-O14) = 1.92852(0) Å	l(Li8-O13) = 2.01873(0) Å
l(Mn16-O21) = 1.87881(0) Å	l(Mn1-O15) = 1.96305(0) Å	l(Li8-O40) = 2.06388(0) Å
l(Mn16-O15) = 1.99386(0) Å	l(Mn1-O41) = 1.97607(0) Å	l(Li8-O14) = 2.12076(0) Å
--------------------------	---------------------------------------	---------------------------------------
Average bond length = 1.9215 Å	Average bond length = 1.9736 Å	Average bond length = 2.0968 Å
Polyhedral volume = 9.3701 Å^3	Polyhedral volume = 10.0311 Å^3	Polyhedral volume = 12.1399 Å^3

Figure S23. (a) The DFT-relaxed crystal structure of the defective O3-LLM where the Mn ion migrates to the Li layer with a vacancy left (O3-LLM HT). (b) Dependence of the lattice parameter c on the degree of Mn migration across layers.

Supplementary Note 7.

To simulate the O3-LLM RT (perfect layered structure without Mn migration) and O3-LLM HT (Mn ions migrate to the Li layer leaving vacancies in the TM layer) models, structural enumeration and first-principles calculations were performed. For the O3-LLM RT model, a 3×3×1 supercell corresponding to the composition $Li_{6/9}[Li_{2/9}Mn_{7/9}]O_2$ was generated. Using the pymatgen package, 20 symmetrically distinct configurations were enumerated and screened based on their Ewald energies. The configuration with the lowest total energy was selected as the thermodynamically stable structure and used for subsequent calculations. For the O3-LLM HT model with same composition of $Li_{6/9}[Li_{2/9}Mn_{7/9}]O_2$, Mn migration was explicitly introduced. Three levels of Mn migration (1/27, 2/27, and 3/27 of the total TM sites) were considered within the same 3×3×1 supercell, corresponding to the migration of one, two, or three Mn ions from the TM layer into the Li layer, with the original TM sites left as vacancies. For each Mn migration concentration, 20 possible configurations were generated, and all structures were optimized. The optimized lattice parameter c was then averaged over the ensemble to evaluate the influence of Mn migration content on the interlayer spacing.

To further understand the evolution of electronic structure, crystal orbital Hamilton population (COHP) calculations were performed on the O3-LLM RT and HT models under highly charged states. In these states, Li ions in the TM layer are prone to migrate into the Li layer, which is consistent with the observations from ^{7}Li NMR spectroscopy in Figure 5. For the O3-LLM RT model, which possesses a perfect layered structure, the Li migration from the TM layer leaves behind well-ordered vacancies. These vacancies are periodically arranged due to the intrinsic symmetry of the structure, leading to relatively uniform local environments. In contrast, the O3-LLM HT model inherently contains Mn vacancies in the TM layer as a result of Mn migration into the Li layer. Under highly delithiated

conditions, additional Li migration from the TM layer creates new vacancies that spatially interact with the pre-existing Mn-induced vacancies. This leads to the formation of vacancy clusters or defect complexes. Such clustering effects significantly modify the local electronic environment, which is reflected in the COHP results as changes in O–O bonding interactions. These defect-induced variations highlight the distinct electronic and structural responses of O3-LLM RT and HT models under deep charging conditions.

From the detailed structural analysis, the local polyhedral environments can be described as: (i) Mn atoms remaining in the TM layer (e.g., Mn_{17}), (ii) the migrated Mn ion in the Li layer (e.g., Mn_1), and (iii) Li atoms adjacent to the migrated Mn in the Li layer (e.g., Li_9). Although the polyhedral volume of the migrated Mn ion slightly decreases after migration, it is significantly smaller compared with the neighboring Li polyhedra. This local contraction reduces the interlayer spacing. Furthermore, statistical analysis across all configurations confirms that as the degree of Mn migration increases, the lattice parameter c systematically decreases. This computational trend is in excellent agreement with experimental observations from VT-XRD and *in-situ* XRD measurements.

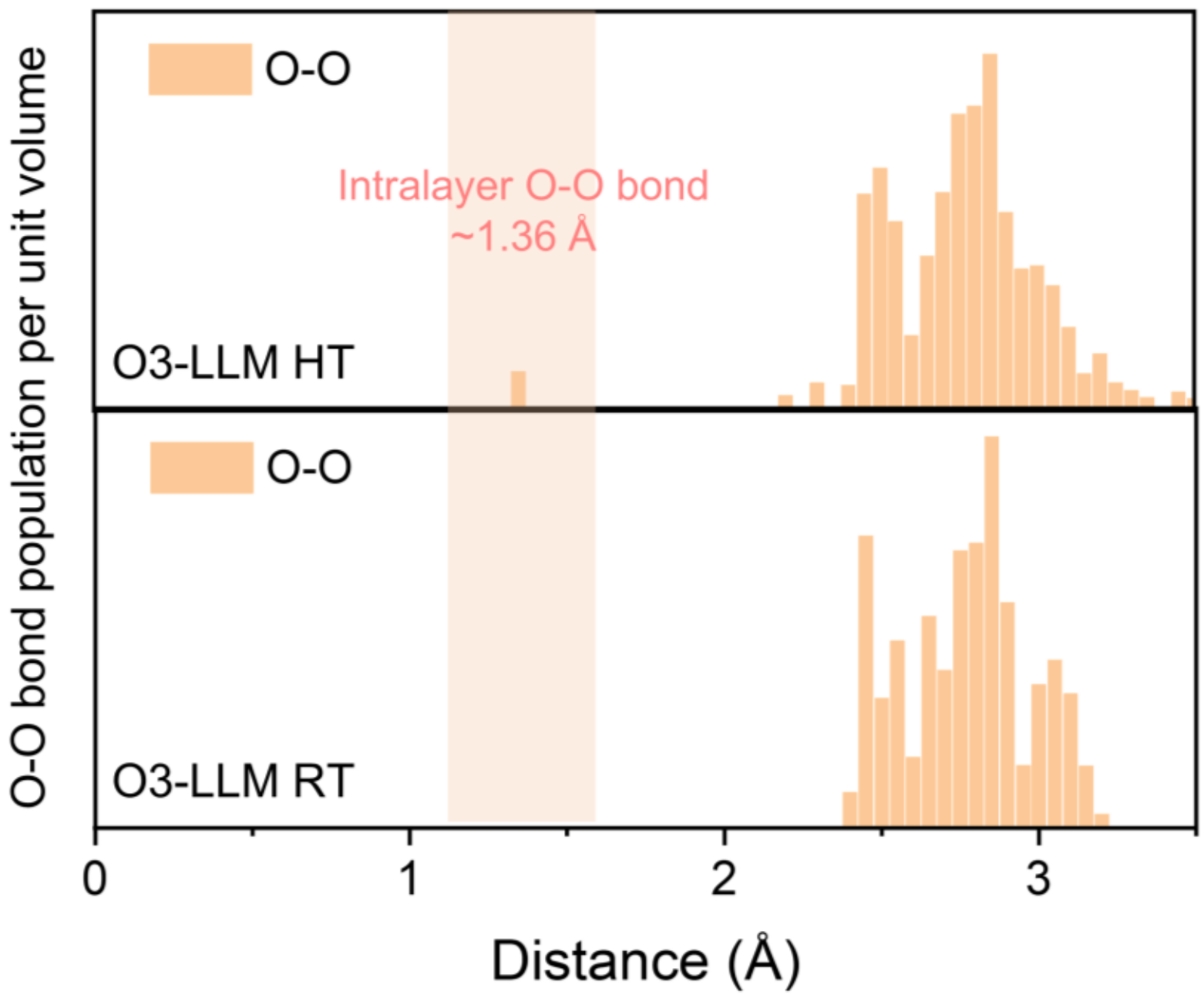

Figure S24. The counted O−O populations per unit volume in the fully delithiated O3-LLM.

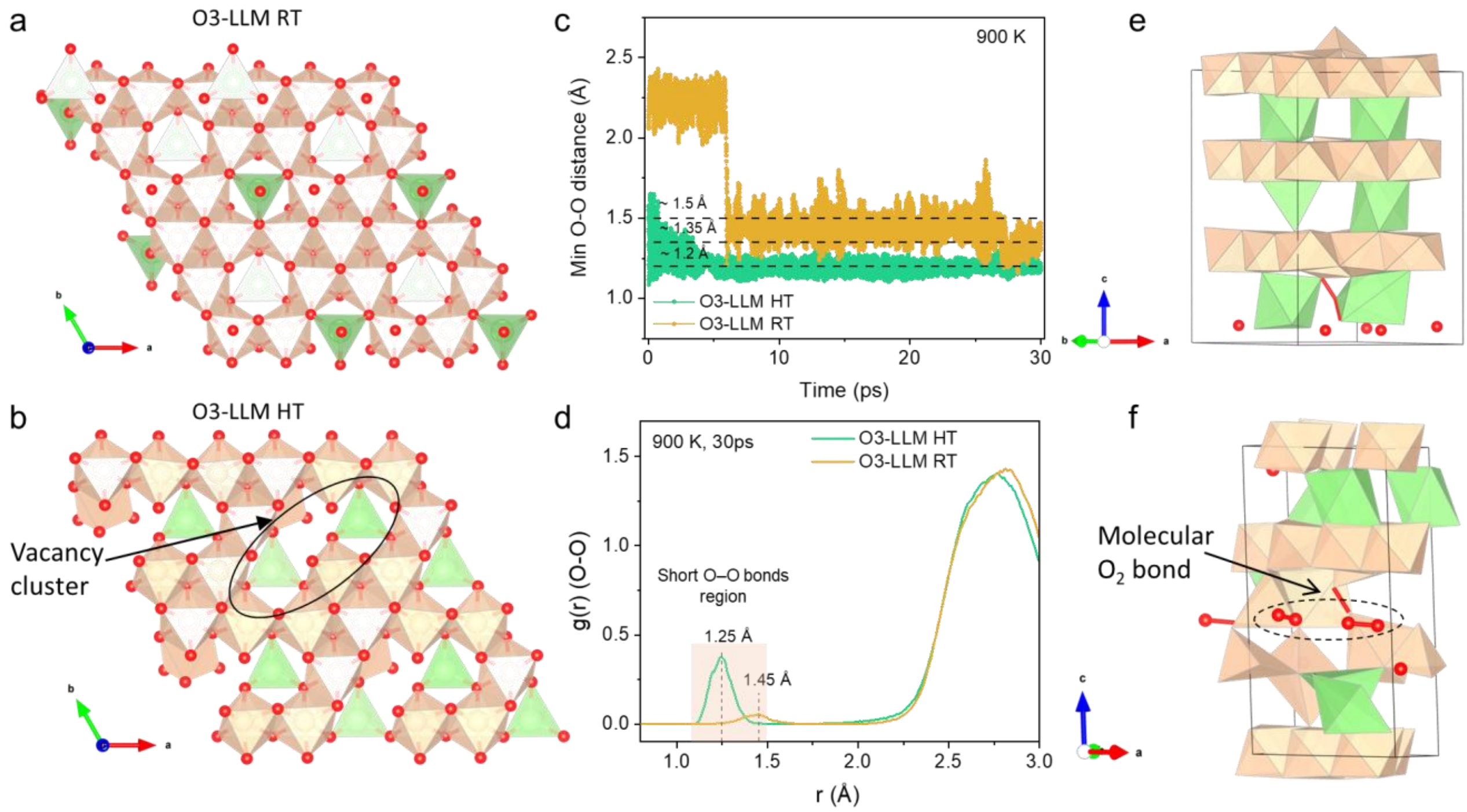

Figure S25. Atomic configurations and O-O bonding evolution of highly delithiated $Li_{2/9}Li_{7/9}O_2$ (O3-LLM). (a) TM layer of O3-LLM RT and (b) HT structures with one Mn migration event (1/27), corresponding to a single Mn atom moving into the Li layer. (c) Time evolution of the shortest O-O bond length during AIMD simulations of highly delithiated O3-LLM RT and HT. (d) Radial distribution function (RDF) of O-O species for O3-LLM RT and HT. Snapshots of the atomic structures after 30 ps for (e) O3-LLM RT and (f) HT, highlighting possible O-O configurations. Orange, green, and red represent Li^+, Mn, and lattice O ions, respectively. Solid ellipses indicate the formation of vacancy clusters, while dashed ellipses highlight molecular oxygen species. The source code for statistical analyses of the RDF and shortest O-O bond length is provided.

Supplementary Note 8.

As shown in the Figure S25 a, b, in the highly delithiated state, when a single Mn ion in the transition-metal layer undergoes interlayer migration, the Li ions in the transition-metal layer also exhibit interlayer migration at high voltage, consistent with observations from ^{7}Li NMR. At this stage, vacancy clusters appear in O3-LLM HT, whereas the vacancies in O3-LLM RT remain ordered. By heating the metastable O3-LLM RT and HT structures to 900 K and holding them at this temperature for 30 *ps* (see Methods), the evolution of the shortest O-O bonds in the two structures can be clearly distinguished. Due to the presence of vacancy clusters in the transition-metal layer of O3-LLM HT, local electronic and atomic arrangements are highly disordered, and the shortest O-O bond fluctuates between 1.2-1.5 Å. In contrast, in O3-LLM RT, the shortest O-O bond remains around 2.2 Å during the first 5 ps of simulation and only decreases to ~1.45 Å under thermal excitation, remaining at ~1.4 Å—significantly longer than the 1.25 Å observed in O3-LLM HT (Figure S25 c). Further statistical analysis of O-O coordination over the full 30 ps simulation yields the radial distribution function (RDF) of O···O species (Figure S25 d), highlighting how differences in local structure lead to distinct kinetic pathways for O-O evolution. Compared with O3-LLM RT, the O3-LLM HT structure exhibits unstable molecular oxygen species with a bond length of 1.25 Å (Figure S25 f). The electronic structure of these oxygen species has been revealed by the COHP calculations in Figure 6. Importantly, such molecular oxygen species can inevitably contribute to oxygen loss during long-term cycling, consistent with the observed decrease in the pre-edge intensity of the O-K edge in soft X-ray measurements and EXAFS results from hard X-rays.

Supplementary Tables

Tab. S1 Rietveld refinement results of XRD data for P3-NLM material.

Space group: *P*1; a = 9.86(4); b = 7.54(2); c = 16.86(7); alpha = 83.3(2); beta = 82.3(1); gamma = 70.95(7); volume = 1171.(3) wR = 2.817; GOF = 2.26						
label	element	multiplicity	x	y	z	fraction
Na1	Na	1	0.03915	0.019146	0.839532	1
Na2	Na	1	0.510525	0.029918	0.839449	1
Na3	Na	1	0.793621	0.647061	0.827265	1
Na4	Na	1	0.321756	0.635797	0.827467	1
Na5	Na	1	0.116465	0.409012	0.836291	1
Na6	Na	1	0.71526	0.257287	0.831017	1
Na7	Na	1	0.872874	0.687835	0.506596	1
Na8	Na	1	0.342989	0.692425	0.50489	1
Na9	Na	1	0.625025	0.313248	0.494029	1
Na10	Na	1	0.154904	0.305016	0.494464	1
Na11	Na	1	0.950722	0.076598	0.501613	1
Na12	Na	1	0.548691	0.919814	0.498196	1
Na13	Na	1	0.676865	0.362736	0.172068	1
Na14	Na	1	0.205816	0.353429	0.172646	1
Na15	Na	1	0.959283	0.977922	0.159847	1
Na16	Na	1	0.488092	0.972789	0.161292	1
Na17	Na	1	0.283083	0.745386	0.168099	1
Na18	Na	1	0.881708	0.589741	0.164425	1
Mn1	Mn	1	0.299011	0.804761	-0.0029	1
Mn2	Mn	1	0.798236	0.807665	-0.00279	1
Mn3	Mn	1	0.201268	0.191243	0.002909	1
Mn4	Mn	1	0.700761	0.194279	0.002356	1
Mn5	Mn	1	0.402657	0.391902	0.000463	1
Mn6	Mn	1	0.900964	0.393125	0.001113	1
Mn7	Mn	1	0.235022	0.059499	0.667809	1
Mn8	Mn	1	0.73332	0.061201	0.667733	1
Mn9	Mn	1	0.130939	0.472601	0.664714	1
Mn10	Mn	1	0.630703	0.475431	0.664466	1
Mn11	Mn	1	0.03368	0.859274	0.669581	1
Mn12	Mn	1	0.53299	0.862011	0.669894	1
Mn13	Mn	1	0.964957	0.139713	0.328378	1
Mn14	Mn	1	0.463851	0.139806	0.332561	1
Mn15	Mn	1	0.068047	0.725841	0.333855	1
Mn16	Mn	1	0.56806	0.726307	0.333136	1
Mn17	Mn	1	0.09868	0.60556	-0.00104	1
Mn18	Mn	1	0.597019	0.607184	-0.0011	1

Mn19	Mn	1	0.36759	0.526891	0.333029	1
Mn20	Mn	1	0.866758	0.525956	0.33717	1
Mn21	Mn	1	0.930694	0.273699	0.666218	1
Mn22	Mn	1	0.428975	0.275069	0.666668	1
Mn23	Mn	1	0.26385	0.93989	0.332488	1
Mn24	Mn	1	0.763537	0.940239	0.33187	1
O1	O	1	0.045388	0.222141	0.939736	1
O2	O	1	0.547085	0.223124	0.93769	1
O3	O	1	0.168315	0.981107	0.065554	1
O4	O	1	0.669909	0.985335	0.063455	1
O5	O	1	0.329298	0.01425	0.93636	1
O6	O	1	0.831478	0.017713	0.934392	1
O7	O	1	0.452642	0.775865	0.06154	1
O8	O	1	0.954195	0.776193	0.060517	1
O9	O	1	0.052307	0.390317	0.063971	1
O10	O	1	0.55344	0.388439	0.061399	1
O11	O	1	0.247237	0.408892	0.938876	1
O12	O	1	0.748466	0.410993	0.937568	1
O13	O	1	0.347751	0.199756	0.06467	1
O14	O	1	0.843869	0.204998	0.065863	1
O15	O	1	0.15587	0.793494	0.933925	1
O16	O	1	0.652056	0.799564	0.935274	1
O17	O	1	0.251849	0.587811	0.06184	1
O18	O	1	0.751977	0.589904	0.060998	1
O19	O	1	0.446197	0.611046	0.937651	1
O20	O	1	0.9472	0.608456	0.936327	1
O21	O	1	0.079281	0.077325	0.605936	1
O22	O	1	0.579379	0.079129	0.605116	1
O23	O	1	0.884554	0.058402	0.730671	1
O24	O	1	0.385336	0.056383	0.72934	1
O25	O	1	0.180659	0.866386	0.731206	1
O26	O	1	0.676769	0.872678	0.732463	1
O27	O	1	0.987525	0.462146	0.601641	1
O28	O	1	0.483934	0.467527	0.602743	1
O29	O	1	0.27847	0.278778	0.605352	1
O30	O	1	0.77925	0.277334	0.603301	1
O31	O	1	0.083762	0.255497	0.729505	1
O32	O	1	0.584542	0.257572	0.728377	1
O33	O	1	0.878421	0.890585	0.605937	1
O34	O	1	0.379725	0.890001	0.605461	1
O35	O	1	0.161274	0.681612	0.603253	1
O36	O	1	0.66438	0.685742	0.602085	1
O37	O	1	0.000582	0.649475	0.732598	1
O38	O	1	0.503301	0.65234	0.730854	1

O39	O	1	0.284092	0.44381	0.729381	1
O40	O	1	0.786059	0.444072	0.727974	1
O41	O	1	0.118156	0.110804	0.393352	1
O42	O	1	0.619985	0.108489	0.395745	1
O43	O	1	0.11368	0.942724	0.268573	1
O44	O	1	0.611569	0.944642	0.271807	1
O45	O	1	0.417363	0.920245	0.395306	1
O46	O	1	0.918935	0.92371	0.393703	1
O47	O	1	0.414816	0.745608	0.270115	1
O48	O	1	0.913493	0.742146	0.2717	1
O49	O	1	-0.00443	0.351739	0.267448	1
O50	O	1	0.497211	0.345821	0.269182	1
O51	O	1	0.013683	0.533889	0.398216	1
O52	O	1	0.51085	0.537133	0.397006	1
O53	O	1	0.320835	0.129207	0.268702	1
O54	O	1	0.817117	0.132497	0.267623	1
O55	O	1	0.333886	0.321443	0.396345	1
O56	O	1	0.835501	0.313411	0.397778	1
O57	O	1	0.210914	0.5578	0.270009	1
O58	O	1	0.713979	0.555939	0.272115	1
O59	O	1	0.219998	0.721865	0.393799	1
O60	O	1	0.718672	0.723049	0.397116	1
Li1	Li	1	0.49983	-0.00054	0.000141	1
Li2	Li	1	-6.7E-05	-0.00064	-0.00015	1
Li3	Li	1	0.332103	0.667265	0.666682	1
Li4	Li	1	0.832019	0.667372	0.667064	1
Li5	Li	1	0.165576	0.333614	0.33303	1
Li6	Li	1	0.665995	0.332474	0.333117	1

Note: Atomic positions in P3-NLM were not refined because the structure has low symmetry (P1) and contains a large number of atoms.

Tab. S2 Rietveld refinement results of XRD data for O3-LLM RT

Overall Composition: $Li_{0.59}[Li_{0.20}Mn_{0.82}]O_2$<br>Space group: $R\bar{3}m$; a = 2.837(2); c = 14.49(1); alpha = 90; gamma = 120; volume = 101.3(17)<br>wR = 7.676; GOF = 3.23							
label	site	multiplicity	x	y	z	fraction	Uiso
Li1	3b	3	0	0	0.5	0.58(6)	0.022(4)
Mn1	3a	3	0	0	0	0.82(7)	0.014(1)
O1	6c	6	0	0	0.2662(7)	1	0.026(5)
Li2	3a	3	0	0	0	0.18(7)	0.014(1)

Tab. S3 Rietveld refinement results of XRD data for O3-LLM HT

Overall Composition: $Li_{0.61}[Li_{0.19}Mn_{0.77}]O_2$<br>Space group: $R\bar{3}m$; a = 2.842(17); c = 14.38(1); alpha = 90; gamma = 120; volume = 101.3(12)<br>wR = 7.16; GOF = 2.93							
label	site	multiplicity	x	y	z	fraction	Uiso
Li1	3b	3	0	0	0.5	0.610	0.063(6)
Mn2	3a	3	0	0	0	0.77(5)	0.012(4)
O3	6c	6	0	0	0.262(1)	1.000	0.033(8)
Li2	3a	3	0	0	0	0.19(5)	0.012(4)
Mn1	3b	3	0	0	0.5	0.03(1)	0.063(6)

Tab. S4 The ICP-OES results of the P3-NLM sample before and after the different ion-exchange pathways. The Na/Li content changes are shown, with the Mn content fixed at 0.8 to determine the relative concentrations of other elements.

Samples	Na	Li	Mn
P3-NLM	0.617	0.173	0.8
O3-LLM RT	0.044	0.762	0.8
O3-LLM HT	0	0.784	0.8

Tab. S5 Rietveld refinement results of XRD data for LLM heated to 450 °C for 6h.

Overall Composition: $Li_{0.61}Mn_{0.1}[Li_{0.19}Mn_{0.68}]O_2$<br>Space group: $R\bar{3}m$; a = 2.86(17); c = 14.15(1); alpha = 90; gamma = 120; volume = 100.3(12)<br>wR = 4.727; GOF = 1.43							
label	site	multiplicity	x	y	z	fraction	Uiso
Li1	3b	3	0	0	0.5	0.610	0.68(6)
Mn2	3a	3	0	0	0	0.68(5)	0.022(4)
O3	6c	6	0	0	0.262(1)	1.000	0.045(8)
Li2	3a	3	0	0	0	0.19(5)	0.0217(4)
Mn1	3b	3	0	0	0.5	0.1(1)	0.68(6)

Tab. S6 Rietveld refinement results of XRD data for $Li_{1.2}Ni_{0.13}Co_{0.13}Mn_{0.54}O_2$ (O3-LNCM)

Overall Composition: $Li_{1.2}Ni_{0.13}Co_{0.13}Mn_{0.54}O_2$						
wR = 2.228; GOF = 2.16; 3% Li/Ni site exchange						
atom	site	x	y	z	fraction	U_{iso}
Phase 1[a] Composition: $Li_{1.12}Ni_{0.17}Co_{0.17}Mn_{0.51}O_2$ Space Group: $R\bar{3}m$.						
Li1	3b	0	0	0	0.969	0.01
Ni1	3b	0	0	0	0.031	0.01
Co1	3a	0	0	0.5	0.127	0.01
Mn1	3a	0	0	0.5	0.535	0.01
Ni2	3a	0	0	0.5	0.127	0.01
Li2	3a	0	0	0.5	0.211	0.01
O1	6c	0	0	0.248(5)	1	0.01
Phase 2[b] Composition: Li_2MnO_3. Crystal System: Monoclinic. Space Group: $C2/m$.						
atom	site	x	y	z	fraction	U_{iso}
Li	4h	0	0.338(5)	0.5	1	0.01
Li	2c	0	0	0.5	1	0.01
Li	2b	0	0.5	0	1	0.01
Mn	4g	0	0.166(8)	0	1	0.01
O	8j	0.256(4)	0.145(9)	0.810(2)	1	0.01
O	4i	0.324(2)	0	0.149(5)	1	0.01

[a]Lattice constants: a = b = 2.844(39) Å, c= 14.24(17) Å, V = 99.8(19) Å^3. [b]Lattice constants: a = 4.93(65) Å, b = 8.54(16) Å, c= 5.01(6) Å, V = 199.5(55) Å^3.

Tab. S7 Summaries on the electrochemical performances of reported LRMOs modified by different strategies.

Sample label	Composition and electrolyte	Modification strategies	Mass loading	Volume of the electrolyte	Applied currents	Voltage range	Half-cell performances	Refs.
1	O3-$Li_{1.2}Ni_{0.13}Co_{0.13}Mn_{0.54}O_2$ 1 **M** $LiPF_6$ solution in EC/DMC (weight ratio of 1:1)	Oxygen vacancies are introduced into the ≈10 nm-thick surface region of the cathode particle through prolonged medium-temperature annealing.	n/a	n/a	0.5 C	2–4.8 V	cn: 200 cr: 86.6%	[29]
2	O3-$Li_{1.2}Ni_{0.13}Co_{0.13}Mn_{0.54}O_2$ 1.0 **M** $LiPF_6$ in EC:DMC (volume ratio of 1:1)	Raw material	n/a	n/a	0.3 C	2–4.8 V	cn: 200 cr: 85%	[30]
3	$Li_{1.8}Mg_{0.3}Mn_{0.9}O_3$ (monoclinic) 1.0 **M** $LiPF_6$ in EC:DMC (volume ratio of 3:7)	Partially substituting Li^+ and Mn^{4+} of Li_2MnO_3 with the redox inactive Mg^{2+} as a structural stabilizer	2 mg cm^{-2}	n/a	4 C	2–4.8 V	cn: 200 cr: 72%	[31]
4	O2-$Li_{1.2}Ni_{0.13}Co_{0.13}Mn_{0.54}O_2$ 1.0 **M** $LiPF_6$ in FEC:FEMC:HFE (volume ratio of 2:6:2) + LiDFOB	all-fluorinated electrolyte	7 mg cm^{-2}	n/a	2/3 C	2–4.8 V	cn: 100 cr: 83.3%	[32]
5	O3-$Li_{1.2}Ni_{0.13}Fe_{0.13}Mn_{0.54}O_2$ 1.0 **M** $LiPF_6$ in EC:EMC:DMC (volume ratio of 1:1:1)	the robust fluoride coating layer and functional doping	2 mg cm^{-2}	n/a	0.2 C	2–4.8 V	cn: 120 cr: 90.1%	[33]
6	O3-$Li_{1.2-\sigma}Mn_{0.6}Ni_{0.2}O_2$ 1.0 **M** $LiPF_6$ in EC:DMC (volume ratio of 3:7)	Create an Li-deficient state in a Co-free lithium-rich cathode using a simple calcination-mediated surface-corrosion (CMISC) strategy.	n/a	50 µL	1 C	2–4.8 V	cn: 200 cr: 82.0%	[34]
7	O3-$Li_{1.2}Mn_{0.56}Ni_{0.16}Co_{0.08}O_2$ 1.0 **M** $LiPF_6$ in EC:DMC:DEC	The gradient W-doping mitigates crystal disintegration	2 mg cm^{-2}	n/a	0.5 C	2.5–4.6 V	cn: 200 cr: 83.2%	[35]

	(volume ratio of 1:1:1)	and enhances interfacial stability by forming a Li_2WO_4 coating layer on the material surface.						
8	O3-$Li_{1.2}Mn_{0.6}Ni_{0.2}O_2$<br>1.0 **M** $LiPF_6$ in EC:EMC:DMC<br>(volume ratio of 1:1:1)	Encapsulated cathode material in an N-doped graphene carbon matrix	3.5 mg cm^{-2}	n/a	0.2 C	2–4.8 V	cn: 200<br>cr: 86%	[36]
9	O3-$Li_{1.17}Mn_{0.44}Ni_{0.35}Co_{0.04}O_2$<br>n/a	high Ni content Li-rich cathode	4-5 mg cm^{-2}	n/a	1.0 C	2–4.8 V	cn: 200<br>cr: 72.29%	[37]
10	Spherical full gradient LNCM<br>1.0 **M** $LiPF_6$ in EC:PC:EMC:DEC<br>+ FEC+PS	gradient distributions of transition-metal ions, in which the Ni content increases continuously, Mn content decreases gradually and Co keeps at a low level.	2 mg cm^{-2}	n/a	2.0 C	2–4.8 V	cn: 100<br>cr: 84%	[38]
11	$Li_{4/7}[\square_{1/7}Mn_{6/7}]O_2$<br>1 **M** $LiClO_4$ in PC	Li-O-vacancy configuration	n/a	n/a	1.0 C	2–4.8 V	cn: 500<br>cr: 62%	[39]
12	$Li_{0.700}[Li_{0.222}Mn_{0.756}]O_2$<br>n/a	Ions exchange in molten salt at 280 °C for 4 h	n/a	n/a	1.0 C	2–4.8 V	cn: 100<br>cr: 80%	[23]
13	$Li_{1.17}Ni_{0.14}Co_{0.14}Mn_{0.55}O_2$<br>1 **M** $LiPF_6$ solution in EC/DMC<br>(weight ratio of 1:1)	the conversion of layered structure into a $LiLaO_2$ layer and Spinel phase structure	3 mg cm^{-2}	n/a	1.0 C	2–4.8 V	cn: 200<br>cr: 82.1%	[40]
14	O2-$Li_{0.6}Li_{0.2}Ru_{0.8}O_2$<br>1 **M** $LiPF_6$ solution in EC/DMC<br>(weight ratio of 1:1)	Ion exchange via molten salt method at 280°C	3 mg cm^{-2}	n/a	0.5 C	2.2-4.7 V	cn: 100<br>cr: 82.1%	[41]
15	O2-$Li_{0.85\square 0.15}[Li_{0.08\square 0.04}Ni_{0.22}Mn_{0.66}]O_2$<br>1 **M** $LiPF_6$ solution in EC/DEC<br>(weight ratio of 1:1)	Ion exchange via molten salt method at 280°C	2-3 mg cm^{-2}	n/a	0.1 C	2–4.8 V	cn: 100<br>cr: 95.8 %	[42]
16	O3-$Li_{4/7}[\square_{1/7}Ti_{1/7}Mn_{5/7}]O_2$<br>LB-111 purchased from DoDoChem	Ti-doped $Na_2Mn_3O_7$ underwent ion exchange via the molten salt method at 280 °C	n/a	n/a	1 C	2–4.8 V	cn: 100<br>cr: 86.1 %	[43]

17	O2+T2-$Li_{0.693}[Li_{0.153}Ni_{0.190}Mn_{0.657}]O_2$<br>1 **M** $LiPF_6$ solution in EC/DMC<br>(weight ratio of 1:1)	Ion exchange via molten salt method at 280°C	2 mg cm^{-2}	40 μL	0.2 C	2–4.8 V	cn: 50<br>cr: 97.4 %	[44]
18	O2/O3-$Li_{0.969}Ni_{0.225}Mn_{0.675}O_2$<br>1.0 **M** $LiPF_6$ dissolved in EC (ethylene carbonate):EMC (ethyl methyl carbonate)=3:7, vol%	Ion exchange via molten salt method at 290°C	n/a	n/a	0.1C	2–4.8 V	cn: 100<br>cr: 94.5 %	[45]
**This work**	**O3-$Li_{0.6}[Li_{0.2}Mn_{0.8}]O_2$<br>1.0 M $LiPF_6$ in EC:DMC:DEC<br>(volume ratio of 1:1:1)**	**Ions exchange at RT**	**2 mg cm^{-2}**	**100 ul**	**0.2 C**	**2–4.8 V**	**cn: 400<br>cr: 88.1%**	

Note: cr: capacity retention, cn: cycling number.

Tab. S8 Mn K-edge EXAFS fitting results on powder pellet samples of P3-NLM, O3-LLM (RT and HT), Spinel-LLM, and reference compounds including Li_2MnO_3, Mn_2O_3, and MnO_2.

Sample	Scattering path	CN	R[Å]	ΔR[Å]	DW factor [Å^2]
Li_2MnO_3	Mn-O(1st)	4.59(65)	1.899(12)	+0.030(12)	0.00168(15)
	Mn-Mn(2nd)	2.12(44)	2.854(18)	+0.053(18)	0.00300(19)
O3-LLM RT	Mn-O(1st)	4.55(105)	1.886(19)	-0.051(19)	0.00141(30)
	Mn-Mn(2nd)	3.24(132)	2.867(27)	+0.024(27)	0.00626(37)
O3-LLM HT	Mn-O(1st)	4.50(53)	1.883(9)	-0.138(9)	0.00405(14)
	Mn-Mn(2nd)	3.30(79)	2.865(13)	+0.064(13)	0.00624(22)
Spinel-LLM	Mn-O(1st)	4.37(59)	1.890(11)	-0.131(11)	0.00347(17)
	Mn-Mn(2nd)	2.95(85)	2.877(13)	+0.076(13)	0.00385(22)
P3-NLM	Mn-O(1st)	4.51(59)	1.888(10)	-0.066(10)	0.00400(16)
	Mn-Mn(2nd)	3.75(92)	2.885(14)	+0.048(14)	0.00650(23)
Mn_2O_3	Mn-O(1st)	2.00 (fixed)	1.941(15)	+0.023(15)	0.00176(172)
	Mn-O(2nd)	4.00 (fixed)	2.200(38)	-0.032(38)	0.03254(867)
	Mn-Mn(3rd)	9.15(358)	3.154(14)	+0.037(14)	0.01009(260)
MnO_2	Mn-O(1st)	4.58(57)	1.886(11)	−0.011(11)	0.00194(15)
	Mn-Mn(2nd)	2.50 (fixed)	2.873(25)	+0.028(25)	0.00564(63)
	Mn-Mn(3rd)	2.50 (fixed)	3.426(17)	+0.010(12)	0.00097(24)

Note: EXAFS data were fitted in R-space using Artemis/IFEFFIT with theoretical FEFF paths. The fitting was performed over a k-range of 3-13 Å^{-1} and R-range of 1-3.6 Å, using a Hanning window and k-weights of 1, 2, and 3. Coordination shells (Mn-O and Mn-Mn) were included. For Mn_2O_3, the first Mn-O shell was fitted using a single averaged shell model to enable direct comparison of average Mn-O bond lengths among different samples. This yields an effective Mn-O distance of ~1.96 Å, which underestimates the true coordination number due to the Jahn-Teller distortion of Mn^{3+}. A split-shell fit (4+2) gives short-axis ~1.88–1.95 Å and long-axis ~2.10-2.20 Å; this does not alter the trend of Mn-O bond lengths among the samples. For MnO_2, the CN for the Mn-Mn second and third shells were fixed because the EXAFS amplitude is highly correlated with the Debye-Waller factor, and the higher-shell signals are weak in the k-range used. Similarly, fixing CN in Mn_2O_3 ensures physically reasonable values and allows accurate determination of Mn-O bond distances. Overall, fixing CN in these cases prevents unphysical fits while preserving reliable trends in Mn-O bond lengths across samples.

Tab. S9 Semi-quantitative analysis of normalized O K-edge peak-area ratios (R) for different electrodes at various electrochemical states

Material	State	$R=A_{pre}/A_{main}$	R retention (%)	R decrease (%)
O3-LLM RT	Pristine	0.2605	100.0%	0.0%
	1 cycle	0.2502	96.0%	4.0%
	250 cycles	0.2413	92.6%	7.4%
O3-LLM HT	Pristine	0.2937	100.0%	0.0%
	1 cycle	0.2595	88.3%	11.7%
	250 cycles	0.2454	83.5%	16.5%
Spinel-LLM	Pristine	0.2858	100.0%	0.0%
	1 cycle	0.2493	87.2%	12.8%
	200 cycles	0.2082	72.8%	27.2%

Note: The pre-edge (528-533 eV) and main-edge (535-550 eV) regions were integrated to obtain the peak-area ratio $R = \frac{A_{pre-edge}}{A_{main-edge}}$, which serves as a semi-quantitative descriptor of oxygen-related electronic-state evolution during cycling.

References

1. Toby BH, Von Dreele RB. GSAS-II: the genesis of a modern open-source all purpose crystallography software package. J Appl Cryst 2013; 46: 544-49.

2. Gann E, McNeill CR, Tadich A *et al.* Quick AS NEXAFS Tool (QANT): a program for NEXAFS loading and analysis developed at the Australian Synchrotron. J Synchrotron Rad 2016; 23: 374-80.

3. Ravel B, Newville M. ATHENA, ARTEMIS, HEPHAESTUS: data analysis for X-ray absorption spectroscopy using IFEFFIT. J Synchrotron Rad 2005; 12: 537-41.

4. Munoz M, Argoul P, Farges F. Continuous Cauchy wavelet transform analyses of EXAFS spectra: A qualitative approach. Am Mineral 2003; 88: 694-700.

5. Funke H, Scheinost AC, Chukalina M. Wavelet analysis of extended x-ray absorption fine structure data. Phys Rev B 2005; 71: 094110.

6. D.Massiot, F.Fayon, M.Capron *et al.* Modelling one and two-dimensional solid-state NMR spectra. Magn Reson Chem 2002; 40: 70-6.

7. Kresse G, Furthmuller J. Efficiency of ab-initio total energy calculations for metals and semiconductors using a plane-wave basis set. Comp Mater Sci 1996; 6: 15-50.

8. Blochl PE. Projector augmented-wave method. Phys Rev B 1994; 50: 17953-79.

9. Kresse G, Joubert D. From ultrasoft pseudopotentials to the projector augmented-wave method. Phys Rev B 1999; 59: 1758.

10. Perdew JP, Burke K, Ernzerhof M. Generalized gradient approximation made simple. Phys Rev Lett 1997; 78: 1396.

11. Grimme S, Ehrlich S, Goerigk L. Effect of the damping function in dispersion corrected density functional theory. J Comput Chem 2011; 32: 1456-65.

12. Grimme S, Antony J, Ehrlich S *et al.* A Consistent andaccurate ab initio parametrization of density functional dispersion correction (DFT-D) for the 94 elements H-Pu. J Chem Phys 2010; 132: 154104.

13. Seo DH, Lee J, Urban A *et al.* The structural and chemical origin of the oxygen redox activity in layered and cation-disordered Li-excess cathode materials. Nat Chem 2016; 8: 692-7.

14. Momma K, Izumi F. VESTA 3 for three-dimensional visualization of crystal, volumetric and morphology data. J Appl Cryst 2011; 44: 1272-76.

15. Ong SP, Richards WD, Jain A *et al.* Python materials genomics (Pymatgen): a robust, open-source

python library for materials analysis. Comp Mater Sci 2013; 68: 314-19.
16. Deringer VL, Tchougréeff AL, Dronskowski R. Crystal orbital hamilton population (COHP) analysis as projected from plane-wave basis sets. J Phys Chem A 2011; 115: 5461-66.
17. Dronskowski R, Bloechl PE. Crystal orbital Hamilton populations (COHP): energy-resolved visualization of chemical bonding in solids based on density-functional calculations. J Phys Chem Lett 1993; 97: 8617-24.
18. Maintz S, Deringer VL, Tchougréeff AL *et al.* LOBSTER: A tool to extract chemical bonding from plane-wave based DFT. J Comput Chem 2016; 37: 1030-35.
19. Henkelman G, Jónsson H. Improved tangent estimate in the nudged elastic band method for finding minimum energy paths and saddle points. J Chem Phys 2000; 113: 9978-9985.
20. Henkelman G, Uberuaga BP, Jónsson H. A climbing image nudged elastic band method for finding saddle points and minimum energy paths. J Chem Phys 2000; 113: 9901-04.
21. McColl K, Coles SW, Zarabadi-Poor P *et al.* Phase segregation and nanoconfined fluid O_2 in a lithium-rich oxide cathode. Nat Mater 2024; 23: 826-33.
22. Hoover WG. Canonical dynamics: Equilibrium phase-space distributions. Phys Rev A 1985; 31: 1695-97.
23. Song J, Li B, Chen Y *et al.* A high-performance Li–Mn–O Li-rich cathode material with rhombohedral symmetry via intralayer Li/Mn disordering. Adv Mater 2020; 32: 2000190.
24. Dogan F, Long BR, Croy JR *et al.* Re-entrant lithium local environments and defect driven electrochemistry of Li- and Mn-rich Li-ion battery cathodes. J Am Chem Soc 2015; 137: 2328-35.
25. Li B, Zhuo Z, Zhang L *et al.* Decoupling the roles of Ni and Co in anionic redox activity of Li-rich NMC cathodes. Nat Mater 2023; 22: 1370-79.
26. Nguyen H, Clément RJ. Rechargeable batteries from the perspective of the electron spin. ACS Energy Lett 2020; 5: 3848-59.
27. Tang M, Dalzini A, Li X *et al.* Operando EPR for simultaneous monitoring of anionic and cationic redox processes in Li-rich metal oxide cathodes. J Phys Chem Lett 2017; 8: 4009-16.
28. Banerjee A, Shilina Y, Ziv B *et al.* On the oxidation state of manganese ions in Li-ion battery electrolyte solutions. J Am Chem Soc 2017; 139: 1738-41.
29. Wang K, Qiu J, Hou F *et al.* Unraveling the role of surficial oxygen vacancies in stabilizing Li-

rich layered oxides. Adv Energy Mater 2023; 13: 2301216.

30. Wang L, Liu T, Dai A *et al.* Reaction inhomogeneity coupling with metal rearrangement triggers electrochemical degradation in lithium-rich layered cathode. Nat Commun 2021; 12: 5370.

31. Lee Y, Park H, Cho Mk *et al.* Li-rich Mn–Mg layered oxide as a novel Ni-/Co-free cathode. Adv Funct Mater 2022; 32: 2204354.

32. Cui C, Fan X, Zhou X *et al.* Structure and interface design enable stable Li-rich cathode. J Am Chem Soc 2020; 142: 8918-27.

33. Zhao H, Li W, Li J *et al.* Enhance performances of Co-free Li-rich cathode by eutesctic melting salt treatment. Nano Energy 2022; 92: 106760.

34. Zhang Y, Chen Z, Shi X *et al.* Regulation of 3d-transition metal interlayered disorder by appropriate lithium depletion for Li-rich layered oxide with remarkably enhanced initial coulombic efficiency and stability. Adv Energy Mater 2022; 13: 2203045.

35. Meng J, Xu L, Ma Q *et al.* Modulating crystal and interfacial properties by W-gradient doping for highly stable and long life Li-rich layered cathodes. Adv Funct Mater 2022; 32: 2113013.

36. Chen M, Zhang G, Wu B *et al.* N-Doped Graphene-Modified Li-Rich Layered $Li_{1.2}Mn_{0.6}Ni_{0.2}O_2$ Cathode for High-Performance Li-Ion Batteries. ACS Appl Energy Mater 2022; 5: 4307-17.

37. Sun G, Yu F-D, Que L-F *et al.* Local electronic structure modulation enhances operating voltage in Li-rich cathodes. Nano Energy 2019; 66: 104102.

38. Ju X, Hou X, Liu Z *et al.* The full gradient design in Li-rich cathode for high performance lithium ion batteries with reduced voltage decay. J Power Sources 2019; 437: 226902.

39. Cao X, Li H, Qiao Y *et al.* Reversible anionic redox chemistry in layered $Li_{4/7}[\square_{1/7}Mn_{6/7}]O_2$ enabled by stable Li–O-vacancy configuration. Joule 2022; 6: 1290-303.

40. Li S, Liu F, Zhang S *et al.* Surface nonbonding oxygen state regulation for reversible anionic redox chemistry in Li-rich Mn-based layered oxides. Angew Chem Int Ed 2025; n/a: e202424079.

41. Cui T, Xu J, Wang X *et al.* Highly reversible transition metal migration in superstructure-free Li-rich oxide boosting voltage stability and redox symmetry. Nat Commun 2024; 15: 4742.

42. Yang X, Wang K, Zhang J *et al.* Modulating local oxygen coordination to achieve highly reversible anionic redox and negligible voltage decay in O2-type layered cathodes for Li-ion batteries. Adv Energy Mater 2025; 15: 2404276.

43. Zhao X, Roy I, Sheng C *et al.* Tailoring oxygen redox activity and accelerating Li^+ diffusion via Ti substitution within Li-O-vacancy configurations. Adv Funct Mater 2025; n/a: 2504973.
44. Wang Y, Li C, Li Y *et al.* A metastable oxygen redox cathode for lithium-ion batteries. Angew Chem Int Ed 2025; 64: e202422789.
45. Wang C, Zhang Z, Zheng Y *et al.* Constructing face-shared configuration at the hetero-interface in Li-rich layered oxide cathodes. Angew Chem Int Ed 2026; n/a: e2943336.
